# Consolidative stereotactic radiotherapy for oligo-residual non-small cell lung cancer after first-line chemoimmunotherapy: A single-arm, phase 2 trial from China

**DOI:** 10.1371/journal.pmed.1004680

**Published:** 2025-08-01

**Authors:** Hongru Chen, Lu Yu, Fei Liang, Yue Zhou, Li Chu, Xiao Chu, Xi Yang, Junhua Zhang, Yechun Pang, Zezhou Wang, Zhiyong Yuan, Jianjiao Ni, Zhengfei Zhu

**Affiliations:** 1 Department of Radiation Oncology, Fudan University Shanghai Cancer Center, Shanghai, China; 2 Department of Oncology, Shanghai Medical College, Fudan University, Shanghai, China; 3 Shanghai Clinical Research Center for Radiation Oncology, Shanghai, China; 4 Shanghai Key Laboratory of Radiation Oncology, Shanghai, China; 5 Department of Radiation Oncology, Tianjin Medical University Cancer Institute and Hospital, National Clinical Research Center for Cancer, Tianjin, China; 6 Department of Biostatistics, Zhongshan Hospital, Fudan University, Shanghai, China; 7 Department of Cancer Prevention, Fudan University Shanghai Cancer Center, Shanghai, China; 8 Institute of Thoracic Oncology, Fudan University, Shanghai, China; Washington University in St Louis, UNITED STATES OF AMERICA

## Abstract

**Background:**

Retrospective evidence indicated potential survival benefit of consolidative stereotactic radiotherapy (SRT) in patients with metastatic driver mutation-negative non-small cell lung cancer (NSCLC) harboring oligo-residual disease (ORD) after effective immune checkpoint inhibitor treatment. However, prospective data about consolidative SRT in this disease population after first-line chemoimmunotherapy remains scarce.

**Methods and findings:**

From March 2021 to March 2023, 59 patients (94.92% males) with metastatic driver mutation-negative NSCLC harboring ORD after effective first-line chemoimmunotherapy were enrolled in this single-arm, phase 2 trial (NCT04767009), which was conducted at Fudan University Shanghai Cancer Center, Shanghai, China. The median (interquartile range) age was 64 (57,71) years. All of the patients received extracranial and/or cranial SRT covering all of the oligo-residual lesions, without holding the maintenance systemic therapy during SRT. The most common sites targeted by consolidative SRT included the lung (*n* = 30), lymph nodes (*n* = 26), bone (*n* = 22), and brain (*n* = 22). All efficacy and safety analyses followed the intention-to-treat principle with all 59 enrolled patients included. No patient was lost to follow-up. The primary endpoint was progression-free survival (PFS). Secondary endpoints included overall survival (OS) and treatment-related adverse events (TRAEs). With a median follow-up of 14.8 months, the median PFS was 29.0 (90% CI [13.97, Not Reach]) months, meeting the primary endpoint. The 2-year OS rate was 88.9% (95% CI [75.9%,100%]). TRAEs of any grade and grade ≥3 occurred in 58 (98.31%) and 13 (22.03%) patients, respectively. Moreover, a prespecified propensity score-matched comparison was conducted with a contemporary cohort of patients who developed ORD but received systematic therapy alone, which found that incorporating consolidative SRT was associated with prolonged PFS (adjusted HR 0.286, *P* < 0.001) and OS (adjusted HR 0.229, *P* = 0.023). The main methodological limitation of this single-arm trial is its inability to establish causal relationships and the findings require validation in randomized controlled trials.

**Conclusions:**

Consolidative SRT was associated with prolonged PFS and generally acceptable toxicities in first-line chemoimmunotherapy-treated patients with metastatic NSCLC harboring ORD, supported by propensity-matched comparisons with a contemporary cohort.

## Author summary

**Why was this study done?:**

- Chemoimmunotherapy is the first-line treatment for advanced non-small cell lung cancer (NSCLC) without driver mutations, but resistance often develops, leading to disease progression.
- Prior retrospective studies suggested consolidative stereotactic radiotherapy (SRT) may improve survival in patients with driver mutation-negative NSCLC harboring oligo-residual disease (ORD) after effective immune checkpoint inhibitor therapy, but prospective evidence remains limited.

**What did the researchers do and find?:**

- In this single-arm trial, 59 patients with ORD after effective chemoimmunotherapy received consolidative SRT to all residual tumors. After a median follow-up of 14.8 months, median progression-free survival was 29 months—nearly triple the historical control (10.0 months)—and the 2-year overall survival rate was 88.9%.
- Compared to matched patients receiving chemoimmunotherapy alone, adding SRT prolonged patient’s survival with generally acceptable toxicities.

**What do these findings mean?:**

- Consolidative SRT shows promise for extending survival in patients with chemoimmunotherapy-responsive NSCLC with oligo-residual disease, supporting its integration into treatment strategies.
- Limitations include the single-arm design, potential unmeasured confounding factors, and immature OS data.
- Confirmatory randomized trials are needed to validate these findings and refine patient selection, as well as SRT dose-fractionations.

## Abstract 3

In a single-arm trial, Hongru Chen, Lu Yu and colleagues investigate whether consolidative stereotactic radiotherapy improves survival in patients with driver mutation-negative NSCLC harboring oligo-residual disease after effective chemoimmunotherapy.

## Introduction

Immune checkpoint inhibitors (ICIs), targeting program death-1 (PD-1) or its ligand (PD-L1), have changed the treatment paradigm of non-small cell lung cancer (NSCLC). PD-1/PD-L1 inhibitors in combination with chemotherapy emerge as the first-line treatment for driver mutation-negative metastatic NSCLC, regardless of PD-L1 expression [1,2]. However, the development of primary and acquired resistance poses great threats to patients’ prognosis and quality of life, highlighting an urgent need to explore combinational treatments that can delay or overcome drug resistance [3,4].

Accumulating evidence suggested that appropriate local ablative therapy (LAT) could potentially improve treatment outcomes in ICI-treated patients with metastatic NSCLC, especially those with oligo-metastatic, oligo-residual, or oligo-progressive disease [5–8]. Theoretically, consolidative LAT may have advantages over concurrent LAT or salvage LAT, since higher tumor control rates could be achieved with fewer side effects at the time of maximal response to ICIs. Our previous retrospective studies identified 19.1% of ICI-treated patients with metastatic NSCLC harbored oligo-residual disease (ORD) at the maximal response and consolidative LAT could prolong patient’s survival [8]. Moreover, potential synergistic effects between extracranial and/or cranial radiotherapy, especially stereotactic radiotherapy (SRT), and ICIs in metastatic NSCLC have been demonstrated by our groups [9–11] and others [12,13]. However, prospective data on the safety and efficacy of consolidative SRT for chemoimmunotherapy-treated patients with metastatic NSCLC with ORD is still limited.

Hence, a prospective, single-arm, phase 2 trial (NCT04767009) was initiated to evaluate the safety and efficacy of consolidative SRT in driver mutation-negative metastatic NSCLC who harbored ORD after effective first-line chemoimmunotherapy. Additionally, a protocol-prespecified propensity score matched (PSM) comparison with a contemporary institutional cohort of patients who received first-line chemoimmunotherapy alone was conducted to better assess the clinical value of consolidative SRT.

## Methods

### Study design and participants

The detailed protocol of the current trial was provided in the S1 Protocol and the trial was approved by the Institutional Review Board and ethics committees of Fudan University Shanghai Cancer Center (No. 2012228-10-2306A), and registered with ClinicalTrials.gov (NCT04767009). The studies were conducted in accordance with the Declaration of Helsinki. All patients provided written informed consent to participate. Inclusion criteria included ≥18 and ≤75 years of age, Eastern Cooperative Oncology Group performance status of 0–1, pathologically confirmed stage IV NSCLC according to the American Joint Committee on Cancer staging manual (8th Edition), without common sensitizing driver mutations (including EGFR, ALK and ROS1). Full eligibility criteria are detailed in the S1 Protocol. Adequate baseline tumor assessment was required before the initiation of first-line chemoimmunotherapy and each patient should have at least one measurable lesion according to Response Evaluation Criteria in Solid Tumors, version 1.1. Patients should harbor ORD after effective first-line chemoimmunotherapy, defined as having partial response (PR) or durable stable disease (SD) (SD lasting no less than 6 months). The oligo-residual tumor lesions should be amenable to consolidative SRT in the opinion of the investigators. Exclusion criteria were history of or concurrent secondary malignancy, pregnancy or breastfeeding, general conditions that might affect compliance or ability to sign informed consent.

Notably, patients with cranial and/or extracranial ORD could be included. The definition of ORD was adjusted to the status of brain metastases (BMs). Extracranial ORD was defined as residual tumors limited to three organs and five lesions among those without baseline BMs or those with complete cranial response, in line with the EORTC-ESTRO consensus [14]. Of note, the primary tumors were also counted as involved lesions/organs. Otherwise, cranial ORD was defined as the BMs limited to 10 lesions with the largest tumor <10 mL in volume and <3 cm in longest diameter and total cumulative volume ≤15 mL, among those with residual BMs and without extracranial progressive disease (in this circumstance, extracranial ORD was not necessarily required), consistent with the JLGK0901 study [15]. Based on the Response Assessment in Neuro-Oncology standard, at least one BM lesion with a diameter >1 cm or a lesion with a diameter >0.5 cm on a 1.5 mm thick thin layer MRI was required for response evaluation [16].

### Procedures

Patients with driver mutation-negative NSCLC receiving first-line chemoimmunotherapy, consisting of commercially available PD-1/PD-L1 inhibitors and platinum-doublet chemotherapy, were evaluated for eligibility for consolidative SRT, which was scheduled after 4 cycles of chemoimmunotherapy and every 2–3 months for one year thereafter. Of note, the status of ORD was confirmed by PET/CT among potential candidates who were willing to participate.

Patients enrolled in this trial were treated with the intent to ablate all residual disease with consolidative SRT. Cranial SRT was prescribed at 27 Gy in three fractions [17]. The dose and fractionation for extracranial ORD were referred to the regimens used in the NRG-BR001 trial [18]. SRT was intentionally scheduled during the interval between cycles of maintenance chemoimmunotherapy to avoid treatment interruption. The maintenance immunotherapy was continued during and after consolidative SRT for up to 2 years or until disease progression or intolerable toxicity.

Follow-up visits were scheduled every three months for the first two years and every six months thereafter. Medical history, physical examination, chest CT scan, and abdominal ultrasound or CT scan were regularly assessed during each follow-up. Other tests, such as bone scanning and PET/CT, were performed at the discretion of the treating physicians. In patients without BMs, follow-up brain MRI scans were not mandatory and were performed at the discretion of treating physicians, typically after the appearance of indicative symptoms. In patients with BMs, brain MRI scans were regularly performed during each follow-up. Toxicity assessments were conducted at each follow-up.

### Outcomes

The primary endpoint was progression-free survival (PFS), which was defined as the time from the date of initiation of first-line chemoimmunotherapy to the time of disease progression or death. For patients who had no disease progression, PFS was censored on the date of the last follow-up.

Treatment related adverse events (TRAEs) and overall survival (OS) were secondary endpoints. TRAEs were evaluated and recorded using the Common Terminology Criteria for Adverse Events, version 5.0. OS was defined as the time from the date of initiation of first-line chemoimmunotherapy to death from any cause. For patients alive, OS was censored on the date of the last follow-up.

Exploratory endpoints included the predictive and prognostic values of distinct infiltrating immune cells in the baseline tumor tissues and various kinds of circulating biomarkers, such as cytokines and immune cell subsets. The exploratory endpoints will be reported separately.

### Prespecified propensity-score matched comparison with a contemporary cohort

In addition to reporting the endpoints of all enrolled patients, the protocol-prespecified PSM by a set of important factors was conducted with a contemporary cohort of patients with oligo-residual NSCLC after first-line chemoimmunotherapy but receiving systemic therapy alone during the period of enrollment. PSM analysis was undertaken in an attempt to adjust for potential bias associated with prognostic factors related to treatment.

The patients used to create this PSM comparison cohort were derived from a prospective observational study, which has been described in our previous studies [19,20]. This study was approved by the Institutional Review Board and ethics committees of Fudan University Shanghai Cancer Center (No. 2012228-3) and those of the other two participating centers, and was registered with ClinicalTrials.gov (NCT04766515). All patients provided written informed consent to participate. The initial aim of the prospective observational study was to further investigate the real-world efficacy and safety of ICI treatment in solid tumors, with a focus on metastatic NSCLC. Detailed information about patient inclusion/exclusion criteria, endpoints and procedures, and statistical analysis plans could be found in the relevant protocol provided in S2 Protocol.

### Statistical analysis

Due to the lack of prospective data about the survival outcomes of patients with metastatic NSCLC with ORD after effective first-line chemoimmunotherapy at the time of study design, a pooled analysis of retrospective studies from our group was conducted and found a median PFS of 10.0 months for those harboring extracranial and/or cranial ORD [8,11], which served as the historical control of the current trial. The sample size calculation was based on the primary endpoint of PFS. Assuming that the PFS in patients with metastatic NSCLC receiving first-line chemoimmunotherapy and harboring ORD was 10.0 months and that the addition of consolidative SRT would improve PFS with a hazard ratio (HR) of 0.60. All the participants would be enrolled in two years and the last participant would be followed up for at least one year. Based on a one-sided alpha of 0.05 and a power of 0.9, the total sample size was 53 patients. With a presumed dropout rate of 10% before the study evaluation, 59 patients were needed in this trial.

R package “MatchIt” was used for 1:1 nearest-neighbor PSM with age, sex, smoking history, Eastern Cooperative Oncology Group performance status, histology, PD-L1 expression, staging, number of involved lesions/organs, the status of baseline brain metastasis, the status of bone metastasis, and status of liver metastasis.

Continuous variables were summarized as medians with interquartile ranges (IQRs), and categorical variables were reported as counts and frequencies (*n*%). The chi-squared test for categorical variables, and *t* test or Wilcoxon Rank Sum test for continuous variables, were used. The confidence intervals (CIs) of PFS and OS were calculated by the Clopper–Pearson method. Of note, since a one-sided significance level (type I error rate) of 5% was adopted for the sample size calculation, a two-sided 90% CI was calculated for the primary endpoint (i.e., PFS). For other parameters, a two-sided 95% CI was used for the convenience of inter-study comparisons. The Kaplan-Meier method was used to estimate time-to-event outcomes such as PFS and OS, with comparisons made with the log-rank test. Univariate and multivariate Cox analyses were used to explore the association between clinical characteristics and PFS and OS in propensity score matched cohorts, and clinical characteristics with *P* < 0.10 in univariate analysis were included in multivariate analysis. SPSS (version 27) and R software (version 4.3.2) were used for all analyses, and statistical significance was set at *P* < 0.05 without adjustments for multiplicity.

This study is reported as per the CONSORT (Consolidated Standards of Reporting Trials) 2025 guideline (see S1 CONSORT Checklist).

## Results

**Fig 1:**
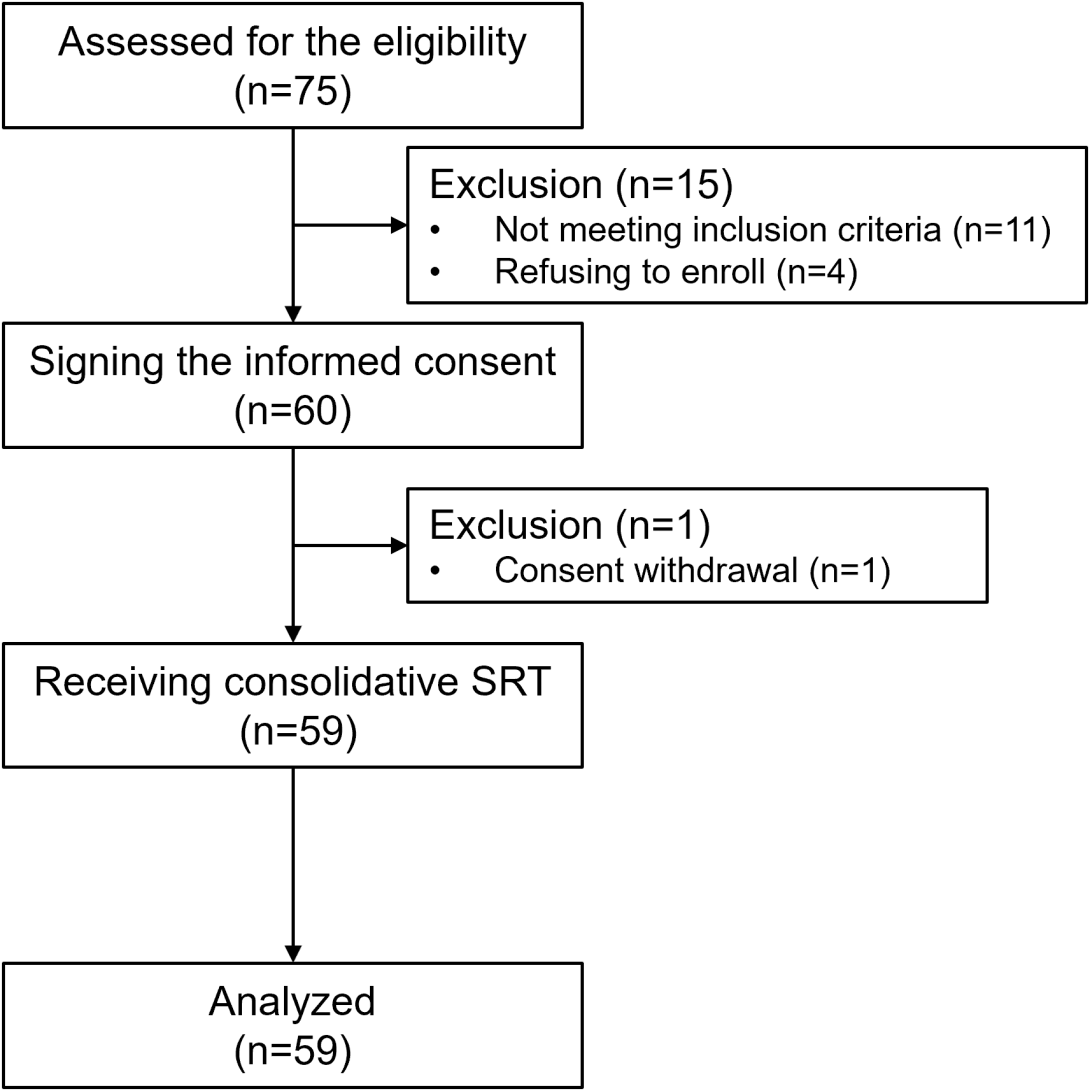
Enrollment flowchart. SRT, stereotactic radiotherapy.

The current trial enrollment started in March 1, 2021 and was completed in March 1, 2023, with the last follow-up date of June 30, 2024. The CONSORT flow diagram for this trial is shown in Fig 1. A total of 59 patients were enrolled and the baseline clinical characteristics were displayed in Table 1. Baseline metastasis in the brain, bone, and liver existed in 22 (37.3%), 22 (37.3%), and 3 (5.1%) patients, respectively. Additionally, the best response of PR and durable SD to the induction chemoimmunotherapy were documented in 40 (67.8%) and 19 (32.2%) patients, respectively. All of the patients received consolidative SRT, with a median interval between the diagnosis of metastatic disease to the initiation of consolidative SRT of 4.17 (IQR 3.27–8.30) months. Detailed information about radiation sites was described in S1 Table.

**Table 1:** Baseline characteristics.

Characteristic	CIT + SRT (*n* = 59)
Age, years	64 (57–71)
Age group	
<65 years	23 (38.98%)
≥65 years	36 (61.02%)
Sex	
Female	3 (5.08%)
Male	56 (94.92%)
Smoking status	
Never	13 (22.03%)
Current or previous	46 (77.97%)
ECOG PS	
0	29 (49.15%)
1	30 (50.85%)
Histology	
Non-squamous	34 (57.63%)
Squamous	25 (42.37%)
PD-L1 status	
<1%	23 (38.98%)
≥1% to <50%	15 (25.42%)
≥50%	16 (27.12%)
Unknown	5 (8.47%)
Stage	
IVA	33 (55.93%)
IVB	26 (44.07%)
Number of involved lesions	
1	29 (49.15%)
2–5	21 (35.59%)
>5	9 (15.25%)
Number of involved organs	
1	18 (30.51%)
2–3	38 (64.41%)
>3	3 (5.08%)
Brain metastasis	
Yes	22 (37.29%)
No	37 (62.71%)
Bone metastasis	
Yes	22 (37.29%)
No	37 (62.71%)
Liver metastasis	
Yes	3 (5.08%)
No	56 (94.92%)

Data are *n* (%) or median (IQR). CIT, chemoimmunotherapy. SRT, stereotactic radiotherapy. ECOG PS, Eastern Cooperative Oncology Group Performance Status.

**Fig 2:**
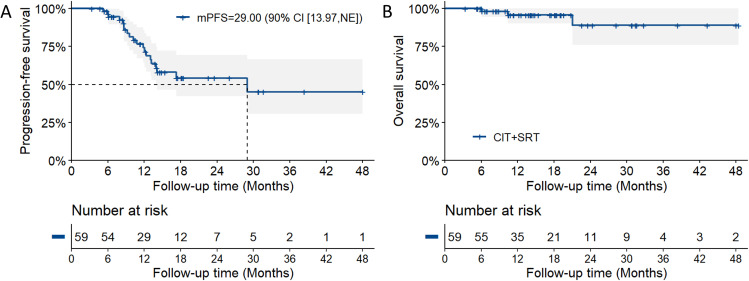
Survival outcomes in patients receiving consolidative SRT (*n* = 59). Progression-free survival (PFS) with 90% confidence interval (CI) **(A)** and overall survival (OS) with 95% CI **(B)**. NE, not evaluable. mPFS, median PFS.

With a median follow-up of 14.80 (IQR 10.27–23.53) months, 19 (32.2%) patients developed progressive disease (PD), including intracranial PD in 5 (8.5%) and extracranial PD in 14 (23.7%) patients. The subsequent treatments in these patients were described in S2 Table. The median PFS in all patients was 29.0 months [90% CI [13.97, Not Reach (NR)], Fig 2A], the lower boundary of which exceeded 10.0 months, and the current trial met the primary endpoint. The 1- and 2-year PFS rates were 74.2% (90% CI [64.3%,85.7%]) and 54.2% (90% CI [42.3%,69.4%]), respectively. Meanwhile, among the 22 (37.3%) patients who received cranial SRT for residual BMs, 8 (36.4%) patients developed initial PD [intracranial PD = 4 (18.2%), extracranial PD = 4 (18.2%)]. The median PFS in this subgroup was 17.3 months (95% CI [12.3, NR], S1 Fig) and 1- and 2-year PFS rates were 80.2% (95% CI [62.4%,100%]) and 42.5% (95% CI [22.8%,79.5%]), respectively.

At the time of the data lock, 3 (5.1%) patients died. The median OS had not been reached (95% CI NR) and the 2-year OS rate was 88.9% (95% CI [75.9%, 100%], Fig 2B). In the patients receiving cranial SRT for residual BMs, 2 (9.1%) patients died and the median OS had not been reached (95% CI NR, S1 Fig).

TRAEs of any grade were reported in 58 (98.31%) patients (Table 2). The most common TRAEs were pneumonitis (grade ≥ 2) (28.81%) and anemia (27.12%), followed by platelet count decrease (23.73%), neutrophil count decreased (23.73%), and nausea (22.03%). A total of 13 (22.03%) patients reported grade ≥ 3 TRAEs, including pneumonitis, anemia, platelet count decreased, neutrophil count decreased, fatigue, esophagitis, skin rash, aspartate aminotransferase increased, diarrhea, and alanine aminotransferase increased. None of the TRAEs led to death.

**Table 2:** Treatment-related adverse events (*n* = 59).

Event, *n* (%)	Any grades	Grade 3	Grade 4
Any TRAE	58 (98.31%)	11 (18.64%)	2 (3.39%)
Potential immune-related AEs	35 (59.32%)	7 (11.86%)	1 (1.69%)
TRAE leading to treatment discontinuation	11 (18.64%)	8 (13.56%)	1 (1.69%)
TRAE leading to death	0 (0.00%)	0 (0.00%)	0 (0.00%)
Pneumonitis (Grade 2 or higher)	17 (28.81%)	3 (5.08%)	1 (1.69%)
Anemia	16 (27.12%)	4 (6.78%)	0 (0.00%)
Platelet count decreased	14 (23.73%)	4 (6.78%)	1 (1.69%)
Neutrophil count decreased	14 (23.73%)	5 (8.47%)	2 (3.39%)
Nausea	13 (22.03%)	0 (0.00%)	0 (0.00%)
Fatigue	7 (11.86%)	2 (3.39%)	0 (0.00%)
Pyrexia	7 (11.86%)	0 (0.00%)	0 (0.00%)
Esophagitis	4 (6.78%)	2 (3.38%)	0 (0.00%)
Skin rash	4 (6.78%)	1 (1.69%)	0 (0.00%)
Diarrhea	3 (5.08%)	1 (1.69%)	0 (0.00%)
Anorexia	3 (5.08%)	0 (0.00%)	0 (0.00%)
Aspartate aminotransferase increased	2 (3.39%)	1 (1.69%)	0 (0.00%)
Pruritus	2 (3.39%)	0 (0.00%)	0 (0.00%)
Radiation dermatitis	2 (3.39%)	0 (0.00%)	0 (0.00%)
CNS radiation necrosis	2 (3.39%)	0 (0.00%)	0 (0.00%)
Alanine aminotransferase increased	1 (1.69%)	1 (1.69%)	0 (0.00%)
Alopecia	1 (1.69%)	0 (0.00%)	0 (0.00%)
Creatinine increased	1 (1.69%)	0 (0.00%)	0 (0.00%)
Blood bilirubin increased	1 (1.69%)	0 (0.00%)	0 (0.00%)

Data are *n* (%). TRAE, treatment-related adverse event. AE, adverse event. CNS, central nervous system.

**Fig 3:**
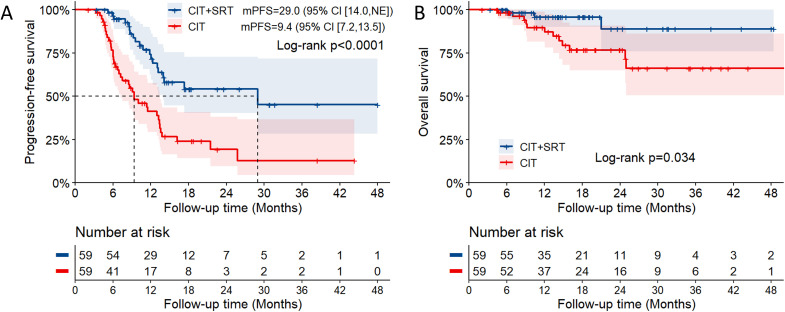
Survival outcomes in patients with (*n* = 59) or without (*n* = 59) consolidative SRT after PSM. Progression-free survival (PFS) **(A)** and overall survival (OS) **(B)** with 95% confidence interval (CI). CIT, chemoimmunotherapy. SRT, stereotactic radiotherapy. NE, not evaluable. mPFS, median PFS.

To establish a contemporary comparison cohort, a total of 1961 patients with metastatic NSCLC receiving first-line chemoimmunotherapy during the period of enrollment were screened (S2 Fig). Among the 260 patients who developed ORD, 136 patients receiving chemoimmunotherapy alone and having adequate follow-up served as the candidates for comparison cohort. After PSM, a comparison cohort with 59 patients was created. The results in Table 3 showed a satisfactory match with no significant differences in clinically relevant covariates between the chemoimmunotherapy + SRT group and the chemoimmunotherapy group. In the chemoimmunotherapy alone group after PSM, the median PFS was 9.4 months (95% CI [7.2,13.5], Fig 3A) and the median OS was not reached (Fig 3B). The addition of consolidative SRT to chemoimmunotherapy was associated with improved PFS (*P* < 0.0001) and OS (*P* = 0.034) with reference to chemoimmunotherapy alone. The incorporation of consolidative cranial SRT was also associated with improved PFS (*P* = 0.0032) among those with residual BMs, although there was no significant difference in OS (*P* = 0.960) (S3 Fig). Moreover, using univariate and multivariate Cox regression analyses, the addition of consolidative SRT to chemoimmunotherapy was identified as an independent factor in better PFS (adjusted HR 0.286, 95% CI [0.164, 0.500]; *P* < 0.001) and OS (adjusted HR 0.229, 95% CI [0.064, 0.819]; *P* = 0.023) with reference to chemoimmunotherapy alone (S3 and S4 Tables) in the propensity score-matched cohort.

**Table 3:** Baseline characteristics of trial cohort and PSM comparison cohort.

Characteristic	CIT + SRT (*n* = 59)	CIT (*n* = 59)	*P* value	SMD
Age group			0.461	0.170
<65 years	23 (38.98%)	26 (44.07%)		
≥65 years	36 (61.02%)	33 (55.93%)		
Sex			0.128	0.341
Female	3 (5.08%)	9 (15.25%)		
Male	56 (94.92%)	50 (84.75%)		
Smoking status			0.526	0.156
Never	13 (22.03%)	17 (28.81%)		
Current or previous	46 (77.97%)	42 (71.19%)		
ECOG PS			0.459	0.171
0	29 (49.15%)	24 (40.68%)		
1	30 (50.85%)	35 (59.32%)		
Histology			0.571	0.139
Non-squamous	34 (57.63%)	38 (64.41%)		
Squamous	25 (42.37%)	21 (35.59%)		
PD-L1 status			0.827	0.175
<1%	23 (38.98%)	23 (38.98%)		
≥1% to <50%	15 (25.42%)	19 (32.20%)		
≥50%	16 (27.12%)	13 (22.03%)		
Unknown	5 (8.47%)	4 (6.78%)		
Stage			0.461	0.170
IVA	33 (55.93%)	28 (47.46%)		
IVB	26 (44.07%)	31 (52.54%)		
Number of involved lesions			0.638	0.175
1	29 (49.15%)	27 (45.36%)		
2–5	21 (35.59%)	19 (32.20%)		
>5	9 (15.25%)	13 (22.03%)		
Number of involved organs			0.387	0.256
1	18 (30.51%)	12 (20.34%)		
2–3	38 (64.41%)	42 (71.19%)		
>3	3 (5.08%)	5 (8.47%)		
Brain metastasis			0.560	0.144
Yes	22 (37.29%)	18 (30.51%)		
No	37 (62.71%)	41 (69.49%)		
Bone metastasis			1.000	<0.001
Yes	22 (37.29%)	22 (37.49%)		
No	37 (62.71%)	37 (62.71%)		
Liver metastasis			1.000	0.072
Yes	3 (5.08%)	4 (6.78%)		
No	56 (94.92%)	55 (93.22%)		

Data are *n* (%) or median (IQR). CIT, chemoimmunotherapy. SRT, stereotactic radiotherapy. SMD, standardized mean difference. ECOG PS, Eastern Cooperative Oncology Group Performance Status.

## Discussion

To the best of our knowledge, the current trial is among the first studies to explore the efficacy and safety of consolidative SRT in first-line chemoimmunotherapy-treated patients with metastatic NSCLC harboring ORD, suggesting a potential clinical benefit of consolidative SRT with manageable toxicities. Additionally, a pre-specified comparison was made with a contemporary cohort of patients who developed ORD but received first-line chemoimmunotherapy alone to further support the clinical value of consolidative SRT, warranting future validation in randomized controlled trials.

The clinical value of consolidative LAT in ICI-treated metastatic NSCLC remains controversial and patient selection may be the key to treatment success. The current trial, along with previous retrospective studies from our group [8,11] and others [21], provided preliminary support for the use of consolidative LAT, especially consolidative SRT, in ICI-treated patients with NSCLC harboring ORD. However, a recent randomized controlled trial (the NRG-LU002) investigating the efficacy and safety of consolidative LAT in majorly ICI-treated metastatic NSCLC reported negative survival results [22]. The major differences between the current trial and the NRG-LU002 study, in our opinion, occurred in the processes of patient selection. First of all, only those who derived prominent clinical benefits from first-line chemoimmunotherapy, manifesting as PR or durable SD, were enrolled in our study. On the other hand, nearly 60% of the patients enrolled in the NRG-LU002 study presented with SD after induction systemic therapy, which was not necessarily durable SD. In contrast, 67.8% of the patients enrolled in the current study had a best response of PR. Previous studies from our study and others found that patients with metastatic NSCLC who developed durable SD, but not short-term SD, generally had comparable prognosis with those who had PR, after ICI treatment [8,23]. In patients who developed PR or durable SD to first-line chemoimmunotherapy, the major components of the tumor lesions were sensitive to ICI treatment, and the drug-resistant subpopulations were effectively eradicated by consolidative SRT, which may extend the duration of ICI treatment and improve patient’s survival. While the SRT cohort’s treatment interval heterogeneity may introduce biological variability, our inclusion of patients with both PR and durable SD reflects real-world clinical scenarios where delayed consolidative therapy remains relevant. Secondly, the status of ORD was confirmed by PET/CT for each patient in our study. The incorporation of PET/CT could help identify patients with truly oligo-metastatic disease by excluding those with occult metastases. Our previous study found that patients with NSCLC with oligo-metastatic disease state confirmed by PET/CT, but not by conventional imaging, could derive clinical benefit from LAT [24], which was also strongly encouraged by several experts’ consensus [14,25]. Meanwhile, a pathological complete response could be obtained after induction chemoimmunotherapy in those with radiographic residual tumor lesions [26,27], for whom consolidative LAT may be unnecessary. Hence, PET/CT should be mandatorily incorporated in patient selection during future trial design and clinical practice in this disease setting.

Besides, the dose-fractionations of radiotherapy may also impact the outcomes of LAT in ICI-treated NSCLC. In the current study, all of the residual lesions, including the primary tumors, were treated with SRT. While, in the NRG-LU002 study, the primary tumor sites and selected metastatic tumor lesions were allowed to be treated with hypo-fractionated radiotherapy (45 Gy in 15 fractions). Previous prospective trials from our group (the SWORD study, NCT04106180) [9] and others [5,28], have shown that SRT may be superior to conventional fractionation and moderate hypofractionation in enhancing anti-tumor immune response when used alone or in combination with ICIs. Mechanically, with higher precise radiation dose, SRT may be more effective in inducing immunogenic cell death and remodeling tumor immune microenvironment, through powerful immune-modulatory pathways, such as cGAS/STING signaling [29], VEGF/VEGFR signaling [30], COX2/IDO1 signaling [31]. Besides, low-dose radiotherapy was also able to reverse tumor immune desertification and resistance to immunotherapy [32,33], and a proof-of-concept study revealed promising efficacy results in patients with metastatic NSCLC treated with triple combination therapy, consisting of low-dose radiotherapy, SRT and PD-1/PD-L1 inhibitors [34]. To validate these findings, the RADIUM trial (NCT06313541) has been initiated as a randomized controlled study evaluating response-adapted hybrid radiotherapy with first-line chemoimmunotherapy in driver mutation-negative metastatic NSCLC. Patients with oligo-residual disease after induction therapy receive SRT using the same dose-fractionation regimens as described in this trial.

Meanwhile, our previous work has hinted potential synergistic effect of cranial radiotherapy with ICIs in metastatic NSCLC and the current trial provided another evidence. Conventionally, the brain is thought to be an immune privilege organ and the immune sensitizing effect of cranial radiotherapy remains largely unknown. However, we found potential synergistic effects between cranial radiotherapy and Atezolizumab treatment in patients with NSCLC with baseline BMs, using individualized patient data from seven large prospective trials [10,35]. In addition, cranial SRT was found to improve patient survival in ICI-treated patients with NSCLC with oligo-BMs in a multicenter real-world study [11]. Moreover, the immune-enhancing effect of cranial radiotherapy was supported by anecdotal case reports of extracranial abscopal response induced by cranial radiotherapy [36] and relevant translational investigations [37]. All of these studies provided preliminary rationale for consolidative cranial SRT in ICI-treated patients with NSCLC with BMs and based on the promising results from the current study, a randomized controlled study (the BRILLIANT study, NCT06501391) has been initiated by our group, to further investigate the safety and efficacy of cranial radiotherapy in first-line chemoimmunotherapy treated patients with NSCLC with BMs.

Beyond consolidative SRT for ORD, salvage SRT for oligo-progressive disease represents another viable strategy combining radiotherapy with chemoimmunotherapy. Recent clinical studies, such as the CURB study [38] and others [39,40], reported encouraging outcomes for this approach. However, we proposed that consolidative SRT for ORD may hold potential advantages over salvage SRT for oligo-progressive disease. Firstly, treatment at minimal tumor burden, achieved after effective systemic therapy when ORD was identified, may enhance SRT efficacy while reducing toxicity. Secondly, not all patients experiencing PD presented with oligo-progressive disease amenable to salvage SRT. Some patients manifested as multi-progressive disease or experienced significant clinical deterioration at initial PD, precluding salvage local therapy. The optimal timing of SRT in chemoimmunotherapy-treated metastatic NSCLC needs to be further investigated.

The toxicities of consolidative SRT in combination with ICIs were generally acceptable, which were generally consistent with former studies. Grade ≥ 2 TRAEs occurred in 25% of the patients receiving hypo-fractionated radiotherapy and ICIs in a pooled analysis of two prospective trials, of which none were radiotherapy-related [41]. A meta-analysis including 51 studies with 15,398 patients, also found comparable grade 3–4 toxicities in those receiving both ICIs and radiotherapy (16.3%; 95% CI [11.1%, 22.3%]) and those receiving ICIs alone (22.3%; 95% CI [18.1%, 26.9%]) [42]. Nevertheless, caution should be taken when combining thoracic SRT with ICIs, since it may increase the risk of pneumonitis [43]. Similarly, ICI treatment may increase the incidence of radiation necrosis [44] and strict domestic constraints should be complied with when combining cranial SRT with ICIs [45].

Our study has several limitations. Firstly, single-arm design makes it challenging to directly determine the superiority of chemoimmunotherapy + consolidative SRT to chemoimmunotherapy alone. To address this issue, a pre-specified comparison with a contemporary cohort of patients receiving first-line chemoimmunotherapy alone was conducted and a 1:1 PSM analysis considering as many factors as possible was used to further minimize the potential selection bias. However, methodological constraints persist due to univariate/multivariate modeling within a shared dataset and a limited sample size. Nevertheless, some covariates that might be associated with the outcomes were not available, such as the status of tumor mutation burden and tumor-infiltrating lymphocytes [46]. Secondly, due to a lack of prospective data on the treatment efficacies of patients with ORD after first-line chemoimmunotherapy at the time of study design, the median PFS of 10.0 months from a pooled analysis of individual patient data was used as the historical control based on our previous work [8,11]. The PFS of the comparison cohort deriving from a prospectively maintained database with a relatively large sample size, as well as the NRG-LU002 study, was generally consistent with the historical control, highlighting the robustness of our previous estimation, which could provide an important reference for future randomized controlled trials. Thirdly, the generalizability of these findings is constrained by the underrepresentation of female patients, a reflection of both the high driver mutation rates in Chinese female NSCLC populations [47] and potential sex-specific differences in immunotherapy efficacy [48]. Lastly, the observed associations between consolidative SRT and OS outcomes should be interpreted cautiously since the OS data was extremely immature due to limited time of follow-up in this highly selected patient population with favorable prognosis. Besides, the observational PSM cohort lacked PET/CT confirmation of ORD, potentially influencing lesion detection accuracy and comparability between cohorts. Given the exploratory nature of these secondary endpoints and the absence of multiplicity adjustments, these results are hypothesis-generating and warrant validation in independent cohorts. Future randomized controlled trials are warranted in investigating the efficacy and safety of consolidative SRT in this disease population.

In conclusion, consolidative SRT is associated with prolonged PFS in first-line chemoimmunotherapy-treated patients with metastatic NSCLC harboring ORD, with manageable toxicities. These exploratory findings warrant validation in randomized controlled trials.

## Supporting information

S1 Fig Survival outcomes in patients receiving cranial consolidative SRT for residual BMs (*n* = 22). Progression-free survival (PFS) **(A)** and overall survival (OS) **(B)** with 95% confidence interval (CI). NE, not evaluable. mPFS, median PFS.
(TIF)

S2 Fig Enrollment flowchart of contemporary comparison cohort. NSCLC, non-small cell lung cancer. CIT, chemoimmunotherapy. PD, progressive disease. SD, stable disease. PR, partial response. ORD, oligo-residual disease. PSM, propensity score-matched.
(TIF)

S3 Fig Survival outcomes in the patients with (*n* = 22) or without (*n* = 22) cranial consolidative SRT for residual BMs after PSM. Progression-free survival (PFS) **(A)** and overall survival (OS) **(B)** with 95% confidence interval (CI). NE, not evaluable. mPFS, median PFS.
(TIF)

S1 Table SRT sites number and organs. SRT, stereotactic radiotherapy.
(DOCX)

S2 Table Subsequent treatment in patients developed progressive disease. CIT, chemoimmunotherapy. SRT, stereotactic radiotherapy.
(DOCX)

S3 Table Univariate and multivariate Cox regression analyses of PFS. HR, hazard ratio. CI, confidence interval. ECOG PS, Eastern Cooperative Oncology Group Performance Status. SRT, stereotactic radiotherapy.
(DOCX)

S4 Table Univariate and multivariate Cox regression analyses of OS. HR, hazard ratio. CI, confidence interval. ECOG PS, Eastern Cooperative Oncology Group Performance Status. SRT, stereotactic radiotherapy.
(DOCX)

S1 Protocol The protocol of current trial. (DOCX)

S2 Protocol The protocol of the mentioned prospective observational study. (DOCX)

S3 Protocol Protocol in original language. (DOCX)

S1 CONSORT Checklist The checklist per the CONSORT (Consolidated Standards of Reporting Trials) 2025 guideline. (DOCX)

## Abbreviations

**:**
ICIs: immune checkpoint inhibitors
LAT: local ablative therapy
NSCLC: non-small cell lung cancer
ORD: oligo-residual disease
PFS: progression-free survival
SRT: stereotactic radiotherapy
TRAEs: treatment-related adverse events
