# Supplementary figures and images for: Consolidative stereotactic radiotherapy for oligo-residual non-small cell lung cancer after first-line chemoimmunotherapy: A single-arm, phase 2 trial from China
Source: PLoS Med. 2025 Aug 1;22(8):e1004680. doi: 10.1371/journal.pmed.1004680 (PMC12316271; doi:10.1371/journal.pmed.1004680)

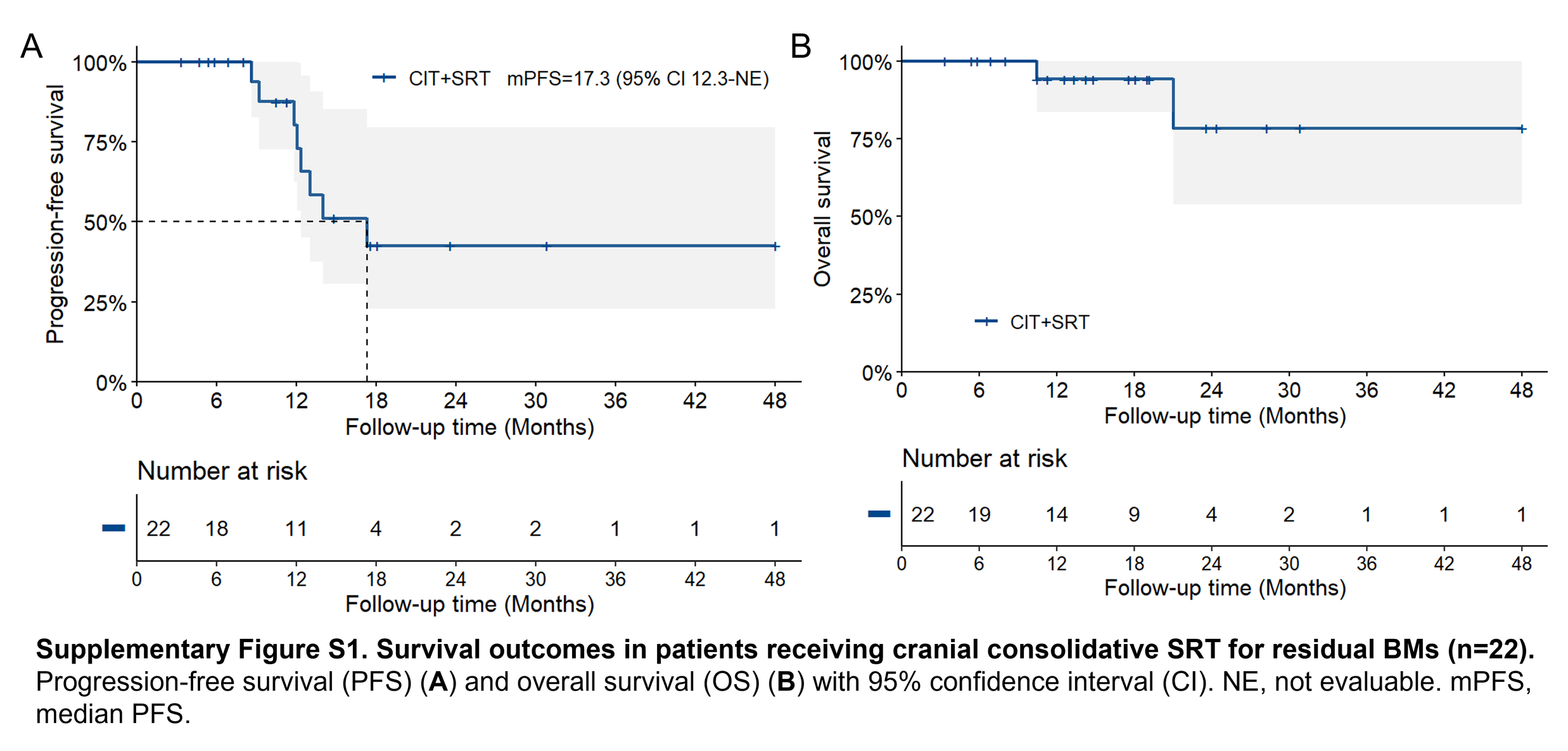

Supplement: S1 Fig — Progression-free survival (PFS) (A) and overall survival (OS) (B) with 95% confidence interval (CI). NE, not evaluable. mPFS, median PFS. (TIF) [file pmed.1004680.s001.tif]

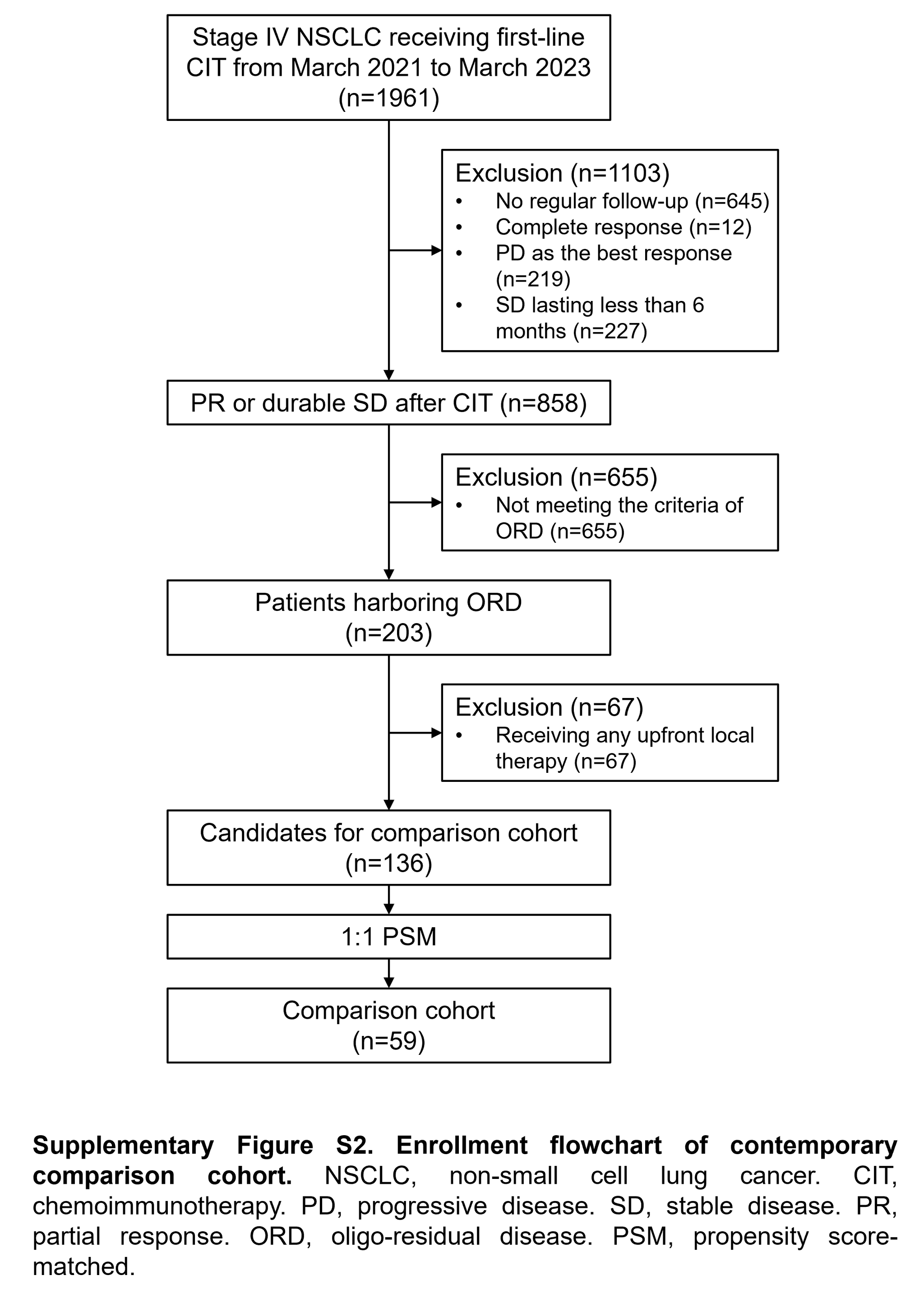

Supplement: S2 Fig — NSCLC, non-small cell lung cancer. CIT, chemoimmunotherapy. PD, progressive disease. SD, stable disease. PR, partial response. ORD, oligo-residual disease. PSM, propensity score-matched. (TIF) [file pmed.1004680.s002.tif]

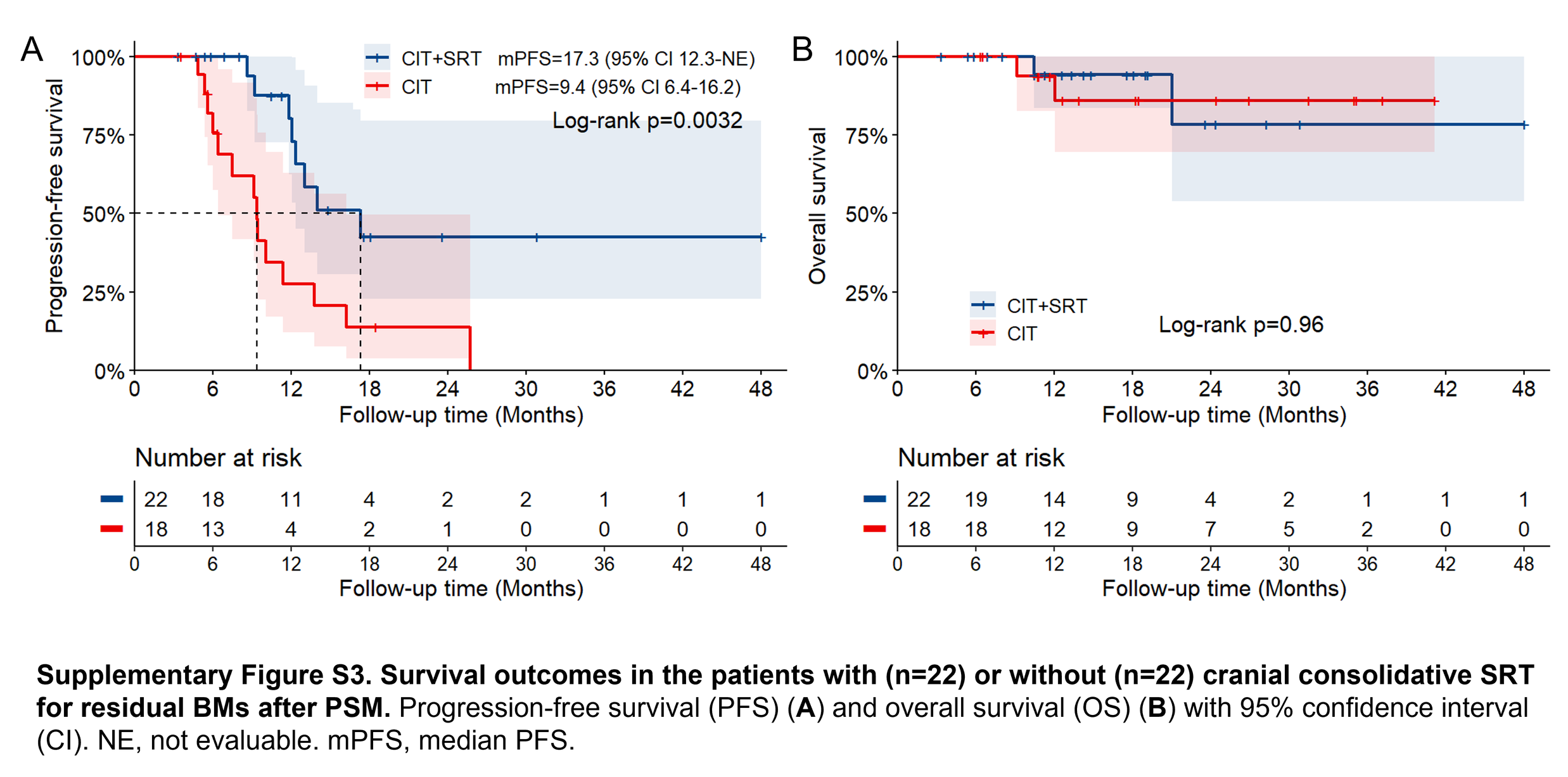

Supplement: S3 Fig — Progression-free survival (PFS) (A) and overall survival (OS) (B) with 95% confidence interval (CI). NE, not evaluable. mPFS, median PFS. (TIF) [file pmed.1004680.s003.tif]
