# Supplementary material for: Consolidative stereotactic radiotherapy for oligo-residual non-small cell lung cancer after first-line chemoimmunotherapy: A single-arm, phase 2 trial from China
Source: PLoS Med. 2025 Aug 1;22(8):e1004680. doi: 10.1371/journal.pmed.1004680 (PMC12316271; doi:10.1371/journal.pmed.1004680)
Supplement: S1 Table — SRT, stereotactic radiotherapy. (DOCX) [file pmed.1004680.s004.docx]

Table S1. SRT sites number, organs, and dose fractionation.

| SRT sites | | Number of patients (n=59) |
| --- | --- | --- |
| Number of SRT sites | |  |
|  | 1 | 25 (42.37%) |
|  | 2 | 20 (33.90%) |
|  | 3-5 | 14 (23.73%) |
| SRT sites and dose fractionation | |  |
|  | Peripheral lung (45 Gy in 3 fractions) | 18 (30.51%) |
|  | Central lung (50 Gy in 5 fractions) | 12 (20.34%) |
|  | Mediastinal/cervical lymph nodes (50 Gy in 5 fractions) | 26 (44.07%) |
|  | Bone/osseous (30 Gy in 3 fractions) | 22 (37.29%) |
|  | Brain (27 Gy in 3 fractions) | 22 (37.29%) |
|  | Liver (45 Gy in 3 fractions) | 3 (5.08%) |
|  | Abdominal-pelvic (45 Gy in 3 fracitons) | 6 (10.17%) |

SRT, stereotactic radiotherapy.
