# Supplementary material for: Consolidative stereotactic radiotherapy for oligo-residual non-small cell lung cancer after first-line chemoimmunotherapy: A single-arm, phase 2 trial from China
Source: PLoS Med. 2025 Aug 1;22(8):e1004680. doi: 10.1371/journal.pmed.1004680 (PMC12316271; doi:10.1371/journal.pmed.1004680)
Supplement: S2 Table — CIT, chemoimmunotherapy. SRT, stereotactic radiotherapy. (DOCX) [file pmed.1004680.s005.docx]

Table S2. Subsequent treatment in patients developed progressive disease.

| Treatment | CIT+SRT | CIT |
| --- | --- | --- |
| PD-1/PD-L1 inhibitors + chemotherapy | 3 | 9 |
| Chemotherapy ± anti-angiogenic agents | 9 | 15 |
| Radiotherapy/surgery | 7 | 5 |
| Unknown | 2 | 6 |

CIT, chemoimmunotherapy. SRT, stereotactic radiotherapy.
