# Supplementary material for: Consolidative stereotactic radiotherapy for oligo-residual non-small cell lung cancer after first-line chemoimmunotherapy: A single-arm, phase 2 trial from China
Source: PLoS Med. 2025 Aug 1;22(8):e1004680. doi: 10.1371/journal.pmed.1004680 (PMC12316271; doi:10.1371/journal.pmed.1004680)
Supplement: S3 Table — HR, hazard ratio. CI, confidence interval. ECOG PS, Eastern Cooperative Oncology Group Performance Status. SRT, stereotactic radiotherapy. (DOCX) [file pmed.1004680.s006.docx]

Table S3. Univariate and multivariate Cox regression analyses of PFS

| Variates | | Univariate analysis | |  | Multivariate analysis | |
| --- | --- | --- | --- | --- | --- | --- |
|  |  | HR (95% CI) | *P* value |  | HR (95% CI) | *P* value |
| Age | | 0.995 [0.964,1.027] | 0.751 |  |  |  |
| Sex | |  |  |  |  |  |
|  | Female | 1 | **0.038** |  | 1 | 0.611 |
|  | Male | 0.467 [0.227,0.960] |  |  | 0.811 [0.363,1.815] |  |
| Smoking Status | |  |  |  |  |  |
|  | Never | 1 | 0.465 |  |  |  |
|  | Current or previous | 0.810 [0.461,1.425] |  |  |  |  |
| ECOG PS | |  |  |  |  |  |
|  | 0 | 1 | 0.461 |  |  |  |
|  | 1 | 1.215 [0.724,2.038] |  |  |  |  |
| Histology | |  |  |  |  |  |
|  | Non,squamous | 1 | 0.890 |  |  |  |
|  | Squamous | 0.964 [0.571,1.627] |  |  |  |  |
| PD-L1 status | |  |  |  |  |  |
|  | <1% | 1 | **0.001** |  | 1 | **<0.001** |
|  | ≥1% to <50% | 0.437 [0.225,0.847] |  |  | 0.350 [0.179,0.684] |  |
|  | ≥50% | 0.394 [0.193,0.806] |  |  | 0.337 [0.164,0.691] |  |
|  | Unknown | 1.691 [0.783,3.654] |  |  | 1.693 [0.722,3.970] |  |
| Stage | |  |  |  |  |  |
|  | IVA | 1 | 0.938 |  |  |  |
|  | IVB | 1.021 [0.612,1.703] |  |  |  |  |
| Number of involved lesions | |  |  |  |  |  |
|  | 1 | 1 | 0.880 |  |  |  |
|  | 2-5 | 1.137 [0.636,2.032] |  |  |  |  |
|  | >5 | 1.166 [0.543,2.503] |  |  |  |  |
| Number of involved organs | |  |  |  |  |  |
|  | 1 | 1 | 0.762 |  |  |  |
|  | 2-3 | 0.841 [0.468,1.512] |  |  |  |  |
|  | >3 | 1.096 [0.400,3.001] |  |  |  |  |
| Brain metastasis | |  |  |  |  |  |
|  | No | 1 | 0.544 |  |  |  |
|  | Yes | 1.178 [0.695,1.998] |  |  |  |  |
| Bone metastasis | |  |  |  |  |  |
|  | No | 1 | 0.804 |  |  |  |
|  | Yes | 0.934 [0.544,1.603] |  |  |  |  |
| Liver metastasis | |  |  |  |  |  |
|  | No | 1 | 0.470 |  |  |  |
|  | Yes | 1.403 [0.560,3.513] |  |  |  |  |
| SRT | |  |  |  |  |  |
|  | Without SRT | 1 | **<0.001** |  | 1 | **<0.001** |
|  | With SRT | 0.341 [0.198,0.586] |  |  | 0.286 [0.164,0.500] |  |

HR, hazard ratio. CI, confidence interval. ECOG PS, Eastern Cooperative Oncology Group Performance Status. SRT, stereotactic radiotherapy.
