# Supplementary material for: Consolidative stereotactic radiotherapy for oligo-residual non-small cell lung cancer after first-line chemoimmunotherapy: A single-arm, phase 2 trial from China
Source: PLoS Med. 2025 Aug 1;22(8):e1004680. doi: 10.1371/journal.pmed.1004680 (PMC12316271; doi:10.1371/journal.pmed.1004680)
Supplement: S4 Table — HR, hazard ratio. CI, confidence interval. ECOG PS, Eastern Cooperative Oncology Group Performance Status. SRT, stereotactic radiotherapy. (DOCX) [file pmed.1004680.s007.docx]

Table S4. Univariate and multivariate Cox regression analyses of OS

| Variates | | Univariate analysis | |  | Multivariate analysis | |
| --- | --- | --- | --- | --- | --- | --- |
|  |  | HR (95% CI) | *P* value |  | HR (95% CI) | *P* value |
| Age | | 1.053 [0.986,1.124] | 0.122 |  |  |  |
| Sex | |  |  |  |  |  |
|  | Female | 1 | 0.330 |  |  |  |
|  | Male | 0.533 [0.150,1.889] |  |  |  |  |
| Smoking Status | |  |  |  |  |  |
|  | Never | 1 | 0.474 |  |  |  |
|  | Current or previous | 0.675 [0.230,1.978] |  |  |  |  |
| ECOG PS | |  |  |  |  |  |
|  | 0 | 1 | 0.461 |  |  |  |
|  | 1 | 1.215 [0.724,2.038] |  |  |  |  |
| Histology | |  |  |  |  |  |
|  | Non,squamous | 1 | **0.050** |  | 1 | **0.020** |
|  | Squamous | 0.356 [0.126,1.001] |  |  | 0.289 [0.102,0.821] |  |
| PD,L1 status | |  |  |  |  |  |
|  | <1% | 1 | 0.437 |  |  |  |
|  | ≥1% to <50% | 0.530 [0.126,2.225] |  |  |  |  |
|  | ≥50% | 0.997 [0.287,3.462] |  |  |  |  |
|  | Unknown | 2.453 [0.465,12.948] |  |  |  |  |
| Stage | |  |  |  |  |  |
|  | IVA | 1 | 0.938 |  |  |  |
|  | IVB | 1.021 [0.612,1.703] |  |  |  |  |
| Number of involved lesions | |  |  |  |  |  |
|  | 1 | 1 | 0.102 |  |  |  |
|  | 2-5 | 1.465 [0.152,14.121] |  |  |  |  |
|  | >5 | 4.583 [0.590,35.622] |  |  |  |  |
| Number of involved organs | |  |  |  |  |  |
|  | 1 | 1 | 0.762 |  |  |  |
|  | 2-3 | 0.841 [0.468,1.512] |  |  |  |  |
|  | >3 | 1.096 [0.400,3.001] |  |  |  |  |
| Brain metastasis | |  |  |  |  |  |
|  | No | 1 | 0.435 |  |  |  |
|  | Yes | 1.256 [0.708,2.227] |  |  |  |  |
| Bone metastasis | |  |  |  |  |  |
|  | No | 1 | 0.170 |  |  |  |
|  | Yes | 2.428 [0.684,8.611] |  |  |  |  |
| Liver metastasis | |  |  |  |  |  |
|  | No | 1 | 0.538 |  |  |  |
|  | Yes | 21.993 [0.001,NA] |  |  |  |  |
| SRT | |  |  |  |  |  |
|  | Without SRT | 1 | **0.048** |  | 1 | **0.023** |
|  | With SRT | 0.278 [0.078,0.988] |  |  | 0.229 [0.064,0.819] |  |

HR, hazard ratio. CI, confidence interval. ECOG PS, Eastern Cooperative Oncology Group Performance Status. SRT, stereotactic radiotherapy.
