# Supplementary material for: Consolidative stereotactic radiotherapy for oligo-residual non-small cell lung cancer after first-line chemoimmunotherapy: A single-arm, phase 2 trial from China
Source: PLoS Med. 2025 Aug 1;22(8):e1004680. doi: 10.1371/journal.pmed.1004680 (PMC12316271; doi:10.1371/journal.pmed.1004680)
Supplement: S1 Protocol — (DOCX) [file pmed.1004680.s008.docx]

**Consolidative stereotactic radiotherapy in oligo-residual non-small cell lung cancer after first-line chemoimmunotherapy**

**Responsible institution:** Fudan University Shanghai Cancer Center

Principal investigator: Zhengfei Zhu

**Protocol number: 2020-NSCLCSBRT**

**Protocol version number: 1.0**

**Scheme version date: 2021.1.20**

**Protocol signature page**

**Clinical Trial Unit Statement**

I will record the clinical trial data in accordance with the requirements of this protocol (version number: 1.0, version date: 2021.01.20), and ensure that the clinical trial data are true, accurate, timely, and lawful to be included in the case report form. I will be responsible for making medical decisions related to the clinical trial to ensure that if adverse events occur during the trial, the subject will receive treatment as promptly as possible. I am aware of the procedures and requirements for proper reporting of serious adverse events, and if an adverse event or serious adverse event is identified in the context of a clinical study, I will report it as required in the protocol.

Fudan University Shanghai Cancer Center

Principal Investigator (signature) :

Date (YYYY/MM/DD):

**1.0 Research Title**

Consolidative stereotactic radiotherapy in oligo-residual non-small cell lung cancer after first-line chemoimmunotherapy

**2.0 Background**

Lung cancer is a malignant tumor that seriously endangers human health. There were about 1.8 million new cases of lung cancer in the world in 2012, accounting for 12.9% of all new cancer cases and 19.4% of cancer-attributable deaths [1, 2]. Non-small cell lung cancer (NSCLC) accounts for 85% of all lung cancers, and nearly 50% of patients are diagnosed at an advanced stage, with limited treatment options and poor prognosis [3, 4].

PD-1/PD-L1 inhibitors are a major breakthrough in the treatment of advanced NSCLC. PD-1/PD-L1 inhibitors alone or in combination with chemotherapy have become the standard first-line treatment for advanced NSCLC without EGFR or ALK mutations. The emergence of PD-1/PD-L1 inhibitors has increased the 5-year overall survival rate of advanced NSCLC without EGFR or ALK mutations from about 5% to about 20% [5-11]. Up to now, Pembrolizumab, alone or in combination with chemotherapy has been approved by the Chinese Food and Drug Administration for the first-line treatment of advanced NSCLC without EGFR or ALK mutation. Nivolumab, another PD-1 inhibitor, has been approved as a second-line treatment for advanced NSCLC without EGFR or ALK mutation.

As more and more patients with advanced NSCLC receive PD-1/PD-L1 inhibitors, acquired resistance has gradually become a problem. Accumulating evidence suggest that 30%-40% of advanced NSCLC patients who have received PD-1/PD-L1 inhibitor treatment and achieved objective response will eventually develop acquired resistance [12, 13]. In June 2018, Scott N Gettinger et al. from Yale Cancer Center first reported the clinical phenotype of acquired resistance in advanced NSCLC treated with PD-1/PD-L1 inhibitors. In that study, 26 cases of metastatic NSCLC with acquired resistance to PD-1/PD-L1 inhibitors were included, of whom 23 (88.0%) developed oligo-progressive disease. The majority of progressive disease was limited to one (54%) or two (35%) sites, and mainly developed in the lymph nodes (including 11 patients with disease progression occurred only in the lymph nodes) [14].

The oligometastatic state is a transition stage between focal disease and extensive metastasis. In this stage, the number of tumor lesions is limited and the speed of progression is mild [[1](#_ENREF_1)]. Correspondingly, an oligo-residual state refers to the situation in which the residual lesions are distributed in a few organs (≤3) and a few lesions (≤5) after effective systemic therapy[[2-5](#_ENREF_2)]. For these patients with oligo-residual disease, local treatments such as radiotherapy, surgery, and radiofrequency ablation, can significantly prolong the progression-free survival (PFS) [[3-5](#_ENREF_3)]. In the era of targeted therapy, consolidative local therapy was found to improve PFS in the prospective phase 2 ATOM study [[2](#_ENREF_2)]. However, in the era of immunotherapy, resistance to PD-1/PD-L1 inhibitors remains an obstacle to achieving durable disease control.

The optimal timing of local therapy in patients with advanced NSCLC treated with PD-1/PD-L1 inhibitors is still controversial. Scott N Gettinger, et al. at the Yale Cancer Center analyzed 26 patients who developed acquired resistance to PD-1/PD-L1 inhibitors and among them, 15 patients received salvage local therapy, the majority of which was salvage radiotherapy. Of the 15 patients receiving salvage local therapy, PD-1/PD-L1 inhibitors were continued in 11 patients, which resulted in no grade 3-4 adverse events. The 2-year survival rate of these 15 patients reached 92%, which was significantly higher than that of other patients who did not receive salvage local treatment (44%) [[6](#_ENREF_6)]. However, there are few reports on the use of local therapy to manage oligo-residual disease after PD-1/PD-L1 inhibitor therapy.

Stereotactic radiotherapy (SRT), based on the modern radiotherapy technology platform, delivers radiotherapy with higher dose fractions within fewer fractions. It has higher conformity to the tumor and less damage to the surrounding tissues, making it a safe and efficient local treatment method. The application of SRT in the treatment of oligometastatic advanced NSCLC with targeted therapy has been widely reported and has gradually become a standard treatment [[7](#_ENREF_7), [8](#_ENREF_8)]. In addition, in the process of SRT, the local radiation dose of the tumor is higher, and more tumor-associated antigens will be released from the irradiated tumor cells. At the same time, SRT can more effectively induce an anti-tumor immune response due to its better protection of the lymphoid tissue around the tumor. In 2016, Joe Y Chang proposed the concept of iSABR, advocating the combination of SRT and immune checkpoint inhibitor for the treatment of solid tumor at all stages [[9](#_ENREF_9)]Since then, the results of a number of retrospective and prospective studies of PD-1/PD-L1 inhibitor combined with SRT for advanced NSCLC have been published. This combination therapy is found to be safe and has shown preliminary efficacy [[10](#_ENREF_10), [11](#_ENREF_11)]. A meta-analysis of 18 representative clinical studies of an immune checkpoint inhibitor (CTLA-4 inhibitor, PD-1 inhibitor) combined with SRT for advanced NSCLC showed that the local control rate was 71%, the response rate in the non-irradiation site was 41%, and the median overall survival time was 12.4 months [[10](#_ENREF_10)]. The Pembro-RT trial, which reported preliminary results in June 2018, compared SRT plus Pembrolizumab with Pembrolizumab monotherapy in previously treated advanced NSCLC, and the results showed that the addition of SRT can improve the response rate (41% vs. 19%) and prolong PFS (6.4 vs. 1.8 months) [[12](#_ENREF_12)]. These studies suggest that SRT is a safe and effective local treatment method, and has the potential to increase the sensitivity of PD-1/PD-L1 inhibitors in metastatic NSCLC.

**References**

1. Weichselbaum, R.R. and S. Hellman, *Oligometastases revisited.* Nat Rev Clin Oncol, 2011. **8**(6): p. 378-82.

2. Chan, O.S.H., et al., *ATOM: A phase II study to assess efficacy of preemptive local ablative therapy to residual oligometastases of NSCLC after EGFR TKI.* Lung Cancer, 2020. **142**: p. 41-46.

3. Guo, T., et al., *Pattern of Recurrence Analysis in Metastatic EGFR-Mutant NSCLC Treated with Osimertinib: Implications for Consolidative Stereotactic Body Radiation Therapy.* Int J Radiat Oncol Biol Phys, 2020. **107**(1): p. 62-71.

4. Miyawaki, T., et al., *Association between oligo-residual disease and patterns of failure during EGFR-TKI treatment in EGFR-mutated non-small cell lung cancer: a retrospective study.* BMC Cancer, 2021. **21**(1): p. 1247.

5. Zeng, Y., et al., *The value of local consolidative therapy in Osimertinib-treated non-small cell lung cancer with oligo-residual disease.* Radiat Oncol, 2020. **15**(1): p. 207.

6. Gettinger, S.N., et al., *Clinical Features and Management of Acquired Resistance to PD-1 Axis Inhibitors in 26 Patients With Advanced Non-Small Cell Lung Cancer.* J Thorac Oncol, 2018. **13**(6): p. 831-839.

7. Ning, M.S., et al., *Stereotactic ablative body radiation for oligometastatic and oligoprogressive disease.* Transl Lung Cancer Res, 2019. **8**(1): p. 97-106.

8. Basler, L., S.G. Kroeze, and M. Guckenberger, *SBRT for oligoprogressive oncogene addicted NSCLC.* Lung Cancer, 2017. **106**: p. 50-57.

9. Bernstein, M.B., et al., *Immunotherapy and stereotactic ablative radiotherapy (ISABR): a curative approach?* Nat Rev Clin Oncol, 2016. **13**(8): p. 516-24.

10. Chicas-Sett, R., et al., *Stereotactic Ablative Radiotherapy Combined with Immune Checkpoint Inhibitors Reboots the Immune Response Assisted by Immunotherapy in Metastatic Lung Cancer: A Systematic Review.* Int J Mol Sci, 2019. **20**(9).

11. Luke, J.J., et al., *Safety and Clinical Activity of Pembrolizumab and Multisite Stereotactic Body Radiotherapy in Patients With Advanced Solid Tumors.* J Clin Oncol, 2018. **36**(16): p. 1611-1618.

12. Willemijn Theelen, N.F.H.P.N.N., *Randomized phase II study of pembrolizumab after stereotactic body radiotherapy (SBRT) versus pembrolizumab alone in patients with advanced non-small cell lung cancer: The PEMBRO-RT study.* Journal of Clinical Oncology, 2018. **DOI: 10.1200/JCO.2018.36.15_suppl.9023 Journal of Clinical Oncology 36, no. 15_suppl (May 20, 2018) 9023-9023**.

**3.0 Study Objectives**

To explore the safety, efficacy and potential predictors of efficacy of stereotactic radiotherapy (SRT) in patients with advanced non-small cell lung cancer (NSCLC) with oligo-residual disease after effective first-line chemoimmunotherapy.

**4.0 Eligibility Criteria**

**4.1 Inclusion Criteria**

- Age ≥ 18 and ≤ 75 years.
- ECOG PS 0-1.
- Patients with pathologically confirmed stage IV NSCLC by tumor biopsy and/or fine-needle aspiration.
- Negative for driver genes including EGFR, ALK, and ROS-1.
- At least one measurable disease per RECIST1.1.
- Patients should harbor cranial- and/or extracranial ORD after effective first-line chemoimmunotherapy, defined as having partial response (PR) or durable stable disease (SD) (SD lasting no less than 6 months). Extracranial ORD was defined as residual tumors limited to three organs and five lesions among those without baseline BMs or those with complete cranial response. Cranial ORD was defined as the BMs limited to 10 lesions with the largest tumor <10 mL in volume and <3 cm in longest diameter and total cumulative volume ≤15 mL, among those with residual BMs and without extracranial progressive disease (in this circumstance, extracranial ORD was not necessarily required). At least one BM lesion with a diameter >1 cm or a lesion with a diameter >0.5 cm on a 1.5 mm thick thin layer magnetic resonance imaging (MRI) was required for response evaluation.
- The oligo-residual tumor lesions should be amenable to consolidative SRT in the opinion of the investigators.
- Patients with a history of radiotherapy are eligible if they satisfy the following criteria:
  1. Radiotherapy administered more than 4 weeks before study entry.
  2. At least one measurable lesion outside the radiation field.
- Patients with no indications for palliative radiotherapy in the opinion of the investigator.
- Patients with a prior history of surgery are eligible if they have sufficiently recovered from the toxicity and/or complications of surgery.
- Signed informed consent for the use of fresh tumor biopsies before and during the treatment.
- Women of childbearing age and men must agree to use effective contraception during the trial.
- Life expectancy of more than 3 months.
- Adequate organ function within 1 week prior to enrollment:
  1. Adequate bone marrow function: hemoglobin ≥80g/L, white blood cell (WBC) count ≥ 4.0 * 10 ^ 9/L or neutrophil count ≥ 1.5 * 10 ^ 9/L, and platelet count ≥ 100 * 10 ^ 9/L;
  2. Adequate hepatic function: total bilirubin < 1.5 x upper limit of normal (ULN). Note: If total bilirubin is > 1.5 x ULN, direct bilirubin must ≤ ULN, Aspartate aminotransferase (AST) and Alanine aminotransferase (ALT) ≤2.5 ULN;
  3. Adequate renal function: serum creatinine ≤ 1.5 x ULN or creatinine clearance ≥ 50 mL/min;
- Ability to understand and willingness to provide the informed consent.

**4.2 Exclusion Criteria**

- Severe autoimmune disease, such as inflammatory bowel disease (including Crohn's disease and ulcerative colitis), rheumatoid arthritis, scleroderma, systemic lupus erythematosus, Wegener's granulomatosis and related vasculitis.
- Symptomatic interstitial lung disease or clinically active infectious/non-infectious pneumonitis.
- History of another malignancy or concurrent malignancy.
- Active infection, congestive heart failure, or any evidence of myocardial infarction, unstable angina pectoris or cardiac arrhythmia within 6 months prior to enrollment.
- Any evidence of severe or uncontrolled systemic diseases, which in the investigator's opinion makes it undesirable for the patient to participate in the trial or which would jeopardize compliance with the protocol. Screening for chronic conditions is not required.
- Patients in whom palliative radiotherapy is indicated in the opinion of the investigator.
- Mixed small cell with non-small cell lung cancer histology.
- The patient is pregnant (confirmed by serum b-HCG if applicable) or is breastfeeding.
- Patients who have received tumor vaccine; or administration of live, attenuated vaccine within 4 weeks before the start of treatment. Note: Influenza vaccination is permitted only during influenza season, while live, attenuated influenza vaccine such as FluMist is not allowed.
- Patients receiving immunosuppressive agents, or other investigational treatment. Long-term corticosteroid users are also excluded.
- Mental disorders, drug abuse, and social condition that may negatively impact compliance in the investigator's opinion.
- Prior allergic reaction or contraindications to PD-1/PD-L1 inhibitors.

**4.3 Withdrawal Criteria**

- Wrong enrollees. Those who have not received the study treatment should be withdrawn immediately, and their information will not be included in the study analysis. If a study treatment had already been initiated, the decision to withdraw was made after evaluation of the patient's benefit and risk by the investigator. If an exclusion is required, the patient will be withdrawn from the study after completing the post-treatment follow-up, and the patient will not be included in the efficacy analysis but will be included in the safety analysis.
- Patients who had any of the following conditions during the pre-treatment evaluation phase: new onset systemic disease or deterioration of preexisting systemic disease and met the exclusion criteria; Ask for a group or lost to follow-up or death; Compliance is poor.
- For patients who were considered to be excluded by the investigator, the investigator should report the reason for exclusion to the principal investigator. After consent, patients who had not yet received the study treatment should be withdrawn immediately, and their information would not be included in the study analysis. If a patient had already started the study treatment and withdrew from the study after completing the post-treatment follow-up visit, the patient would not be included in the efficacy analysis but would be included in the safety analysis.
- If the subjects do not wish to continue the trial during the course of the trial, they can withdraw from the trial at any time by asking the investigators.
- If a serious adverse event occurs during the trial, the participant should be stopped from participating in the trial according to the investigator's judgment;
- Deterioration in the trial could threaten lives, other impact trial, and/or in the trial observation condition;
- Loss to follow-up or death during the treatment phase;
- Share the trial during chemotherapy, proprietary Chinese medicine, other immune therapy, radiotherapy sensitization agent or other affect curative effect and toxicity of evaluators;
- Serious deviations occurred during the implementation of the clinical trial protocol, which made it difficult to evaluate the efficacy of the drugs. Adherence to claim.

**5.0 Pre-enrollment assessment**

- a complete medical history and demographic data;
- histopathological diagnosis and clinical staging;
- tumor-related symptoms and physical examination;
- driver gene mutations (tissue or peripheral blood testing, ARMS, NGS, or digital PCR);
- baseline tumor assessment (whole-body tumor imaging within 3 weeks before treatment initiation);
- results of routine examination (blood, urine, and stool routine, liver and kidney function, electrocardiogram, etc.).

1. **Treatment and Follow-up**
   1. **Patient treatment**

Patients will receive the original maintainance PD-1/PD-L1 inhibitor treatment for up to 2 years or until confirmed progression or unacceptable toxicity. PD-1/PD-L1 inhibitors will be administrated as an intravenous (IV) infusion. Patients with oligo-residual NSCLC after effective treatment will be treated with curative-intent SRT covering all of the residual lesions. The choice of dose-fractionation regimen is at the discretion of the treating radiation oncologist, referring to the protocol of NRG-BR001 study.

- 1. **Regular follow-up**

1. The first imaging examination is performed after 1 month of SRT treatment and at 2-month intervals thereafter. The specific examination time should be within ±5 days of the scheduled time;
2. The detail of imaging examination is based on the patient's baseline findings before chemoimmunotherapy:
3. Except bone metastasis, the imaging examination of the lesion sites found in the baseline evaluation was required each time.
4. For bone metastases, if there is a definite soft tissue mass, the corresponding imaging examination should be performed, otherwise the corresponding imaging examination is not mandatory. Bone scanning is not mandatory to be performed each time.
5. For sites without lesions at baseline evaluation, additional imaging examination will be performed at the discretion of the responsible physician when the patient has corresponding symptoms or abnormal biochemical indicators.
6. Systemic imaging was considered if a patient had a definite progression of any lesion (according to RECIST1.1 criteria) during the course of treatment.
7. For patients with baseline brain metastasis, brain MRI should be performed at each follow-up and the response of cranial lesions should be evaluated per the RANO criteria.
8. Blood routine, liver and kidney function, tumor markers, and other hematological indicators are examinated as appropriate.
9. The changes in tumor-related symptoms and physical examination results are recorded.
   1. **Examination at the time of disease progression**
10. Physical examination: body weight, body surface area, vital signs;
11. Tumor assessment: tumor-related symptoms, comprehensive imaging examination;
12. Laboratory examination: blood routine, urine routine, liver and kidney function, electrocardiogram (ECG).

**7.0 Sample size**

All patients with driver-mutation negative NSCLC receiving first-line chemoimmunotherapy were included in a prospective observational study, which had been approved and registered as NCT04766515. Those with the best response of partial response (PR) or durable stable disease (SD) (SD lasting no less than 6 months) would be screened for this study. Since there is limited survival data in metastatic NSCLC patients who developed ORD after effective first-line chemoimmunotherapy, we pooled individualized patient data from three previous retrospective studies and found a median PFS of 10.0 month for this disease population, which served as the historical control. Adding consolidative SRT, the risk of disease progression or death would be reduced by 40% (HR=0.6). Under the condition of the Power = 90% and α = 0.05 (one-sided), this single-arm study needs total of 53 evaluable subjects. Considering 10% dropout rate before the data analysis, we need a total of 59 subjects. Moreover, a propensity score-matched (PSM) comparison will be conducted with a contemporary cohort of patients who developed ORD but received systematic therapy alone in order to further investigate the clinical value of consolidative SRT.

1. **Study evaluation**
   1. **Study measuring**

**8.1.1 Primary endpoints**

**Progression-free survival (PFS)**. PFS is measured from the date of the initiation of first-line chemoimmunotherapy to the date of initial disease progression as defined by Response Evaluation Criteria in Solid Tumor (RECIST) Version 1.1 or death. Participants survived without progression at the time of data analysis were censored at the date of last follow-up.

**8.1.2 Secondary endpoints**

**Treatment-related adverse events (TRAEs)**. TRAEs will be assessed and graded according to CTCAE v.5.0.

**Overall survival (OS)**. OS is measured from the date of the initiation of first-line chemoimmunotherapy to the date of death by any cause. Participants survived at the time of data analysis were censored at the date of last follow-up.

**8.1.3 Exploratory endpoints**

This study will obtain peripheral blood and tissue samples at specific time point, and preserve and process in accordance with the specification, for translational research as follows:

1. Correlation between molecular subsets of immune-infiltrating cells in baseline tumor tissue and response to treatment.
2. Correlation between curative effect and cytokines, immune cell subsets and their dynamic change in peripheral blood.
   1. **Response evaluation criteria**

According to response evaluation criteria in solid tumors (RECIST) version 1.1 (see Appendix 3).

**9.0 the annual research plan**

| 2021.1-2021.03 | Start of the clinical research. |
| --- | --- |
| 2021.03-2021.12 | Subject enrollment and treatment with SBRT (6~7 subjects per month averagely). |
| 2022.01-2022.12 | Patient enrollment. Regular follow-up. Continuous dynamic peripheral blood samples collection. |
| 2023.01-2023.12 | Follow-up completion. Data collection and analysis. Peripheral blood samples detection and analysis; Summary of the data and paper writing. |

**10.0 Ethical and legal issues**

**10.1 Independent ethics committee (IEC)**

According to the requirements of GCP, China's laws and regulations and the relevant organizations, all center involved in the study should obtain the ethics committee approval documents before the beginning of study, and amended or retrial if necessary.

**10.2 Ethical guidance of this study**

The solution related to operation, evaluation and program files involved in this study is to ensure that the researchers in detail in the following clinical practice guidelines and the declaration of Helsinki guidelines. The implementation of this study will also follow the relevant laws and regulations in China.

The researchers can't modify research plan without ethics committee and the sponsor's written consent. However, in case of an emergency in order to remove the participants' risk factors, the researchers can deviate or change the plan without the consent or support of the ethics committee/sponsor. The deviation or change and reasons shall be submitted to the ethics committee/sponsor as soon as possible, if appropriate, should also submit a proposal to modify. Researchers must fully explain all the deviation or change of research.

**10.3 Participants information and informed consent**

The main information and informed consent form of the study shall be provided to the subjects. Before the start of the study, researchers must provide participants with informed consent form approved by the ethics committee and all other written information. Ethics committee approval documents and approved subject guidelines/ informed consent form must be archived in the study file.

Before implementation of any specific steps of this study, the signed informed consent of subjects must be gotten.

**10.4 Confidentiality**

About the identity of the patients with all records are confidential, and in accordance with the relevant laws and/or regulations allow, these data will not be made public. Only relevant people, such as researchers, research nurses can know the patient's identity information.

The names of the participants will not show up in the case report form. Case report form only records the subject number and the abbreviation of name, if the subjects' names appear in any other documents (such as pathological report, imaging examinations), it must be covered in the copy of the file. The reports using computer storage must be in accordance with local data protection laws. When the results of the study is published, the identity of the subjects will be confidential.

The researchers will retain a list to identify the subjects' records.

**10.5 Conditions of the research plan revision**

Unless it is an emergency adjustment in order to eliminate the damage of trial subjects, or just about the adjustment of the experimental study on the logistics and management, such as arbitrator, number of replacement. All revised plan must be submitted to the appropriate ethics committee, and obtain the license after researchers to implement adjustments.

**10.6 Surveillance**

If the patient confidentiality conform to the local requirements, responsible arbitrator will regularly contact and visit the researchers, and is allowed to check various trial record form (cases) and other relevant data.

Throughout the study period, responsible arbitrator is responsible for the regular inspection report, verify compliance of the research program, and check the input data integrity, consistency and accuracy. Arbitrator should get access to a laboratory trial report and other privileges, patient records to verify the input on the case report. The researchers (or its designated personnel) agreed cooperates with, to ensure that found during the inspection visit any of the problems can be resolved.

**Appendix 1. Karnofsky performance status scale definitions rating (%) criteria**

**
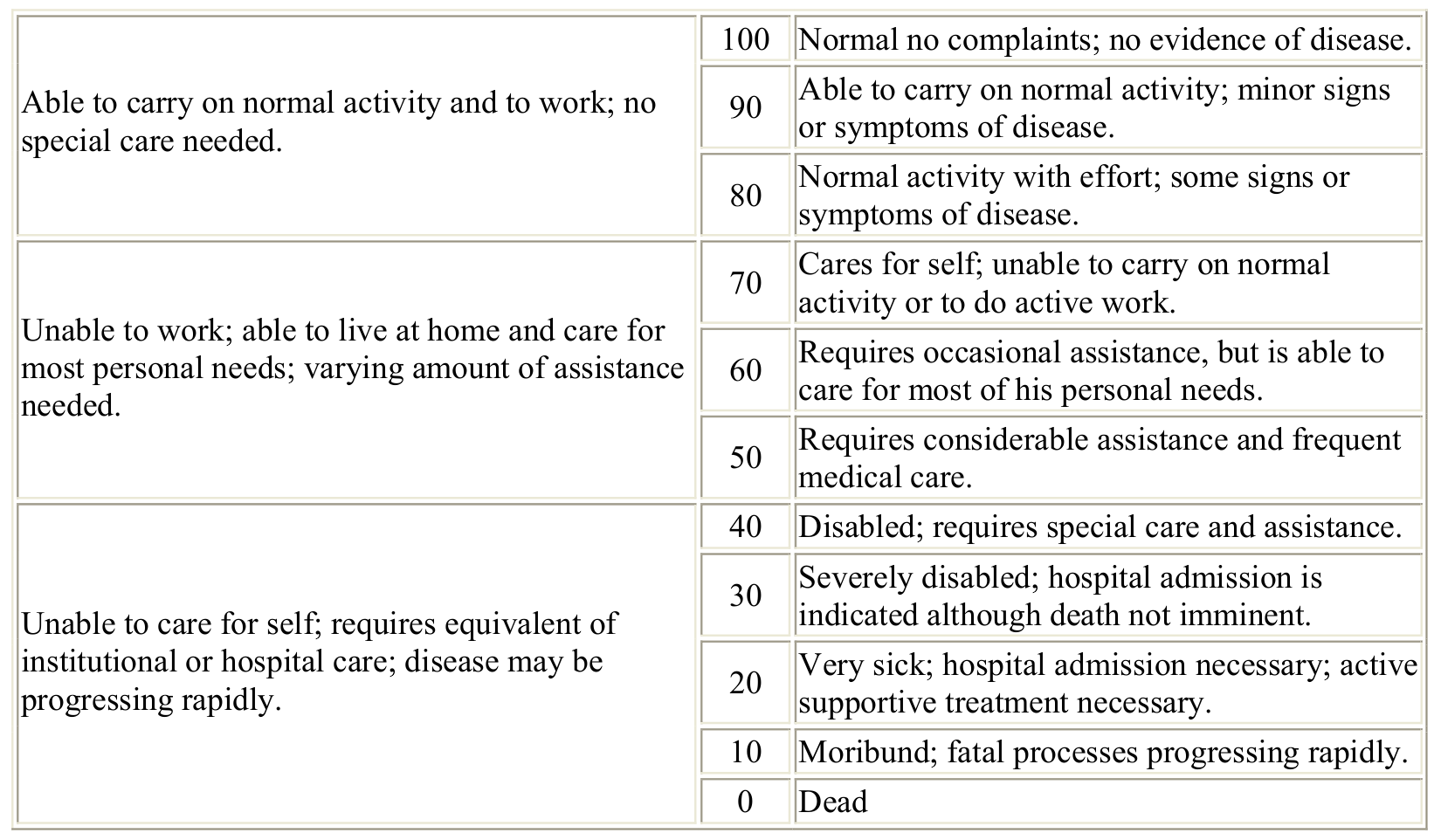
**

**Appendix 2. The Eighth Edition Lung Cancer Stage Classification**


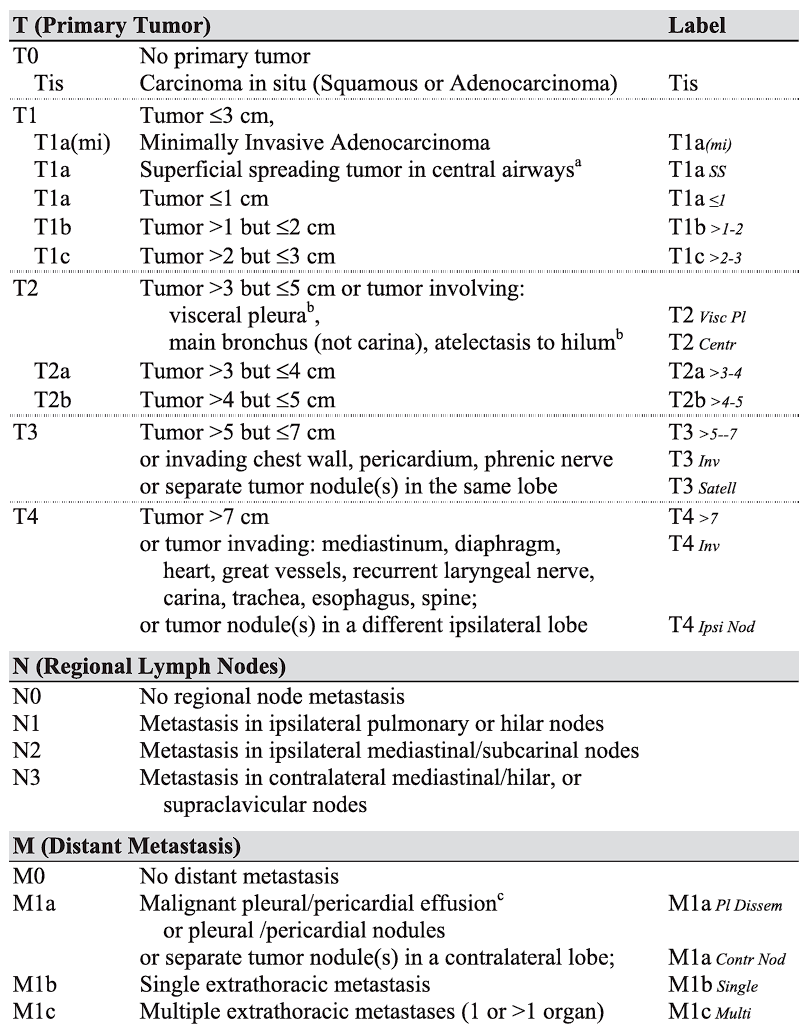


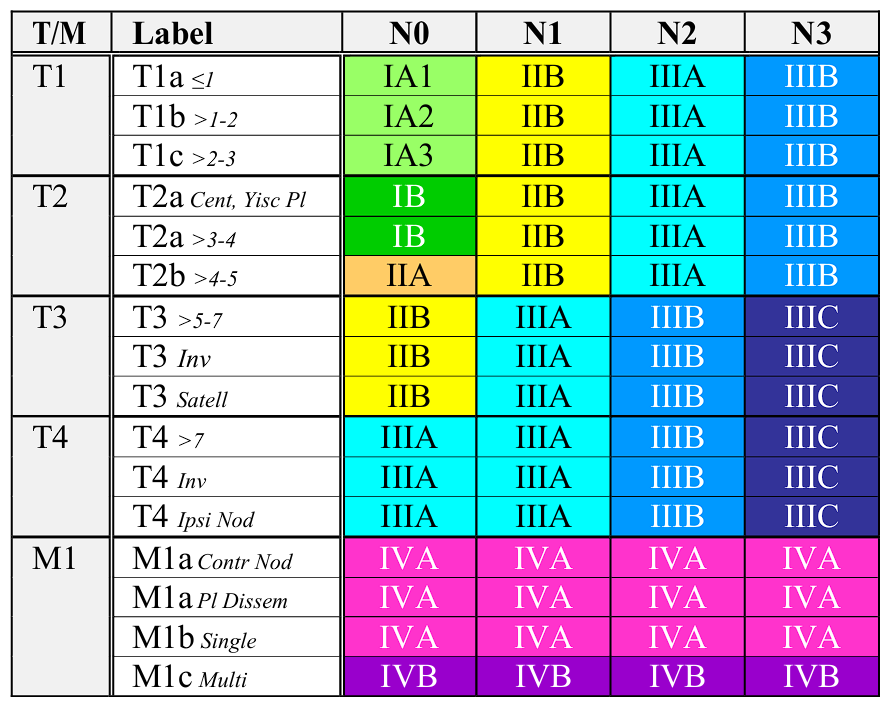


**Appendix 3. New response evaluation criteria in solid tumours: Revised RECIST guideline (version 1.1)**

3. Measurability of tumour at baseline

3.1. Definitions

At baseline, tumour lesions/lymph nodes will be categorised measurable or non-measurable as follows:

3.1.1. Measurable

*Tumour lesions*: Must be accurately measured in at least one dimension (*longest* diameter in the plane of measurement is to be recorded) with a *minimum* size of:

- 10 mm by CT scan (CT scan slice thickness no greater than 5 mm; see [Appendix II](https://www.sciencedirect.com/science/article/pii/S0959804908008733?via%3Dihub#app2) on imaging guidance).
- 10 mm caliper measurement by clinical exam (lesions which cannot be accurately measured with calipers should be recorded as non-measurable).
- 20 mm by chest X-ray.

*Malignant lymph nodes*: To be considered pathologically enlarged *and* measurable, a lymph node must be ⩾15 mm in *short* axis when assessed by CT scan (CT scan slice thickness recommended to be no greater than 5 mm). At baseline and in follow-up, only the *short* axis will be measured and followed (see Schwartz et al. in this Special Issue^[15](https://www.sciencedirect.com/science/article/pii/S0959804908008733?via%3Dihub#bib15)^). See also notes below on ‘Baseline documentation of target and non-target lesions’ for information on lymph node measurement.

3.1.2. Non-measurable

All other lesions, including small lesions (longest diameter <10 mm or pathological lymph nodes with ⩾10 to <15 mm short axis) as well as truly non-measurable lesions. Lesions considered truly non-measurable include: leptomeningeal disease, ascites, pleural or pericardial effusion, inflammatory breast disease, lymphangitic involvement of skin or lung, abdominal masses/abdominal organomegaly identified by physical exam that is not measurable by reproducible imaging techniques.

3.1.3. Special considerations regarding lesion measurability

Bone lesions, cystic lesions, and lesions previously treated with local therapy require particular comment:

**Bone lesions:**

- Bone scan, PET scan or plain films are not considered adequate imaging techniques to measure bone lesions. However, these techniques can be used to confirm the presence or disappearance of bone lesions.
- Lytic bone lesions or mixed lytic-blastic lesions, with *identifiable* *soft* *tissue* *components*, that can be evaluated by cross sectional imaging techniques such as CT or MRI can be considered as measurable lesions if the *soft* *tissue* *component* meets the definition of measurability described above.
- Blastic bone lesions are non-measurable.

**Cystic lesions:**

- Lesions that meet the criteria for radiographically defined simple cysts should not be considered as malignant lesions (neither measurable nor non-measurable) since they are, by definition, simple cysts.
- ‘Cystic lesions’ thought to represent cystic metastases can be considered as measurable lesions, if they meet the definition of measurability described above. However, if non-cystic lesions are present in the same patient, these are preferred for selection as target lesions.

**Lesions with prior local treatment:**

- Tumour lesions situated in a previously irradiated area, or in an area subjected to other loco-regional therapy, are usually not considered measurable unless there has been demonstrated progression in the lesion. Study protocols should detail the conditions under which such lesions would be considered measurable.

3.2. Specifications by methods of measurements

3.2.1. Measurement of lesions

All measurements should be recorded in metric notation, using calipers if clinically assessed. All baseline evaluations should be performed as close as possible to the treatment start and never more than 4 weeks before the beginning of the treatment.

3.2.2. Method of assessment

The same method of assessment and the same technique should be used to characterise each identified and reported lesion at baseline and during follow-up. Imaging based evaluation should always be done rather than clinical examination unless the lesion(s) being followed cannot be imaged but are assessable by clinical exam.*Clinical lesions:* Clinical lesions will only be considered measurable when they are superficial and ⩾10 mm diameter as assessed using calipers (e.g. skin nodules). For the case of skin lesions, documentation by colour photography including a ruler to estimate the size of the lesion is suggested. As noted above, when lesions can be evaluated by both clinical exam and imaging, imaging evaluation should be undertaken since it is more objective and may also be reviewed at the end of the study.*Chest X-ray:* Chest CT is preferred over chest X-ray, particularly when progression is an important endpoint, since CT is more sensitive than X-ray, particularly in identifying new lesions. However, lesions on chest X-ray may be considered measurable if they are clearly defined and surrounded by aerated lung. See [Appendix II](https://www.sciencedirect.com/science/article/pii/S0959804908008733?via%3Dihub#app2) for more details.*CT, MRI:* CT is the best currently available and reproducible method to measure lesions selected for response assessment. This guideline has defined measurability of lesions on CT scan based on the assumption that CT slice thickness is 5 mm or less. As is described in [Appendix II](https://www.sciencedirect.com/science/article/pii/S0959804908008733?via%3Dihub#app2), when CT scans have slice thickness greater than 5 mm, the minimum size for a measurable lesion should be twice the slice thickness. MRI is also acceptable in certain situations (e.g. for body scans). More details concerning the use of both CT and MRI for assessment of objective tumour response evaluation are provided in [Appendix II](https://www.sciencedirect.com/science/article/pii/S0959804908008733?via%3Dihub#app2).*Ultrasound:* Ultrasound is not useful in assessment of lesion size and should not be used as a method of measurement. Ultrasound examinations cannot be reproduced in their entirety for independent review at a later date and, because they are operator dependent, it cannot be guaranteed that the same technique and measurements will be taken from one assessment to the next (described in greater detail in [Appendix II](https://www.sciencedirect.com/science/article/pii/S0959804908008733?via%3Dihub#app2)). If new lesions are identified by ultrasound in the course of the study, confirmation by CT or MRI is advised. If there is concern about radiation exposure at CT, MRI may be used instead of CT in selected instances.*Endoscopy, laparoscopy:* The utilisation of these techniques for objective tumour evaluation is not advised. However, they can be useful to confirm complete pathological response when biopsies are obtained or to determine relapse in trials where recurrence following complete response or surgical resection is an endpoint.*Tumour markers:* Tumour markers *alone* cannot be used to assess *objective* tumour response. If markers are initially above the upper normal limit, however, they must normalise for a patient to be considered in complete response. Because tumour markers are disease specific, instructions for their measurement should be incorporated into protocols on a disease specific basis. Specific guidelines for both CA-125 response (in recurrent ovarian cancer) and PSA response (in recurrent prostate cancer), have been published.[16](https://www.sciencedirect.com/science/article/pii/S0959804908008733?via%3Dihub#bib16), [17](https://www.sciencedirect.com/science/article/pii/S0959804908008733?via%3Dihub#bib17), [18](https://www.sciencedirect.com/science/article/pii/S0959804908008733?via%3Dihub#bib18) In addition, the Gynecologic Cancer Intergroup has developed CA125 progression criteria which are to be integrated with objective tumour assessment for use in first-line trials in ovarian cancer.^[19](https://www.sciencedirect.com/science/article/pii/S0959804908008733?via%3Dihub#bib19)^*Cytology, histology:* These techniques can be used to differentiate between PR and CR in rare cases if required by protocol (for example, residual lesions in tumour types such as germ cell tumours, where known residual benign tumours can remain). When effusions are known to be a potential adverse effect of treatment (e.g. with certain taxane compounds or angiogenesis inhibitor), the cytological confirmation of the neoplastic origin of any effusion that appears or worsens during treatment can be considered if the measurable tumour has met criteria for response or stable disease in order to differentiate between response (or stable disease) and progressive disease.

4. Tumour response evaluation

4.1. Assessment of overall tumour burden and measurable disease

To assess objective response or future progression, it is necessary to estimate the *overall* *tumour* *burden* *at* *baseline* and use this as a comparator for subsequent measurements. Only patients with measurable disease at baseline should be included in protocols where objective tumour response is the primary endpoint. Measurable disease is defined by the presence of at least one measurable lesion (as detailed above in Section [3](https://www.sciencedirect.com/science/article/pii/S0959804908008733?via%3Dihub#sec1)). In studies where the primary endpoint is tumour progression (either time to progression or proportion with progression at a fixed date), the protocol must specify if entry is restricted to those with measurable disease or whether patients having non-measurable disease only are also eligible.

4.2. Baseline documentation of ‘target’ and ‘non-target’ lesions

When more than one measurable lesion is present at baseline all lesions up to a maximum of five lesions total (and a maximum of two lesions per organ) representative of all involved organs should be identified as *target* *lesions* and will be recorded and measured at baseline (this means in instances where patients have only one or two organ sites involved a *maximum* of two and four lesions respectively will be recorded). For evidence to support the selection of only five target lesions, see analyses on a large prospective database in the article by Bogaerts et al.^[10](https://www.sciencedirect.com/science/article/pii/S0959804908008733?via%3Dihub#bib10)^.

Target lesions should be selected on the basis of their size (lesions with the longest diameter), be representative of all involved organs, but in addition should be those that lend themselves to *reproducible* *repeated* *measurements*. It may be the case that, on occasion, the largest lesion does not lend itself to reproducible measurement in which circumstance the next largest lesion which can be measured reproducibly should be selected. To illustrate this point see the example in Fig. 3 of [Appendix II](https://www.sciencedirect.com/science/article/pii/S0959804908008733?via%3Dihub#app2).

*Lymph nodes* merit special mention since they are normal anatomical structures which may be visible by imaging even if not involved by tumour. As noted in Section [3](https://www.sciencedirect.com/science/article/pii/S0959804908008733?via%3Dihub#sec1), pathological nodes which are defined as measurable and may be identified as target lesions must meet the criterion of a short axis of ⩾15 mm by CT scan. Only the *short* axis of these nodes will contribute to the baseline sum. The short axis of the node is the diameter normally used by radiologists to judge if a node is involved by solid tumour. Nodal size is normally reported as two dimensions in the plane in which the image is obtained (for CT scan this is almost always the axial plane; for MRI the plane of acquisition may be axial, saggital or coronal). The smaller of these measures is the short axis. For example, an abdominal node which is reported as being 20 mm × 30 mm has a short axis of 20 mm and qualifies as a malignant, measurable node. In this example, 20 mm should be recorded as the node measurement (See also the example in Fig. 4 in [Appendix II](https://www.sciencedirect.com/science/article/pii/S0959804908008733?via%3Dihub#app2)). All other pathological nodes (those with short axis ⩾10 mm but <15 mm) should be considered non-target lesions. Nodes that have a short axis <10 mm are considered non-pathological and should not be recorded or followed.

A *sum* *of* *the* *diameters* (longest for non-nodal lesions, short axis for nodal lesions) for all target lesions will be calculated and reported as the *baseline* *sum* *diameters*. If lymph nodes are to be included in the sum, then as noted above, only the *short* axis is added into the sum. The baseline sum diameters will be used as reference to further characterise any objective tumour regression in the measurable dimension of the disease.

All other lesions (or sites of disease) including pathological lymph nodes should be identified as *non-target* *lesions* and should also be recorded at baseline. Measurements are not required and these lesions should be followed as ‘present’, ‘absent’, or in rare cases ‘unequivocal progression’ (more details to follow). In addition, it is possible to record multiple non-target lesions involving the same organ as a single item on the case record form (e.g. ‘multiple enlarged pelvic lymph nodes’ or ‘multiple liver metastases’).

4.3. Response criteria

This section provides the definitions of the criteria used to determine objective tumour response for target lesions.

4.3.1. Evaluation of target lesions

Complete Response (CR):

Disappearance of all target lesions. Any pathological lymph nodes (whether target or non-target) must have reduction in short axis to <10 mm.

Partial Response (PR):

At least a 30% decrease in the sum of diameters of target lesions, taking as reference the baseline sum diameters.

Progressive Disease (PD):

At least a 20% increase in the sum of diameters of target lesions, taking as reference the *smallest* *sum* *on* *study* (this includes the baseline sum if that is the smallest on study). In addition to the relative increase of 20%, the sum must also demonstrate an absolute increase of at least 5 mm. (*Note:* the appearance of one or more new lesions is also considered progression).

Stable Disease (SD):

Neither sufficient shrinkage to qualify for PR nor sufficient increase to qualify for PD, taking as reference the smallest sum diameters while on study.

4.3.2. Special notes on the assessment of target lesions

**Lymph nodes**

Lymph nodes identified as target lesions should always have the actual short axis measurement recorded (measured in the same anatomical plane as the baseline examination), even if the nodes regress to below 10 mm on study. This means that when lymph nodes are included as target lesions, the ‘sum’ of lesions may not be zero even if complete response criteria are met, since a normal lymph node is defined as having a short axis of <10 mm. Case report forms or other data collection methods may therefore be designed to have target nodal lesions recorded in a separate section where, in order to qualify for CR, each node must achieve a short axis <10 mm. For PR, SD and PD, the actual short axis measurement of the nodes is to be included in the sum of target lesions.

**Target lesions that become ‘too small to measure’**

While on study, all lesions (nodal and non-nodal) recorded at baseline should have their actual measurements recorded at each subsequent evaluation, even when very small (e.g. 2 mm). However, sometimes lesions or lymph nodes which are recorded as target lesions at baseline become so faint on CT scan that the radiologist may not feel comfortable assigning an exact measure and may report them as being ‘too small to measure’. When this occurs it is important that a value be recorded on the case report form. If it is the opinion of the radiologist that the lesion has likely disappeared, the measurement should be recorded as 0 mm. If the lesion is believed to be present and is faintly seen but too small to measure, a default value of 5 mm should be assigned (*Note:* It is less likely that this rule will be used for lymph nodes since they usually have a definable size when normal and are frequently surrounded by fat such as in the retroperitoneum; however, if a lymph node is believed to be present and is faintly seen but too small to measure, a default value of 5 mm should be assigned in this circumstance as well). This default value is derived from the 5 mm CT slice thickness (but should not be changed with varying CT slice thickness). The measurement of these lesions is potentially non-reproducible, therefore providing this default value will prevent false responses or progressions based upon measurement error. To reiterate, however, if the radiologist *is* able to provide an actual measure, that should be recorded, even if it is below 5 mm.

**Lesions that split or coalesce on treatment**

As noted in [Appendix II](https://www.sciencedirect.com/science/article/pii/S0959804908008733?via%3Dihub#app2), when non-nodal lesions ‘fragment’, the longest diameters of the fragmented portions should be added together to calculate the target lesion sum. Similarly, as lesions coalesce, a plane between them may be maintained that would aid in obtaining maximal diameter measurements of each individual lesion. If the lesions have truly coalesced such that they are no longer separable, the vector of the longest diameter in this instance should be the maximal longest diameter for the ‘coalesced lesion’.

4.3.3. Evaluation of non-target lesions

This section provides the definitions of the criteria used to determine the tumour response for the group of non-target lesions. While some non-target lesions may actually be measurable, they need not be measured and instead should be assessed only *qualitatively* at the time points specified in the protocol.

Complete Response (CR):

Disappearance of all non-target lesions and normalisation of tumour marker level. All lymph nodes must be non-pathological in size (<10 mm short axis).

Non-CR/Non-PD:

Persistence of one or more non-target lesion(s) and/or maintenance of tumour marker level above the normal limits.

Progressive Disease (PD):

*Unequivocal* *progression* (see comments below) of existing non-target lesions. (*Note:* the appearance of one or more new lesions is also considered progression).

4.3.4. Special notes on assessment of progression of non-target disease

The concept of progression of non-target disease requires additional explanation as follows:

**When the patient also has measurable disease**

In this setting, to achieve ‘unequivocal progression’ on the basis of the non-target disease, there must be an overall level of substantial worsening in non-target disease such that, even in presence of SD or PR in target disease, the overall tumour burden has increased sufficiently to merit discontinuation of therapy (see examples in [Appendix II](https://www.sciencedirect.com/science/article/pii/S0959804908008733?via%3Dihub#app2) and further details below). A modest ‘increase’ in the size of one or more non-target lesions is usually not sufficient to quality for unequivocal progression status. The designation of overall progression *solely* on the basis of change in non-target disease in the face of SD or PR of target disease will therefore be extremely rare.

**When the patient has only non-measurable disease**

This circumstance arises in some phase III trials when it is not a criterion of study entry to have measurable disease. The same general concepts apply here as noted above, however, in this instance there is no measurable disease assessment to factor into the interpretation of an increase in non-measurable disease burden. Because worsening in non-target disease cannot be easily quantified (by definition: if all lesions are truly non-measurable) a useful trial that can be applied when assessing patients for unequivocal progression is to consider if the increase in overall disease burden based on the change in non-measurable disease is comparable in magnitude to the increase that would be required to declare PD for measurable disease: i.e. an increase in tumour burden representing an additional 73% increase in ‘volume’ (which is equivalent to a 20% increase diameter in a measurable lesion). Examples include an increase in a pleural effusion from ‘trace’ to ‘large’, an increase in lymphangitic disease from localised to widespread, or may be described in protocols as ‘sufficient to require a change in therapy’. Some illustrative examples are shown in Figs. 5 and 6 in [Appendix II](https://www.sciencedirect.com/science/article/pii/S0959804908008733?via%3Dihub#app2). If ‘unequivocal progression’ is seen, the patient should be considered to have had overall PD at that point. While it would be ideal to have objective criteria to apply to non-measurable disease, the very nature of that disease makes it impossible to do so, therefore the increase must be substantial.

4.3.5. New lesions

The appearance of new malignant lesions denotes disease progression; therefore, some comments on detection of new lesions are important. There are no specific criteria for the identification of new radiographic lesions; however, the finding of a new lesion should be unequivocal: i.e. not attributable to differences in scanning technique, change in imaging modality or findings thought to represent something other than tumour (for example, some ‘new’ bone lesions may be simply healing or flare of pre-existing lesions). This is particularly important when the patient’s baseline lesions show partial or complete response. For example, necrosis of a liver lesion may be reported on a CT scan report as a ‘new’ cystic lesion, which it is not.

A lesion identified on a follow-up study in an anatomical location that was *not* scanned at baseline is considered a new lesion and will indicate disease progression. An example of this is the patient who has visceral disease at baseline and while on study has a CT or MRI brain ordered which reveals metastases. The patient’s brain metastases are considered to be evidence of PD even if he/she did not have brain imaging at baseline.

If a new lesion is equivocal, for example because of its small size, continued therapy and follow-up evaluation will clarify if it represents truly new disease. If repeat scans confirm there is definitely a new lesion, then progression should be declared using the date of the initial scan.

While FDG-PET response assessments need additional study, it is sometimes reasonable to incorporate the use of FDG-PET scanning to complement CT scanning in assessment of progression (particularly possible ‘new’ disease). New lesions on the basis of FDG-PET imaging can be identified according to the following algorithm:

a. Negative FDG-PET at baseline, with a positive^[l](https://www.sciencedirect.com/science/article/pii/S0959804908008733?via%3Dihub#fn1)^ FDG-PET at follow-up is a sign of PD based on a new lesion.

b. No FDG-PET at baseline and a positive FDG-PET at follow-up:

- - If the positive FDG-PET at follow-up corresponds to a new site of disease confirmed by CT, this is PD.
  - If the positive FDG-PET at follow-up is not confirmed as a new site of disease on CT, additional follow-up CT scans are needed to determine if there is truly progression occurring at that site (if so, the date of PD will be the date of the initial abnormal FDG-PET scan).
  - If the positive FDG-PET at follow-up corresponds to a pre-existing site of disease on CT that is not progressing on the basis of the anatomic images, this is not PD.

4.4. Evaluation of best overall response

The best overall response is the best response recorded from the start of the study treatment until the end of treatment taking into account any requirement for confirmation. On occasion a response may not be documented until after the end of therapy so protocols should be clear if post-treatment assessments are to be considered in determination of best overall response. Protocols must specify how any new therapy introduced before progression will affect best response designation. The patient’s best overall response assignment will depend on the findings of both target and non-target disease and will also take into consideration the appearance of new lesions. Furthermore, depending on the nature of the study and the protocol requirements, it may also require confirmatory measurement (see Section [4.6](https://www.sciencedirect.com/science/article/pii/S0959804908008733?via%3Dihub#sec2)). Specifically, in non-randomised trials where response is the primary endpoint, confirmation of PR or CR is needed to deem either one the ‘best overall response’. This is described further below.

4.4.1. Time point response

It is assumed that at each protocol specified time point, a response assessment occurs. [Table 1](https://www.sciencedirect.com/science/article/pii/S0959804908008733?via%3Dihub#tbl1) on the next page provides a summary of the overall response status calculation at each time point for patients who have measurable disease at baseline.

Table 1. Time point response: patients with target (+/– non-target) disease.

| **Target lesions** | **Non-target lesions** | **New lesions** | **Overall response** |
| --- | --- | --- | --- |
| CR | CR | No | CR |
| CR | Non-CR/non-PD | No | PR |
| CR | Not evaluated | No | PR |
| PR | Non-PD or not all evaluated | No | PR |
| SD | Non-PD or not all evaluated | No | SD |
| Not all evaluated | Non-PD | No | NE |
| PD | Any | Yes or No | PD |
| Any | PD | Yes or No | PD |
| Any | Any | Yes | PD |

CR = complete response, PR = partial response, SD = stable disease, PD = progressive disease, and NE = inevaluable.

When patients have non-measurable (therefore non-target) disease only, [Table 2](https://www.sciencedirect.com/science/article/pii/S0959804908008733?via%3Dihub#tbl2) is to be used.

Table 2. Time point response: patients with non-target disease only.

| **Non-target lesions** | **New lesions** | **Overall response** |
| --- | --- | --- |
| CR | No | CR |
| Non-CR/non-PD | No | Non-CR/non-PD^[a](https://www.sciencedirect.com/science/article/pii/S0959804908008733?via%3Dihub#tblfn1)^ |
| Not all evaluated | No | NE |
| Unequivocal PD | Yes or No | PD |
| Any | Yes | PD |

CR = complete response, PD = progressive disease, and NE = inevaluable.

^a^ ‘Non-CR/non-PD’ is preferred over ‘stable disease’ for non-target disease since SD is increasingly used as endpoint for assessment of efficacy in some trials so to assign this category when no lesions can be measured is not advised.

4.4.2. Missing assessments and inevaluable designation

When no imaging/measurement is done at all at a particular time point, the patient is not evaluable (NE) at that time point. If only a subset of lesion measurements are made at an assessment, usually the case is also considered NE at that time point, unless a convincing argument can be made that the contribution of the individual missing lesion(s) would not change the assigned time point response. This would be most likely to happen in the case of PD. For example, if a patient had a baseline sum of 50 mm with three measured lesions and at follow-up only two lesions were assessed, but those gave a sum of 80 mm, the patient will have achieved PD status, regardless of the contribution of the missing lesion.

4.4.3. Best overall response: all time points

The *best* *overall* *response* is determined once all the data for the patient is known.

*Best response determination in trials where confirmation of complete or partial response IS NOT required*: Best response in these trials is defined as the best response across all time points (for example, a patient who has SD at first assessment, PR at second assessment, and PD on last assessment has a best overall response of PR). When SD is believed to be best response, it must also meet the protocol specified minimum time from baseline. If the minimum time is not met when SD is otherwise the best time point response, the patient’s best response depends on the subsequent assessments. For example, a patient who has SD at first assessment, PD at second and does not meet minimum duration for SD, will have a best response of PD. The same patient lost to follow-up after the first SD assessment would be considered inevaluable.

*Best response determination in trials where confirmation of complete or partial response IS required*: Complete or partial responses may be claimed only if the criteria for each are met at a subsequent time point as specified in the protocol (generally 4 weeks later). In this circumstance, the best overall response can be interpreted as in [Table 3](https://www.sciencedirect.com/science/article/pii/S0959804908008733?via%3Dihub#tbl3).

Table 3. Best overall response when confirmation of CR and PR required.

| **Overall response** | **Overall response** | **BEST overall response** |
| --- | --- | --- |
| **First time point** | **Subsequent time point** | Empty Cell |
| CR | CR | CR |
| CR | PR | SD, PD or PR^[a](https://www.sciencedirect.com/science/article/pii/S0959804908008733?via%3Dihub#tblfn2)^ |
| CR | SD | SD provided minimum criteria for SD duration met, otherwise, PD |
| CR | PD | SD provided minimum criteria for SD duration met, otherwise, PD |
| CR | NE | SD provided minimum criteria for SD duration met, otherwise NE |
| PR | CR | PR |
| PR | PR | PR |
| PR | SD | SD |
| PR | PD | SD provided minimum criteria for SD duration met, otherwise, PD |
| PR | NE | SD provided minimum criteria for SD duration met, otherwise NE |
| NE | NE | NE |

CR = complete response, PR = partial response, SD = stable disease, PD = progressive disease, and NE = inevaluable.

^a^ If a CR is *truly* met at first time point, then any disease seen at a subsequent time point, even disease meeting PR criteria relative to baseline, makes the disease PD at that point (since disease must have reappeared after CR). Best response would depend on whether minimum duration for SD was met. However, sometimes ‘CR’ may be claimed when subsequent scans suggest small lesions were likely still present and in fact the patient had PR, not CR at the first time point. Under these circumstances, the original CR should be changed to PR and the best response is PR.

4.4.4. Special notes on response assessment

When nodal disease is included in the sum of target lesions and the nodes decrease to ‘normal’ size (<10 mm), they may still have a measurement reported on scans. This measurement should be recorded even though the nodes are normal in order not to overstate progression should it be based on increase in size of the nodes. As noted earlier, this means that patients with CR may not have a total sum of ‘zero’ on the case report form (CRF).

In trials where confirmation of response is required, repeated ‘NE’ time point assessments may complicate best response determination. The analysis plan for the trial must address how missing data/assessments will be addressed in determination of response and progression. For example, in most trials it is reasonable to consider a patient with time point responses of PR-NE-PR as a confirmed response.

Patients with a global deterioration of health status requiring discontinuation of treatment without objective evidence of disease progression at that time should be reported as ‘symptomatic deterioration’. Every effort should be made to document objective progression even after discontinuation of treatment. Symptomatic deterioration is *not* a descriptor of an objective response: it is a reason for stopping study therapy. The objective response status of such patients is to be determined by evaluation of target and non-target disease as shown in [Table 1](https://www.sciencedirect.com/science/article/pii/S0959804908008733?via%3Dihub#tbl1), [Table 2](https://www.sciencedirect.com/science/article/pii/S0959804908008733?via%3Dihub#tbl2), [Table 3](https://www.sciencedirect.com/science/article/pii/S0959804908008733?via%3Dihub#tbl3).

Conditions that define ‘early progression, early death and inevaluability’ are study specific and should be clearly described in each protocol (depending on treatment duration, treatment periodicity).

In some circumstances it may be difficult to distinguish residual disease from normal tissue. When the evaluation of complete response depends upon this determination, it is recommended that the residual lesion be investigated (fine needle aspirate/biopsy) before assigning a status of complete response. FDG-PET may be used to upgrade a response to a CR in a manner similar to a biopsy in cases where a residual radiographic abnormality is thought to represent fibrosis or scarring. The use of FDG-PET in this circumstance should be prospectively described in the protocol and supported by disease specific medical literature for the indication. However, it must be acknowledged that both approaches may lead to false positive CR due to limitations of FDG-PET and biopsy resolution/sensitivity.

For equivocal findings of progression (e.g. very small and uncertain new lesions; cystic changes or necrosis in existing lesions), treatment may continue until the next scheduled assessment. If at the next scheduled assessment, progression is confirmed, the date of progression should be the earlier date when progression was suspected.

4.5. Frequency of tumour re-evaluation

Frequency of tumour re-evaluation while on treatment should be protocol specific and adapted to the type and schedule of treatment. However, in the context of phase II studies where the beneficial effect of therapy is not known, follow-up every 6–8 weeks (timed to coincide with the end of a cycle) is reasonable. Smaller or greater time intervals than these could be justified in specific regimens or circumstances. The protocol should specify which organ sites are to be evaluated at baseline (usually those most likely to be involved with metastatic disease for the tumour type under study) and how often evaluations are repeated. Normally, all target and non-target sites are evaluated at each assessment. In selected circumstances certain non-target organs may be evaluated less frequently. For example, bone scans may need to be repeated only when complete response is identified in target disease or when progression in bone is suspected.

After the end of the treatment, the need for repetitive tumour evaluations depends on whether the trial has as a goal the response rate or the time to an event (progression/death). If ‘time to an event’ (e.g. time to progression, disease-free survival, progression-free survival) is the main endpoint of the study, then routine scheduled re-evaluation of protocol specified sites of disease is warranted. In randomised comparative trials in particular, the scheduled assessments should be performed as identified on a calendar schedule (for example: every 6–8 weeks on treatment or every 3–4 months after treatment) and should not be affected by delays in therapy, drug holidays or any other events that might lead to imbalance in a treatment arm in the timing of disease assessment.

4.6. Confirmatory measurement/duration of response

4.6.1. Confirmation

In non-randomised trials where response is the primary endpoint, confirmation of PR and CR is required to ensure responses identified are not the result of measurement error. This will also permit appropriate interpretation of results in the context of historical data where response has traditionally required confirmation in such trials (see the paper by Bogaerts et al. in this Special Issue[^10^](https://www.sciencedirect.com/science/article/pii/S0959804908008733?via%3Dihub#bib10)). However, in all other circumstances, i.e. in randomised trials (phase II or III) or studies where stable disease or progression are the primary endpoints, confirmation of response is not required since it will not add value to the interpretation of trial results. However, elimination of the requirement for response confirmation may increase the importance of central review to protect against bias, in particular in studies which are not blinded.

In the case of SD, measurements must have met the SD criteria at least once after study entry at a minimum interval (in general not less than 6–8 weeks) that is defined in the study protocol.

4.6.2. Duration of overall response

The duration of overall response is measured from the time measurement criteria are first met for CR/PR (whichever is first recorded) until the first date that recurrent or progressive disease is objectively documented (taking as reference for progressive disease the smallest measurements recorded on study).

The duration of overall complete response is measured from the time measurement criteria are first met for CR until the first date that recurrent disease is objectively documented.

4.6.3. Duration of stable disease

Stable disease is measured from the start of the treatment (in randomised trials, from date of randomisation) until the criteria for progression are met, taking as reference the *smallest* *sum* *on* *study* (if the baseline sum is the smallest, this is the reference for calculation of PD).

The clinical relevance of the duration of stable disease varies in different studies and diseases. If the proportion of patients achieving stable disease for a minimum period of time is an endpoint of importance in a particular trial, the protocol should specify the minimal time interval required between two measurements for determination of stable disease.

*Note*: The duration of response and stable disease as well as the progression-free survival are influenced by the frequency of follow-up after baseline evaluation. It is not in the scope of this guideline to define a standard follow-up frequency. The frequency should take into account many parameters including disease types and stages, treatment periodicity and standard practice. However, these limitations of the precision of the measured endpoint should be taken into account if comparisons between trials are to be made.

4.7. Progression-free survival/proportion progression-free

4.7.1. Phase II trials

This guideline is focused primarily on the use of objective response endpoints for phase II trials. In some circumstances, ‘response rate’ may not be the optimal method to assess the potential anticancer activity of new agents/regimens. In such cases ‘progression-free survival’ (PFS) or the ‘proportion progression-free’ at landmark time points, might be considered appropriate alternatives to provide an initial signal of biologic effect of new agents. It is clear, however, that in an uncontrolled trial, these measures are subject to criticism since an apparently promising observation may be related to biological factors such as patient selection and not the impact of the intervention. Thus, phase II screening trials utilising these endpoints are best designed with a randomised control. Exceptions may exist where the behaviour patterns of certain cancers are so consistent (and usually consistently poor), that a non-randomised trial is justifiable (see for example van Glabbeke et al.^[20](https://www.sciencedirect.com/science/article/pii/S0959804908008733?via%3Dihub#bib20)^). However, in these cases it will be essential to document with care the basis for estimating the expected PFS or proportion progression-free in the absence of a treatment effect.

4.7.2. Phase III trials

Phase III trials in advanced cancers are increasingly designed to evaluate progression-free survival or time to progression as the primary outcome of interest. Assessment of progression is relatively straightforward if the protocol requires all patients to have measurable disease. However, restricting entry to this subset of patients is subject to criticism: it may result in a trial where the results are less likely to be generalisable if, in the disease under study, a substantial proportion of patients would be excluded. Moreover, the restriction to entry will slow recruitment to the study. Increasingly, therefore, trials allow entry of both patients with measurable disease as well as those with non-measurable disease only. In this circumstance, care must be taken to explicitly describe the findings which would qualify for progressive disease for those patients *without* measurable lesions. Furthermore, in this setting, protocols must indicate if the maximum number of recorded target lesions for those patients with measurable disease may be relaxed from five to three (based on the data found in Bogaerts et al.[^10^](https://www.sciencedirect.com/science/article/pii/S0959804908008733?via%3Dihub#bib10) and Moskowitz et al.^[11](https://www.sciencedirect.com/science/article/pii/S0959804908008733?via%3Dihub#bib11)^). As found in the ‘special notes on assessment of progression’, these guidelines offer recommendations for assessment of progression in this setting. Furthermore, if available, validated tumour marker measures of progression (as has been proposed for ovarian cancer) may be useful to integrate into the definition of progression. Centralised blinded review of imaging studies or of source imaging reports to verify ‘unequivocal progression’ may be needed if important drug development or drug approval decisions are to be based on the study outcome. Finally, as noted earlier, because the date of progression is subject to ascertainment bias, timing of investigations in study arms should be the same. The article by Dancey et al. in this special issue^[21](https://www.sciencedirect.com/science/article/pii/S0959804908008733?via%3Dihub#bib21)^ provides a more detailed discussion of the assessment of progression in randomised trials.

4.8. Independent review of response and progression

For trials where *objective* *response* (CR + PR) is the primary endpoint, and in particular where key drug development decisions are based on the observation of a minimum number of responders, it is recommended that all claimed responses be reviewed by an expert(s) independent of the study. If the study is a randomised trial, ideally reviewers should be blinded to treatment assignment. Simultaneous review of the patients’ files and radiological images is the best approach.

Independent review of progression presents some more complex issues: for example, there are statistical problems with the use of central-review-based progression time in place of investigator-based progression time due to the potential introduction of informative censoring when the former precedes the latter. An overview of these factors and other lessons learned from independent review is provided in an article by Ford et al. in this special issue.^[22](https://www.sciencedirect.com/science/article/pii/S0959804908008733?via%3Dihub#bib22)^

4.9. Reporting best response results

4.9.1. Phase II trials

When response is the primary endpoint, and thus all patients must have measurable disease to enter the trial, all patients included in the study must be accounted for in the report of the results, even if there are major protocol treatment deviations or if they are not evaluable. Each patient will be assigned one of the following categories:

1. Complete response

2. Partial response

3. Stable disease

4. Progression

5. Inevaluable for response: specify reasons (for example: early death, malignant disease; early death, toxicity; tumour assessments not repeated/incomplete; other (specify)).

Normally, all *eligible* patients should be included in the denominator for the calculation of the response rate for phase II trials (in some protocols it will be appropriate to include all treated patients). It is generally preferred that 95% two-sided confidence limits are given for the calculated response rate. Trial conclusions should be based on the response rate for all eligible (or all treated) patients and should *not* be based on a selected ‘evaluable’ subset.

4.9.2. Phase III trials

Response evaluation in phase III trials may be an indicator of the relative anti-tumour activity of the treatments evaluated and is almost always a secondary endpoint. Observed differences in response rate may not predict the clinically relevant therapeutic benefit for the population studied. If objective response is selected as a primary endpoint for a phase III study (only in circumstances where a direct relationship between objective tumour response and a clinically relevant therapeutic benefit can be unambiguously demonstrated for the population studied), the same criteria as those applying to phase II trials should be used and all patients entered should have at least one measurable lesion.

In those many cases where response is a secondary endpoint and not all trial patients have measurable disease, the method for reporting overall best response rates must be pre-specified in the protocol. In practice, response rate may be reported using either an ‘intent to treat’ analysis (all randomised patients in the denominator) or an analysis where only the subset of patients with measurable disease at baseline are included. The protocol should clearly specify how response results will be reported, including any subset analyses that are planned.

The original version of RECIST suggested that in phase III trials one could write protocols using a ‘relaxed’ interpretation of the RECIST guidelines (for example, reducing the number of lesions measured) but this should no longer be done since these revised guidelines have been amended in such a way that it is clear how these criteria should be applied for all trials in which anatomical assessment of tumour response or progression are endpoints.
