# Supplementary material for: Consolidative stereotactic radiotherapy for oligo-residual non-small cell lung cancer after first-line chemoimmunotherapy: A single-arm, phase 2 trial from China
Source: PLoS Med. 2025 Aug 1;22(8):e1004680. doi: 10.1371/journal.pmed.1004680 (PMC12316271; doi:10.1371/journal.pmed.1004680)
Supplement: S2 Protocol — (DOCX) [file pmed.1004680.s009.docx]

​**​**

**Clinical Trial Protocol​**​

​**​**

**Real-world efficacy and safety of immune checkpoint inhibitors for solid tumors: A multicenter, prospective, observational study**

​**​Protocol Version Number:​**​ 1.0

​**​Protocol Version Date:​**​ 2020.09.01

​**​1. Study Title​**​

Real-world efficacy and safety of immune checkpoint inhibitors for solid tumors: A multicenter, prospective, observational study

​**​2. Study Background​**​

Malignant tumors are currently a major disease threatening human life and health. According to statistics, there were approximately 18.1 million new cancer cases and 9.6 million cancer-related deaths worldwide in 2018. Among them, China had 3.804 million new cancer patients and approximately 2.296 million deaths, accounting for 21.0% and 23.9% of global new cases and cancer deaths, respectively [1]. Currently, the treatment of malignant tumors remains a global challenge. In recent years, although the three conventional treatment modalities for cancer (surgery, radiotherapy, and chemotherapy) have made significant progress, the survival rate and quality of life of malignant tumor patients still need further improvement. Notably, the application of immune checkpoint inhibitors (ICIs) has been increasingly prominent in cancer treatment. Tumor immunotherapy, represented by ICIs, is one of the most promising research directions in the current field of cancer treatment and is gradually rewriting the diagnosis and treatment guidelines for major solid tumors.

Unlike previous treatment strategies targeting tumor cells, immunotherapy activates and enhances the patient's own anti-tumor immune response to eliminate tumor cells in the body. Normally, the body's immune system has the ability to recognize "self" and eliminate "non-self." During the occurrence and development of tumors, a large number of mutations accumulate, and these mutations can encode numerous tumor-associated antigens. Antigen-presenting cells (APCs) such as dendritic cells (DCs), after recognizing and phagocytosing tumor-associated antigens, can migrate to draining lymph nodes to present tumor antigens to T cells and B cells, promoting the activation and proliferation of T cells and B cells into tumor-specific T cells and tumor-specific B cells. Activated B cells and T cells can migrate to the tumor site and, after specific receptor recognition, kill tumor cells through various mechanisms [2]. However, tumor cells can also evade recognition and attack by the immune system through various mechanisms [3]. Among them, loss of immunogenicity is one of the main mechanisms of tumor immune escape. Additionally, tumor cells can inhibit T cell activation and proliferation by upregulating negative regulatory molecules (such as immune checkpoints) or their ligands, leading to T-cell exhaustion in the tumor microenvironment, thereby inducing immune tolerance and preventing the body's immune system from performing normal immune surveillance and killing functions [4].

In recent years, the application of ICIs to block the transmission of immunosuppressive signals, restore T-cell anti-tumor activity, and thereby reverse the body's immune tolerance state has become a research hotspot in the field of immunotherapy. Currently, cytotoxic T-lymphocyte-associated antigen 4 (CTLA-4), programmed cell death protein 1 (PD-1), and its ligand (PD-L1) are two relatively mature immune checkpoints under investigation. The co-stimulatory molecules CD80 and CD86 on the surface of APCs can bind to the co-stimulatory molecule receptor CD28 on the T cell surface, providing a second signal for T cell activation. CTLA-4 is mainly expressed on the surface of activated T cells and has high homology with CD28. Therefore, CTLA-4 can also bind to CD80 and CD86 on the surface of APCs, preventing CD28-mediated co-stimulatory signal transduction and transmitting inhibitory signals to T cells [5]. Furthermore, CTLA-4 is constitutively expressed in regulatory T cells (Treg) and plays a key role in Treg-mediated anti-tumor immune suppression [6]. Anti-CTLA-4 antibodies can block the binding of CTLA-4 to its ligands, reversing its immunosuppressive effect and thereby enhancing the body's anti-tumor immune response.

In addition to CTLA-4, PD-1 expressed on activated T cells is another important immune checkpoint. When PD-1 binds to its ligand PD-L1, the tyrosine in the ITSM domain of PD-1 is phosphorylated, recruiting SHP2 phosphatase molecules that dephosphorylate downstream effector molecules, thereby inhibiting T cell proliferation, differentiation, and cytokine production, exerting a negative regulatory effect [7,8]. Tumor cells can upregulate the expression of PD-1 ligands PD-L1 and PD-L2 (mainly PD-L1). Binding of PD-L1/PD-L2 to the PD-1 receptor on tumor-infiltrating lymphocytes inhibits the proliferation and activation of cytotoxic T cells, preventing them from effectively exerting anti-tumor effects, thereby altering the tumor microenvironment and ultimately leading to tumor immune escape [9]. Furthermore, the PD-1/PD-L1 signaling pathway also plays an important role in Treg differentiation and their immunosuppressive function [10]. Monoclonal antibodies targeting PD-1 or PD-L1 can block the binding of PD-L1 on the tumor cell surface to PD-1 on the T cell surface, releasing T-cell inhibition and enhancing anti-tumor immune effects.

An increasing number of large-scale prospective clinical trials have shown that compared with traditional therapies, ICIs can bring significant survival benefits to patients with malignant tumors (such as lung cancer, advanced melanoma, esophageal squamous cell carcinoma, etc.) [11-13]. Currently, anti-PD-1 monoclonal antibodies such as Nivolumab and Pembrolizumab, and the PD-L1-targeted monoclonal antibody Durvalumab have been approved for marketing in mainland China by the National Medical Products Administration (NMPA), opening a new era of solid tumor treatment in China. Nivolumab has shown significant efficacy against advanced melanoma, non-small cell lung cancer (NSCLC), renal cancer, etc [14-16]. In the Phase III clinical trials Checkmate-017 [15] and Checkmate-057 [17], Nivolumab compared with standard docetaxel second-line chemotherapy significantly improved overall survival (OS) in NSCLC patients with squamous and non-squamous histology who progressed after first-line chemotherapy: in squamous patients, the median OS in the Nivolumab group was 3.2 months longer than in the chemotherapy group (9.2 months vs. 6.0 months); in non-squamous patients, the median OS in the Nivolumab group was 2.8 months longer than in the chemotherapy group (12.2 months vs. 9.4 months). Other PD-1/PD-L1 antibodies such as Pembrolizumab are also widely used in the treatment of advanced melanoma and NSCLC, etc., and are gradually advancing to first-line treatment. The results of the Keynote-024 study [18] showed that in NSCLC patients with PD-L1 expression ≥50% and no EGFR/ALK mutations, first-line treatment with Pembrolizumab significantly improved OS and progression-free survival (PFS) compared to standard platinum-based chemotherapy. Based on the Keynote-024 study, the Keynote-042 study [19] enrolled PD-L1 ≥1% driver gene-negative advanced NSCLC patients to explore the efficacy of Pembrolizumab monotherapy as first-line treatment. The study found that median OS in the Pembrolizumab group was significantly better than in the chemotherapy group, with the most significant efficacy observed in patients with PD-L1 ≥50%. Concurrently in the field of NSCLC treatment, the success of the PACIFIC study [20] extended the application of ICIs from advanced patients to locally advanced patients. In that study, consolidation therapy with Durvalumab after concurrent chemoradiotherapy significantly improved PFS in patients with locally advanced, unresectable stage III NSCLC (16.8 months vs. 5.6 months, P<0.001). In terms of survival rate, the 24-month overall survival rate in the Durvalumab group was also much higher than in the placebo group (66.3% vs. 55.6%, P=0.005). Currently, ICIs have been approved for more than 25 indications worldwide, and they are expected to be game-changers in overcoming the dilemma of cancer treatment, bringing new hope to an increasing number of cancer patients.

However, while ICIs have made remarkable progress and achieved significant results in the field of solid tumor treatment, many questions remain to be answered, including predictive biomarkers for ICI efficacy, the mechanisms and prevention/treatment measures for immune-related adverse events (irAEs), mechanisms of resistance, optimal treatment models (such as duration of ICI therapy), advantages and disadvantages of combining ICIs with different treatment modalities (chemotherapy, radiotherapy, targeted therapy, etc.), and the optimal patient populations corresponding to various treatment models. Currently, reports on the efficacy and safety of ICI treatment in solid tumor patients mostly come from randomized controlled trials (RCTs). However, RCTs determine a specific population of a certain sample size through a series of inclusion and exclusion criteria. These strict conditions limit the applicability and generalizability of RCT results in real-world clinical practice [21]. Real-world studies (RWS) collect data related to patients in real-world environments and, through analysis, obtain clinical evidence on the value of use and potential benefits or risks of medical products. The type of RWS can be observational studies or clinical trials. Compared to RCTs, RWS is rooted in real clinical practice, its data comes from a broader patient population and different treatment scenarios, and thus it is closer to clinical reality and better reflects the treatment situation in the real world [21].

Therefore, this study aims to explore some urgent problems in the field of ICI treatment, such as the efficacy prediction indicators, irAE mechanisms and prevention/treatment measures, resistance mechanisms, and optimal treatment models mentioned above, through a multicenter observational study based on a real-world big data platform, collecting clinical information from solid tumor patients receiving ICI treatment. It will provide real-world data and evidence for the application and optimization of ICIs in solid tumor treatment.

​**​References​**​

1. Bray, F., et al., Global cancer statistics 2018: GLOBOCAN estimates of incidence and mortality worldwide for 36 cancers in 185 countries. CA Cancer J Clin, 2018. 68(6): p. 394-424.
2. Desrichard, A., A. Snyder, and T.A. Chan, Cancer Neoantigens and Applications for Immunotherapy. Clin Cancer Res, 2016. 22(4): p. 807-12.
3. Beatty, G.L. and W.L. Gladney, Immune escape mechanisms as a guide for cancer immunotherapy. Clin Cancer Res, 2015. 21(4): p. 687-92.
4. Korman, A.J., K.S. Peggs, and J.P. Allison, Checkpoint blockade in cancer immunotherapy. Adv Immunol, 2006. 90: p. 297-339.
5. Carreno, B.M., et al., CTLA-4(CD152) can inhibit T cell activation by two different mechanisms depending on its level of cell surface expression. J Immunol, 2000. 165(3): p. 1352-6.
6. Wing, K., et al., CTLA-4 control over Foxp3+ regulatory T cell function. Science, 2008. 322(5899): p. 271-5.
7. Chemnitz, J.M., et al., SHP-1 and SHP-2 associate with immunoreceptor tyrosine-based switch motif of programmed death 1 upon primary human T cell stimulation, but only receptor ligation prevents T cell activation. J Immunol, 2004. 173(2): p. 945-54.
8. Riley, J.L., PD-1 signaling in primary T cells. Immunol Rev, 2009. 229(1): p. 114-25.
9. Ramsay, A.G., Immune checkpoint blockade immunotherapy to activate anti-tumour T-cell immunity. Br J Haematol, 2013. 162(3): p. 313-25.
10. Cai, J., et al., The Role Of PD-1/PD-L1 Axis In Treg Development And Function: Implications For Cancer Immunotherapy. Onco Targets Ther, 2019. 12: p. 8437-8445.
11. Hamid, O., et al., Safety and tumor responses with lambrolizumab (anti-PD-1) in melanoma. N Engl J Med, 2013. 369(2): p. 134-44.
12. Herbst, R.S., et al., Pembrolizumab versus docetaxel for previously treated, PD-L1-positive, advanced non-small-cell lung cancer (KEYNOTE-010): a randomised controlled trial. Lancet, 2016. 387(10027): p. 1540-1550.
13. Pembrolizumab versus chemotherapy as second-line therapy for advanced esophageal cancer: Phase III KEYNOTE-181 study. 2019 Gastrointestinal Cancers Symposium. Abstract #2.
14. Topalian, S.L., et al., Survival, durable tumor remission, and long-term safety in patients with advanced melanoma receiving nivolumab. J Clin Oncol, 2014. 32(10): p. 1020-30.
15. Brahmer, J., et al., Nivolumab versus Docetaxel in Advanced Squamous-Cell Non-Small-Cell Lung Cancer. N Engl J Med, 2015. 373(2): p. 123-35.
16. Motzer, R.J., et al., Nivolumab plus Ipilimumab versus Sunitinib in Advanced Renal-Cell Carcinoma. N Engl J Med, 2018. 378(14): p. 1277-1290.
17. Borghaei, H., et al., Nivolumab versus Docetaxel in Advanced Nonsquamous Non-Small-Cell Lung Cancer. N Engl J Med, 2015. 373(17): p. 1627-39.
18. Reck, M., et al., Updated Analysis of KEYNOTE-024: Pembrolizumab Versus Platinum-Based Chemotherapy for Advanced Non-Small-Cell Lung Cancer With PD-L1 Tumor Proportion Score of 50% or Greater. J Clin Oncol, 2019. 37(7): p. 537-546.
19. Mok, T.S.K., et al., Pembrolizumab versus chemotherapy for previously untreated, PD-L1-expressing, locally advanced or metastatic non-small-cell lung cancer (KEYNOTE-042): a randomised, open-label, controlled, phase 3 trial. Lancet, 2019. 393(10183): p. 1819-1830.
20. Antonia, S.J., et al., Overall Survival with Durvalumab after Chemoradiotherapy in Stage III NSCLC. N Engl J Med, 2018. 379(24): p. 2342-2350.
21. Sherman, R.E., et al., Real-World Evidence - What Is It and What Can It Tell Us? N Engl J Med, 2016. 375(23): p. 2293-2297.

​**​3. Study Objectives​**​

To analyze the efficacy and safety of immune checkpoint inhibitor therapy for solid tumors in the real-world settings, and to explore urgent scientific issues in tumor treatment with immune checkpoint inhibitors, such as efficacy prediction, occurrence and treatment measures for adverse reactions, resistance mechanisms, and optimal combinational strategies.

​**​4. Study Endpoints​**​

- ​**​Overall Survival (OS):​**​ Time from the start of immune checkpoint inhibitor treatment until death from any cause. Patients still alive at the time of analysis will be censored at the date of their last contact.
- ​**​Objective Response Rate (ORR):​**​ Proportion of patients whose best overall response is Complete Response (CR) or Partial Response (PR) according to the RECIST 1.1 criteria among all evaluable cases.
- ​**​Progression-Free Survival (PFS):​**​ Time from the start of immune checkpoint inhibitor treatment to the observation of disease progression or death from any cause. Patients still alive without progression at the time of analysis will be censored at the date of their last contact.
- ​**​Incidence of Treatment-Related Adverse Events:** Proportion of treatment-related toxic reactions evaluated according to CTCAE 5.0 criteria among all evaluable cases.
- ​**​Pattern of Failure:​**​ Characteristics of tumor regression after immune checkpoint inhibitor treatment, distribution of residual lesions, phenotype of disease progression, and related predictive factors.
- ​**​Biomarkers:​**​ Exploration of the correlation between clinically available pathological indicators, peripheral blood parameters, etc., and the efficacy, toxicity, and disease phenotype of immune checkpoint inhibitor therapy for solid tumors.

​**​5. Case Selection​**​

​**​5.1 Inclusion Criteria​**​

(1) Age ≥ 18 years.
(2) Pathologically confirmed malignant solid tumor, especially lung cancers.
(3) Receiving immune checkpoint inhibitor therapy, either as monotherapy or combinational therapy.
(4) Patients must be able to understand and voluntarily sign the informed consent form.

​**​5.2 Exclusion Criteria​**​

(1) Age < 18 years.
(2) Hematologic malignancies or benign tumors.
(3) Patients with psychiatric disorders, substance abuse, or social problems that affect compliance, as determined by the reviewing physician.

​**​6. Sample Size​**​

This is a multicenter, prospective, observational study and is planned to enroll a total of 2000 patients in 3 years.

​**​7. Study Plan​**​

​**​7.1 Study Design​**​

This is an open-label, prospective, multicenter observational study aimed at exploring the efficacy, safety and patterns of failure of immune checkpoint inhibitors in the treatment of solid tumors. Data will be gathered from solid tumor patients receiving immune checkpoint inhibitor therapy, including age, gender, tumor type, disease stage, treatment methods, laboratory/imaging results, efficacy evaluation, and safety data. The analysis will explore scientific issues such as the efficacy and its predictive indicators, adverse reactions and their treatment outcomes, resistance mechanisms, and clinical phenotypes of immunotherapy in the real-world setting. The study design is as follows:

​**​**Enroll all malignant solid tumor patients (especially lung cancer patients) receiving immune checkpoint inhibitor therapy between September 1, 2020, and August 30, 2023 (approximately 2000 cases) for in-depth, multi-angle, prospective research.

​**​7.2 Patient Enrollment​**​

All malignant solid tumor patients meeting the inclusion/exclusion criteria and receiving immune checkpoint inhibitor therapy can be enrolled in this study. There are no restrictions on tumor type, disease stage, type of immune checkpoint inhibitor, or treatment line.

​**​7.3 Data Collection​**​

Clinical data for enrolled patients will be collected without additional intervention, either through medical record review or telephone follow-up. It includes the following parts:

(1) Basic Information: Age, gender, past medical history, personal history, symptoms, diagnostic methods, pathological reports, disease stage, etc.;
(2) Laboratory/Imaging Results: Tests and examinations required for this study are those needed in routine clinical diagnosis and treatment; there are no additional interventions or extra tests/examinations. Laboratory results include blood routine, liver function, renal function, electrolytes, coagulation function, tumor markers, thyroid function, cytokines, lymphocyte subsets, etc.; Imaging results include chest CT, electrocardiogram (ECG), cardiac ultrasound, pulmonary function, etc.;
(3) Treatment Information: Name of immune checkpoint inhibitor, treatment regimen (monotherapy or combination), line of therapy, number of treatment cycles, efficacy evaluation, etc.;
(4) Follow-up Information: Progression-free survival (PFS), overall survival (OS), adverse events, quality of life assessment, etc.

​**​7.4 Biological Specimens​**​

1. Peripheral Blood Specimens: As this is a non-interventional study, if blood specimens are needed during the trial, they will be obtained by collecting residual blood samples from routine clinical practice; no additional blood draws will be performed.
2. Tissue Specimens: As this is a non-interventional study, if tissue specimens are needed during the trial, they will be obtained from existing sample banks; no additional tissue biopsies will be performed.

​**​7.5 Study Flowchart​**​


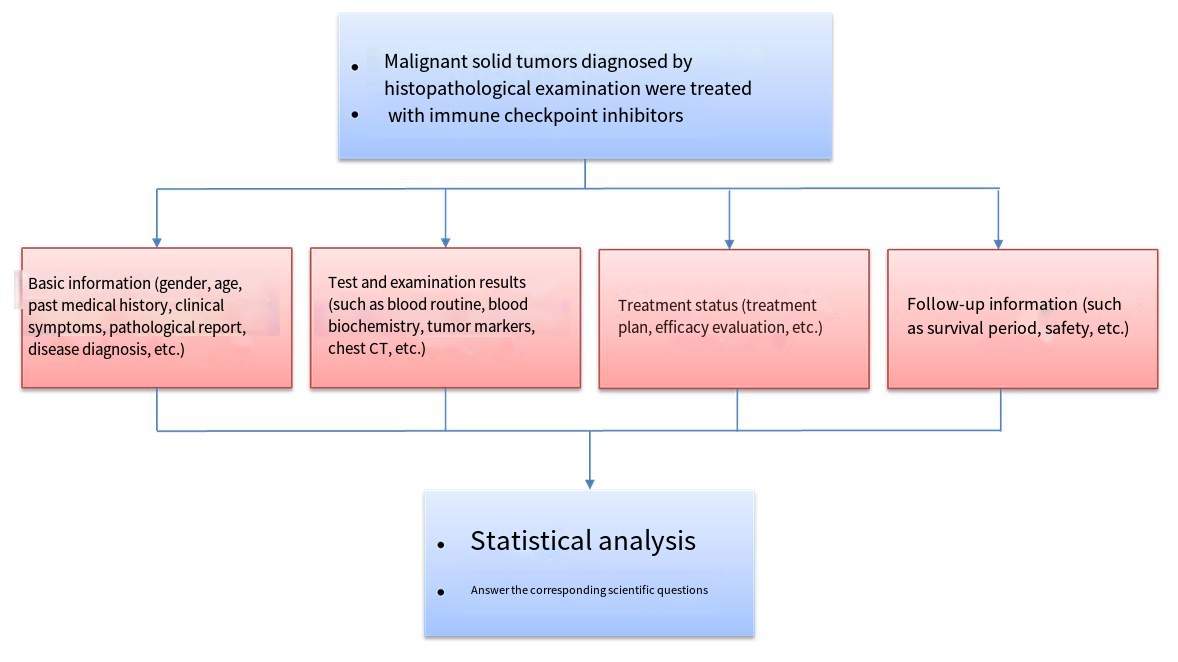


​**​7.6 Follow-up Observation​**​

1. ​**​Follow-up Time​**​

Patients will be generally followed up every 6 weeks. During the first 4 evaluation cycles (24 weeks) of the study period, data collection and follow-up (content as above) will be performed every evaluation cycle (6 weeks); thereafter, data collection and follow-up will be performed every 2 evaluation cycles (12 weeks); after disease progression or discontinuation of immune checkpoint inhibitor therapy, data collection and follow-up will be performed every 12 weeks.

​**​(2) Follow-up Content​**​

- Treatment Information: Use of immune checkpoint inhibitors, drug name, treatment regimen (monotherapy or combination), line of therapy, number of cycles, efficacy evaluation, etc.;
- Survival Information: Progression-free survival (PFS), overall survival (OS), etc.;
- Safety Follow-up: Adverse event recording, quality of life assessment, etc.;
- Laboratory and Imaging Information: Blood routine, blood biochemistry, tumor markers, chest CT, ECG, etc. Tests and examinations required for this study are those needed in actual clinical diagnosis and treatment; there is no additional test or examination content;
- Other Content: Other anti-tumor treatments, concomitant medications, etc.

​**​7.7 Study Drug Information​**​

Currently marketed immune checkpoint inhibitors applied in clinical treatment in China include: Nivolumab, Pembrolizumab, Atezolizumab, Durvalumab, Camrelizumab, Sintilimab, Toripalimab, and Tislelizumab.

​**​(1) Nivolumab​**​

- Brand Name: Opdivo
- Generic Name: Nivolumab Injection
- Active Ingredient: Nivolumab, a humanized monoclonal antibody (IgG4 subtype) targeting programmed death-1 (PD-1)
- Excipients: Disodium citrate dihydrate, Sodium Chloride, Mannitol, Pentetic Acid, Polysorbate 80, Hydrochloric Acid, Sodium Hydroxide, Water for Injection
- Usage and Dosage: Recommended dose is 3mg/kg, intravenous infusion every 2 weeks, each infusion lasting 60 minutes, until disease progression or unacceptable toxicity.
- Adverse Reactions: Fatigue, rash, pruritus, diarrhea, nausea, etc.
- Contraindications: Hypersensitivity to the active ingredient or any excipient listed.
- Storage Conditions: Store at 2~8°C protected from light. Do not freeze.
- Shelf Life: Unopened: 36 months. After opening: From a microbiological perspective, the product should be administered immediately after opening or diluted for infusion. The prepared solution should be used immediately from a microbiological perspective. If the prepared solution cannot be used immediately, stability studies have shown that it can be stored at 2~8°C protected from light for up to 24 hours, or at 20~25°C under ambient light for up to 8 hours (including administration time).
- Manufacturer: Bristol-Myers Squibb Holdings Pharma, Ltd. Liability Company

​**​(2) Pembrolizumab​**​

- Brand Name: Keytruda
- Generic Name: Pembrolizumab Injection
- Active Ingredient: Pembrolizumab
- Excipients: L-Histidine, Sucrose, Polysorbate 80, Water for Injection
- Usage and Dosage: The recommended dosage of Pembrolizumab is 2 mg/kg administered as an intravenous infusion over 30 minutes every 3 weeks until disease progression or unacceptable toxicity.
- Adverse Reactions: Fatigue, pruritus, rash, diarrhea, nausea, etc.
- Contraindications: Hypersensitivity to the active ingredient or excipients listed in the [Ingredients] section.
- Storage Conditions: Store vials refrigerated at 2°C to 8°C in the original carton to protect from light. Do not freeze. Do not shake.
- Shelf Life: 24 months
- Manufacturer: Merck Sharp & Dohme Corp., a subsidiary of Merck & Co., Inc.

​ ​**​(3) Atezolizumab​**​

- Brand Name: Tecentriq
- Generic Name: Atezolizumab Injection
- Active Ingredient: Atezolizumab, a humanized immunoglobulin G1 monoclonal antibody targeting programmed death-ligand 1 (PD-L1)
- Excipients: L-Histidine, Acetic Acid glacial, Sucrose, Polysorbate 20, Water for Injection
- Usage and Dosage: The recommended dosage of Atezolizumab is 1200 mg administered as an intravenous infusion. The first infusion should last at least 60 minutes; subsequent infusions should last at least 30 minutes, administered every 3 weeks until disease progression or unacceptable toxicity.
- Adverse Reactions: Fatigue, decreased appetite, nausea, cough, etc.
- Contraindications: Hypersensitivity to the active ingredient or excipients listed in the [Ingredients] section.
- Storage Conditions: Store at 2°C~8°C protected from light.
- Manufacturer: Roche Registration GmbH

​**​(4) Durvalumab​**​

- Brand Name: Imfinzi
- Generic Name: Durvalumab Injection
- Active Ingredient: Durvalumab, a humanized monoclonal antibody (IgG1κ type) against programmed death receptor-ligand 1 (PD-L1), expressed using Chinese Hamster Ovary (CHO) cells.
- Excipients: L-Histidine, L-Histidine hydrochloride monohydrate, α,α-Trehalose dihydrate, Polysorbate 80, Water for Injection
- Usage and Dosage: The recommended dosage is 10 mg/kg administered as an intravenous infusion over 60 minutes every 2 weeks until disease progression or intolerable toxicity.
- Adverse Reactions: Immune-mediated pneumonitis, immune-mediated hepatitis, immune-mediated colitis, etc.
- Contraindications: Hypersensitivity to the active ingredient or excipients listed in the [Ingredients] section.
- Storage Conditions: Store at 2~8°C protected from light. Do not freeze. Do not shake.
- Shelf Life: 36 months
- Manufacturer: AstraZeneca UK Limited

​**​(5) Camrelizumab​**​

- Brand Name: AiRuiKa
- Generic Name: Camrelizumab for Injection
- Active Ingredient: Camrelizumab (Humanized anti-PD-1 monoclonal antibody)
- Excipients: α,α-Trehalose dihydrate, Polysorbate 20, Acetic Acid glacial, Sodium Hydroxide, Water for Injection
- Usage and Dosage: The recommended dose is 200 mg per administration, intravenously, every 2 weeks until disease progression or intolerable toxicity.
- Adverse Reactions: Rash, thyroid dysfunction, elevated transaminases, etc.
- Contraindications: Hypersensitivity to the active ingredient or any excipient listed.
- Storage Conditions: Store and transport at 2~8°C protected from light.
- Shelf Life: 24 months
- Manufacturer: Suzhou Suncadia Biopharmaceuticals Co., Ltd.

​**​(6) Sintilimab​**​

- Brand Name: Tyvyt
- Generic Name: Sintilimab Injection
- Active Ingredient: Sintilimab (Recombinant fully human anti-programmed death receptor-1 monoclonal antibody)
- Excipients: Mannitol, Histidine, Disodium citrate dihydrate, Sodium Chloride, Disodium Edetate, Polysorbate 80, Citric acid monohydrate, Water for Injection
- Usage and Dosage: Administered by intravenous infusion. The recommended dosage is 200 mg every 3 weeks until disease progression or intolerable toxicity.
- Adverse Reactions: Pyrexia, anemia, increased aspartate aminotransferase (AST), increased alanine aminotransferase (ALT), fatigue, decreased white blood cell count, etc.
- Contraindications: Hypersensitivity to the active ingredient or excipients listed in the [Ingredients] section.
- Storage Conditions: Store vials refrigerated at 2~8°C in the original carton to protect from light. Avoid freezing. Avoid shaking.
- Shelf Life: 24 months
- Manufacturer: Innovent Biologics (Suzhou) Co., Ltd.

​**​(7) Toripalimab​**​

- Brand Name: TuoYi
- Generic Name: Toripalimab Injection
- Active Ingredient: Each vial contains Toripalimab 240 mg, produced by DNA recombinant technology using Chinese Hamster Ovary (CHO) cells.
- Excipients: Citric acid monohydrate, Disodium citrate dihydrate, Sodium Chloride, Mannitol, Polysorbate 80
- Usage and Dosage: The recommended dosage of Toripalimab is 3 mg/kg administered as an intravenous infusion every 2 weeks until disease progression or intolerable toxicity.
- Adverse Reactions: Anemia, increased ALT, fatigue, increased AST, rash, pyrexia, increased blood thyroid stimulating hormone, decreased white blood cell count, cough, pruritus, hypothyroidism, decreased appetite, increased blood glucose, increased blood bilirubin, etc.
- Contraindications: Hypersensitivity to the active ingredient or any excipient listed in the [Ingredients] section.
- Storage Conditions: Store at 2~8°C protected from light during storage and transportation. Do not freeze.
- Shelf Life: 24 months
- Manufacturer: Shanghai Junshi Biosciences Co., Ltd.

​**​(8) Tislelizumab​**​

- Brand Name: BaiZeAn
- Generic Name: Tislelizumab Injection
- Active Ingredient: Tislelizumab, a humanized monoclonal antibody (IgG4 variant) targeting programmed death receptor-1 (PD-1)
- Excipients: Sodium citrate dihydrate, Citric acid monohydrate, L-Histidine hydrochloride monohydrate, L-Histidine, Trehalose dihydrate, Polysorbate 20, Water for Injection
- Usage and Dosage: Administered by intravenous infusion. The recommended dosage is 200 mg every 3 weeks.
- Adverse Reactions: Fatigue, rash, hypothyroidism, increased ALT, increased AST, etc.
- Contraindications: Hypersensitivity to the active ingredient or any excipient listed in the [Ingredients] section.
- Manufacturer: BeiGene (Shanghai) Biotechnology Co., Ltd.

​**​8. Efficacy Observation Indicators and Visits​**​

​**​8.1 Basic Information​**​

(1) ​**​Target Lesion Assessment:​**​ Based on CT or MRI measurements of target lesion size. Requirements: lymph node short axis ≥15mm; non-lymph node lesions long axis ≥10mm.
* ​**​CR (Complete Response):​**​ Disappearance of all target lesions. All pathological lymph nodes (target and non-target) must have short axis reduced to <10mm.
* ​**​PR (Partial Response):​**​ Sum of diameters of all target lesions reduced by ≥30% relative to baseline sum.
* ​**​PD (Progressive Disease):​**​ Sum of diameters of all target lesions increased by ≥20% relative to the smallest sum recorded during the study (nadir), *and* an absolute increase of ≥5mm.
* ​**​SD (Stable Disease):​**​ Neither sufficient shrinkage to qualify for PR nor sufficient increase to qualify for PD (relative to nadir).
(2) ​**​Non-Target Lesion Assessment:​**​
* ​**​CR:​**​ Disappearance of all non-target lesions and normalization of tumor marker levels. All lymph nodes non-pathological (short axis <10mm).
* ​**​PD:​**​ Unequivocal progression of existing non-target lesions. Appearance of one or more new lesions is also considered PD.
* ​**​Non-CR/Non-PD:​**​ Persistence of one or more non-target lesions and/or tumor marker levels above normal limits, without meeting PD criteria.
(3) ​**​New Lesions:​**​ Appearance of any unequivocal new lesion(s) constitutes PD for overall assessment.

​**​8.2 Data Collection and Efficacy Assessment Time Points​**​

Efficacy assessment is generally performed every 6 weeks (one assessment cycle). Patients typically receive 2-3 cycles of drug therapy per assessment period depending on the drug.
* During the first 4 assessment cycles (24 weeks): Data collection and follow-up performed every assessment cycle (6 weeks).
* During ongoing treatment after 24 weeks: Data collection and follow-up performed every 2 assessment cycles (12 weeks).
* After disease progression or discontinuation of immune checkpoint inhibitor therapy: Data collection and follow-up performed every 12 weeks.

​**​9. Safety Evaluation and Adverse Event Management​**​

Safety evaluation includes recording and assessment of clinical symptoms, signs, laboratory tests, and all adverse events (AEs) and serious adverse events (SAEs).

​**​9.1 Definition of Adverse Event (AE)​**​

An AE is defined as any untoward medical occurrence in a clinical trial subject, from the time of signing the informed consent form until 90 days after the last administration of the study drug, regardless of whether it has a causal relationship with the study drug. AEs include, but are not limited to, the following situations:

- Worsening of a pre-existing medical condition/disease (including worsening of symptoms, signs, laboratory abnormalities);
- Occurrence of any new adverse medical condition (including symptoms, signs, newly diagnosed diseases);
- Abnormal, clinically significant laboratory value(s) or result(s).

​**​9.2 Definition of Serious Adverse Event (SAE)​**​

A serious adverse event is an adverse event that meets at least one of the following criteria:

- ​**​Results in death.​**​ *Note:* Death due to progression of the disease under study is excluded.
- ​**​Is life-threatening.​**​ ("Life-threatening" in the definition means the subject was at risk of death at the time of the event; it does not refer to an event which hypothetically might have caused death if it were more severe).
- ​**​Requires inpatient hospitalization or prolongation of existing hospitalization.​**​ Excludes the following:
  - Rehabilitation facilities
  - Nursing homes
  - Routine admission to an emergency room
  - Same-day surgery (e.g., outpatient/day/ambulatory surgery)
  - Hospitalization or prolongation of hospitalization unrelated to AE worsening is not an SAE. For example: admission for a pre-existing disease without new AEs or worsening of the pre-existing disease (e.g., for investigation of persistent laboratory abnormalities present since before the trial); admission for administrative reasons (e.g., annual routine check-up); hospitalization mandated by the trial protocol (e.g., for protocol-required procedures); elective admission unrelated to AE worsening (e.g., elective surgery - such treatments or surgeries should be recorded in the overall trial protocol and/or subject baseline data); admission solely for blood product administration.
- ​**​Results in persistent or significant disability/incapacity.​**​ (Severely interferes with the ability to conduct normal life functions).
- ​**​Results in congenital anomaly/birth defect.​**​
- ​**​Other important medical events:​**​ Defined as events that may jeopardize the subject or may require medical or surgical intervention to prevent one of the outcomes listed above.

​**​9.3 Adverse Event Assessment​**​

The Investigator will assess all AEs according to the National Cancer Institute (NCI) Common Terminology Criteria for Adverse Events (CTCAE) Version 5.0. Any AE that changes the CTCAE grade must be recorded on the AE Case Report Form (CRF)/worksheet.

All AEs, regardless of CTCAE grade, must be assessed for whether they are SAEs. Specific details can be referenced in the table below:

​**​Adverse Event Assessment Guidelines​**​

| **Aspect** | **Detail** |
| --- | --- |
| ​**​CTCAE Grade​**​ | ​**​1 (Mild):​**​ Asymptomatic or mild symptoms; clinical or diagnostic observations only; intervention not indicated. |
|  | ​**​2 (Moderate):​**​ Minimal, local, or noninvasive intervention indicated; limiting age-appropriate instrumental Activities of Daily Living (ADL). |
|  | ​**​3 (Severe or Medically Significant):​**​ Severe or medically significant but not immediately life-threatening; hospitalization or prolongation of hospitalization indicated; disabling; limiting self-care ADL. |
|  | ​**​4 (Life-threatening consequences):​**​ Urgent intervention indicated. |
|  | ​**​5 (Death related to AE):​**​ Death. |
| ​**​Seriousness (SAE)​**​ | An SAE is any AE occurring at any dose or during any use of the study drug that meets any of the following: |
|  | • Results in death; |
|  | • Is life-threatening: or in the Investigator's opinion, places the subject at immediate risk of death (Note: This does not include AEs that hypothetically might cause death if more severe); |
|  | • Results in persistent or significant disability/incapacity; (severely interferes with normal life functions); |
|  | • Requires inpatient hospitalization or prolongation of existing hospitalization; (Hospitalization defined as an inpatient admission, regardless of length, even if only for preventive observation. *Note:* Hospitalization for a pre-existing disease not worsened during the study [including elective surgery] does not constitute an SAE. Pre-existing disease refers to a clinical condition diagnosed before study drug use, recorded in the subject's history); |
|  | • Results in congenital anomaly/birth defect; (in offspring of a subject exposed to the product, regardless of time of diagnosis); |
|  | • Other important medical events: Events that may not result in death, be life-threatening, or require hospitalization, but based on appropriate medical judgment, may jeopardize the subject and may require medical or surgical intervention to prevent one of the outcomes listed above. |
| ​**​Duration​**​ | Record the start and end dates of the AE. If less than 1 day, note the appropriate length of time and unit. |
| ​**​Action Taken​**​ | Did the AE lead to discontinuation of the study drug? |
| ​**​Relationship to Study Drug​**​ | Was the AE caused by the study drug? A medically qualified Investigator must provide the causal assessment between the study drug and the AE. The Investigator must sign/date (initials) the source document or worksheet supporting the causality assessment on the AE form to ensure a medically qualified assessment has been performed. This signed document must be retained within the required regulatory time frame. The criteria below are reference guidelines to assist the Investigator in assessing the relationship based on available information. The following elements are used to assess the relationship; the greater the number and/or strength of correlation between the items and their corresponding elements, the more likely the AE was caused by the study drug: |
|  | ​**​Exposure:​**​ Is there evidence the subject was actually exposed to the study drug (e.g., credible past medical history, acceptable compliance assessment [pill count, logs, etc.], expected pharmacological effect, measurement of drug/metabolites in specimens collected in vivo)? |
|  | ​**​Timing:​**​ Is there a reasonable temporal sequence between AE occurrence and study drug administration? Does the timing fit a drug-induced AE? |
|  | ​**​Possible Causes:​**​ Can the AE *not* be explained by other etiologies, such as underlying disease, other drugs/vaccines, or other host or environmental factors? |
|  | ​**​Dechallenge:​**​ Was the study drug discontinued or dose/exposure/frequency reduced? If yes, did the AE resolve or improve? If yes, dechallenge is positive. If no, dechallenge is negative. *Note:* This criterion is not applicable if: (1) The AE led to death or permanent disability; (2) The AE resolved/improved despite continuing the study drug; (3) The trial involves a single-dose drug; (4) The study drug was administered only once. |
|  | ​**​Rechallenge:​**​ Was the subject re-exposed to the study drug in this trial? If yes, did the AE recur or worsen? If yes, rechallenge is positive. If no, rechallenge is negative. *Note:* This criterion is not applicable if: (1) The initial AE led to death or permanent disability; or (2) The trial is a single-dose trial; or (3) The study drug was administered only once. *Note:* If rechallenge for a serious and potentially study drug-related AE is planned, or if re-exposure may pose a serious potential risk to the subject/patient, rechallenge is not recommended unless continuation of the drug is considered beneficial to the patient and no alternative treatment is available, and must be approved in advance by the Sponsor. |
|  | ​**​Consistency with Study Drug Characteristics:​**​ Are the clinical/pathological manifestations of the AE consistent with prior treatment information regarding the study drug or drugs of this class (pharmacology and toxicology studies)? |
| ​**​Recording Causality​**​ | The medically qualified Investigator will report the assessment of relationship on the CRF/worksheet based on his/her best clinical judgment, considering the factors above. The table below may be used for causality assessment (not all criteria need be met): |
|  | ​**​Related:​**​ Evidence of exposure to the study drug. Temporal sequence between AE occurrence and study drug administration is reasonable. The AE is more likely explained by the study drug than by other causes. |
|  | ​**​Unrelated:​**​ The subject did not receive the study drug or the temporal relationship is unreasonable or another cause is more likely to explain the AE than the study drug (also applies to subjects with drug overdose but no related AE). |

​**​9.4 Adverse Event Recording​**​

The Investigator shall record AEs or SAEs using medical terminology/concepts. Colloquial language and abbreviations should be avoided. All AEs (including SAEs) should be recorded on the eCRF Adverse Event form.

- ​**​AE Collection Period:​**​ Investigators ascertain AEs by asking subjects non-suggestive questions. Collect all AEs, including SAEs, occurring from the time of signing the informed consent form until 90 days after the last dose, whether observed by the Investigator or spontaneously reported by the subject. After 90 days post-last dose, the Investigator should report SAEs considered related to the study drug or procedure.
- ​**​AE Follow-up:​**​ Follow AEs until they resolve to baseline or Grade 0-1, or the Investigator reasonably determines follow-up is no longer necessary (e.g., unlikely to resolve or has stabilized). If an AE does not resolve, a reasonable explanation must be recorded in the eCRF. The resolution status and date of the subject's AE or SAE should be recorded in the eCRF and medical records, regardless of relationship to the study drug.
- ​**​AE Recording Content:​**​ The Investigator must completely record each AE, including diagnosis (if no diagnosis, record symptoms, signs including laboratory abnormalities), start and end date and time (if applicable), CTCAE severity grade and changes (especially for Grade 3 or higher events), whether it is an SAE, actions taken regarding the study drug, treatment given for the AE, and outcome of the event. For SAEs, the Investigator must also provide: date the AE met SAE criteria, date the Investigator became aware of the SAE, basis for classifying the event as an SAE, hospitalization date, discharge date, possible cause of death (if applicable), date of death, whether an autopsy was performed, causality assessment related to the study procedure, causality assessment related to other drugs, and other possible causes leading to the SAE. The Investigator should also provide the rationale for the causality judgment and a description of the SAE. The SAE description should include: subject number, age, gender, height, weight; indication for study drug treatment and disease stage; relevant systemic status; clinical course of SAE occurrence, development, outcome, and resolution; relevant laboratory findings related to the SAE (must provide time of test, units, and normal range); relevant past medical history, concomitant diseases and their onset/duration; relevant medication history, concomitant medications and their start date, duration, and dosage; detailed information on study drug treatment start, duration, and dosage.

Specific considerations for AE recording:

- ​**​Diagnosis, Symptoms, and Signs:​**​ If a diagnosis is established, record the diagnosis on the eCRF, not individual symptoms and signs (e.g., record "liver failure" not "jaundice, elevated transaminases, and asterixis"). If symptoms/signs cannot be definitively attributed to a diagnosis at the time of reporting, they should be recorded as separate AEs/SAEs. If symptoms/signs are determined to be caused by the diagnosis, only the diagnosis is reported; symptoms and signs are encompassed within the diagnosis. Symptoms/signs should be removed from AE records; for SAEs, a follow-up update report may be needed.
- ​**​AEs Secondary to Other Events:​**​ Generally, AEs secondary to other events (e.g., caused by or a clinical sequelae of another event) should be recorded as the primary event, unless the secondary event is severe or constitutes an SAE. However, clinically significant secondary events occurring at a different time from the primary event should be recorded as independent AEs on the eCRF. If the association between events is unclear, they should be recorded separately.
- ​**​Persisting or Recurrent AEs:​**​ A persisting AE is one that has not resolved between two assessment time points. This AE should only be recorded once on the eCRF. Record the initial severity and update to reflect the worst severity if the event worsens. A recurrent AE is one that resolved between two assessment time points but later recurred. Record each occurrence separately on the eCRF.
- ​**​Laboratory Abnormalities:​**​ Clinically significant laboratory abnormalities should be reported as AEs. It is the Investigator's responsibility to review all laboratory abnormalities and use medical judgment to determine if each should be reported as an AE.
- ​**​Death:​**​ All deaths occurring during the entire trial period, including within the 90-day follow-up period after the last dose, regardless of relationship to the study drug, must be recorded on the Death Report Form in the eCRF and promptly reported to the Sponsor. When recording a death event, if the cause is known, record the cause of death as the AE, with the outcome "Death," and report this event as an SAE. If the cause is unknown at the time of reporting, record "Death of unknown cause" on the AE form and initially report "Death of unknown cause" as an SAE, then investigate further to determine the exact cause.
- ​**​Preexisting Medical Conditions:​**​ Symptoms/signs present at trial screening should only be recorded and reported as AEs if they worsen in severity, frequency, or nature (excluding worsening of the studied disease condition). The record should reflect the change relative to the prior state, e.g., "increased frequency of headaches."
- ​**​Disease Progression:​**​ Disease progression is defined as worsening of the subject's condition due to the primary tumor targeted by the experimental treatment. The appearance of new lesions relative to the primary tumor or progression of existing lesions is considered disease progression. Expected disease progression is not reported as an AE. Death, life-threatening events, hospitalization or prolongation of hospitalization, persistent/significant disability/incapacity, congenital anomalies/birth defects, or other important medical events resulting from symptoms/signs of *expected* disease progression are ​**​not​**​ reported as SAEs for expedited reporting.
- ​**​New Anti-Tumor Therapy:​**​ Within 90 days after the last dose, if the subject starts new anti-tumor therapy, only record and report SAEs considered related to the study drug.

​**​9.5 Expedited Reporting of SAEs and Pregnancy​**​

- ​**​SAE Reporting:​**​
  - ​**​Reporting Period:​**​ SAEs occurring from signing informed consent until 90 days (inclusive) after the last dose.
  - ​**​Reporting Timeline:​**​ Upon becoming aware of an SAE, the Investigator must complete the Serious Adverse Event Report Form within 24 hours and report it to the Sponsor, Ethics Committee, and National Regulatory Authorities.
  - ​**​Sintilimab Specific:​**​ For subjects using Sintilimab, in addition to the above, the Investigator must also report within 24 hours via email to drugsafety@innoventbio.com.
  - ​**​Reporting Outside Period:​**​ If SAEs occurring outside the above period are determined to be related to the study drug, they should also be reported.
- ​**​Pregnancy:​**​
  - ​**​Background:​**​ Drugs in this class carry embryo-fetal toxicity risks. All subjects of childbearing potential must use effective contraception.
  - ​**​Pregnancy in Female Subjects:​**​ If pregnancy occurs in a female subject exposed to the drug during the clinical trial, the subject will discontinue the study. The pregnancy must be reported to the Sponsor and Innovent Biologics (drugsafety@innoventbio.com) within 24 hours of the Investigator becoming aware.
  - ​**​Pregnancy in Partner of Male Subject:​**​ If pregnancy occurs in the partner of a male subject exposed to the drug during the clinical trial, the subject may continue in the trial. The pregnancy must be reported to the Sponsor and Innovent Biologics (drugsafety@innoventbio.com) within 24 hours of the Investigator becoming aware.
  - ​**​Pregnancy Outcome Follow-up:​**​ The Investigator must monitor the pregnant subject and follow up on the pregnancy outcome until 8 weeks after the mother delivers, reporting the result to the Sponsor and Innovent Biologics.
  - ​**​Adverse Pregnancy Outcomes:​**​ If the pregnancy results in stillbirth, spontaneous miscarriage, fetal malformation (any congenital anomaly/birth defect), or therapeutic abortion for medical reasons, this is considered an SAE and must be reported according to the SAE process and timeline.
  - ​**​SAE During Pregnancy:​**​ If an SAE occurs during pregnancy, it should be reported according to the SAE reporting procedure.

​**​10. Data Recording and Management​**​

​**​10.1 Completion and Transfer of Case Report Forms (CRFs)​**​

- Investigators transcribe data from source observations into CRFs promptly, accurately, completely, and legibly.
- CRFs must be completed for every enrolled subject.
- Transfer of completed CRFs between Investigators and Data Managers must be documented with signatures. Records must be properly maintained and archived.

​**​10.2 Data Entry and Modification​**​

- Data entry and management are the responsibility of Data Managers designated by the Statistics Department.
- Data Managers develop data entry programs and manage the database.
- Data Queries (DRQs) are generated by Data Managers for any uncertainties found in the CRFs and sent to Investigators via Clinical Monitors.
- Investigators respond to DRQs promptly.
- Data Managers modify, confirm, and enter data based on Investigator responses. Further DRQs may be issued if necessary.

​**​10.3 Data Review and Database Lock​**​

- After all data queries are resolved, "clean" data is exported and provided to Statisticians.
- The Principal Investigator (PI), Sponsor, and Statisticians jointly review the data and finalize the Statistical Analysis Plan (SAP).
- After a blind review confirms the database is correct, the PI, Sponsor, and Statisticians lock the database. Locked data files are no longer modified.

​**​11. Statistical Analysis Plan​**​

​**​11.1 Datasets for Statistical Analysis​**​

- ​**​Full Analysis Set (FAS):​**​ Analyzed according to the Intention-To-Treat (ITT) principle. Includes all subjects who received at least one efficacy evaluation. Last Observation Carried Forward (LOCF) is used for subjects who did not complete the full treatment course.
- ​**​Per-Protocol Population (PPS):​**​ Subjects who fully complied with the protocol, had good adherence, and completed all required CRF sections. Efficacy is analyzed for this population.
- ​**​Safety Analysis Set (SS):​**​ Includes all subjects who received at least one cycle of study drug treatment and have safety evaluation records.

​**​11.2 Statistical Analysis Plan​**​

- Statistical analyses will be performed using SPSS 19.0 software.
- ​**​ORR:​**​ The proportion of subjects achieving CR or PR (best overall response) will be calculated along with its 95% confidence interval.
- ​**​OS and PFS:​**​ Kaplan-Meier survival curves will be used to describe OS and PFS.
  - Subjects not experiencing death or progression will be censored at their last follow-up date.
- ​**​Treatment-Related Adverse Events:​**​ The frequency and proportion of treatment-emergent adverse events will be tabulated by type and CTCAE severity grade.

​**​12. Quality Control and Assurance​**​

- Investigators must possess appropriate professional qualifications, practice licenses, expertise, and experience relevant to the trial. They must be familiar with the protocol and relevant Sponsor-provided documents/literature, and have authority over trial personnel and resources.
- Laboratories across participating hospitals must establish unified standards for laboratory testing indicators before trial initiation.
- Training will be conducted prior to the trial start to ensure researchers thoroughly understand the protocol and specific indicator requirements.

​**​13. Criteria for Discontinuing the Trial​**​

- Requirement from the National Medical Products Administration (NMPA, formerly CFDA).
- Requirement from the Ethics Committee (EC).
- Discovery of severe safety issues during the trial.

​**​14. Ethical Principles​**​

​**​14.1 Ethical Basis​**​

- The trial follows the Declaration of Helsinki and relevant Chinese regulations and norms for clinical research.

​**​14.2 GCP Guiding Principles​**​

- Designed according to Good Clinical Practice (GCP) guidelines; execution complies with GCP.

​**​14.3 Ethics Committee (EC)​**​

- The protocol, informed consent form (ICF), and other subject materials must be submitted to the Institutional Ethics Committee (IEC) for review before trial initiation.
- Written approval (dated and signed) from the EC is mandatory before starting the trial.
- Any protocol amendments (except administrative ones) require EC approval.

​**​14.4 Informed Consent Form (ICF)​**​

- ​**​**Written informed consent is required. **Before enrollment:**
  - Investigators must explain the study purpose, methods, potential risks, and discomforts to potential subjects/guardians.
  - Subjects must have adequate time to ask questions and receive satisfactory answers.
  - Subjects and Investigators sign and date two copies of the ICF (one kept by each party).
- ​**​During Study:​**​ Subjects can ask questions at any time; Investigators must provide updates.
- ​**​Major Protocol Changes:​**​ If significant changes occur during the study, subjects must re-sign an ICF.

​**​15. Data Preservation and Summary​**​

​**​15.1 Documents to be Retained by the Investigator (Originals)​**​

- Protocol and amendments (signed)
- EC approval letter
- Lab quality control certifications
- Source medical documents
- Pathology report forms (completed, signed, dated) (retain copy)
- Investigator's SAE reports to Sponsor
- Other Documents: Investigator Brochure, CRF samples, financial agreements, signed agreements (Investigator, Sponsor), Investigator CVs/relevant documents, lab normal ranges, shipment records for study product/supplies, updated Investigator Brochures, study product dispensing logs, subject identification code list, Final Study Report.

​**​15.2 Data Preservation and Summary​**​

- ​**​CRF Acceptance & Source Document Retention:​**​
  - CRFs filled by Investigators are immediately submitted to the local study lead for acceptance and safekeeping.
  - Monitors and local leads audit medical records and CRFs per Section 10 requirements.
  - CRFs and "Study Product Dispensing Logs" are sent to the Sponsor/Coordinating Center.
  - Statistics unit manages data entry, queries, and analysis.
  - PI drafts the final report; data is archived.
  - Source documents (e.g., medical records) are stored in hospital records/archive departments. Source data for all CRF entries must be traceable.
- ​**​Summary:​**​
  - Statistics unit establishes a clinical database for unified data processing.
  - Statisticians analyze data per center and perform pooled analysis.
  - Center-specific results and summaries are confirmed by each center as the basis for drafting "Clinical Study Summary Reports."
  - Signed and stamped summary reports are sent to the coordinating center (for archiving) and Sponsor.
  - Coordinating center PI is responsible for completing the "Clinical Study Final Report."

​**​16. Trial Flowchart​**​

| **​​Phase​​** | **​​Baseline Screening​​** | **​​Each Assessment Cycle​​** | **​​Post-Progression Follow-up​​** |
| --- | --- | --- | --- |
| ​**​Activity​**​ |  |  |  |
| Inclusion/Exclusion Criteria | X |  |  |
| Demographics/PMH/Prior Tx | X |  |  |
| Vital Signs | X | X |  |
| Weight/Height | X | X |  |
| History, Physical Exam | X | X |  |
| ECOG PS Score | X | X | X |
| Blood Routine | X | X | X |
| Coagulation Function | X | X | X |
| Blood Biochemistry | X | X | X |
| Thyroid Function | X | X | X |
| Urinalysis | X | X | X |
| Viral Serology (HIV/HBV/HCV) | X |  |  |
| 12-Lead ECG | X | X |  |
| Cardiac Ultrasound | X | X |  |
| Adverse Event Assessment |  | X | X |
| Concomitant Medications | X | X | X |
| Subsequent Anti-Tumor Tx |  |  | X |
| Phone Follow-up (if no clinic) |  |  | X |
| ​**​Efficacy Assessment​**​ |  |  |  |
| Imaging Studies | X | X | X |
| Gene Mutation Testing | X |  |  |

**Attachment 1: Patient General Condition Scoring Standard (Karnofsky Performance Status - KPS)​**​

**
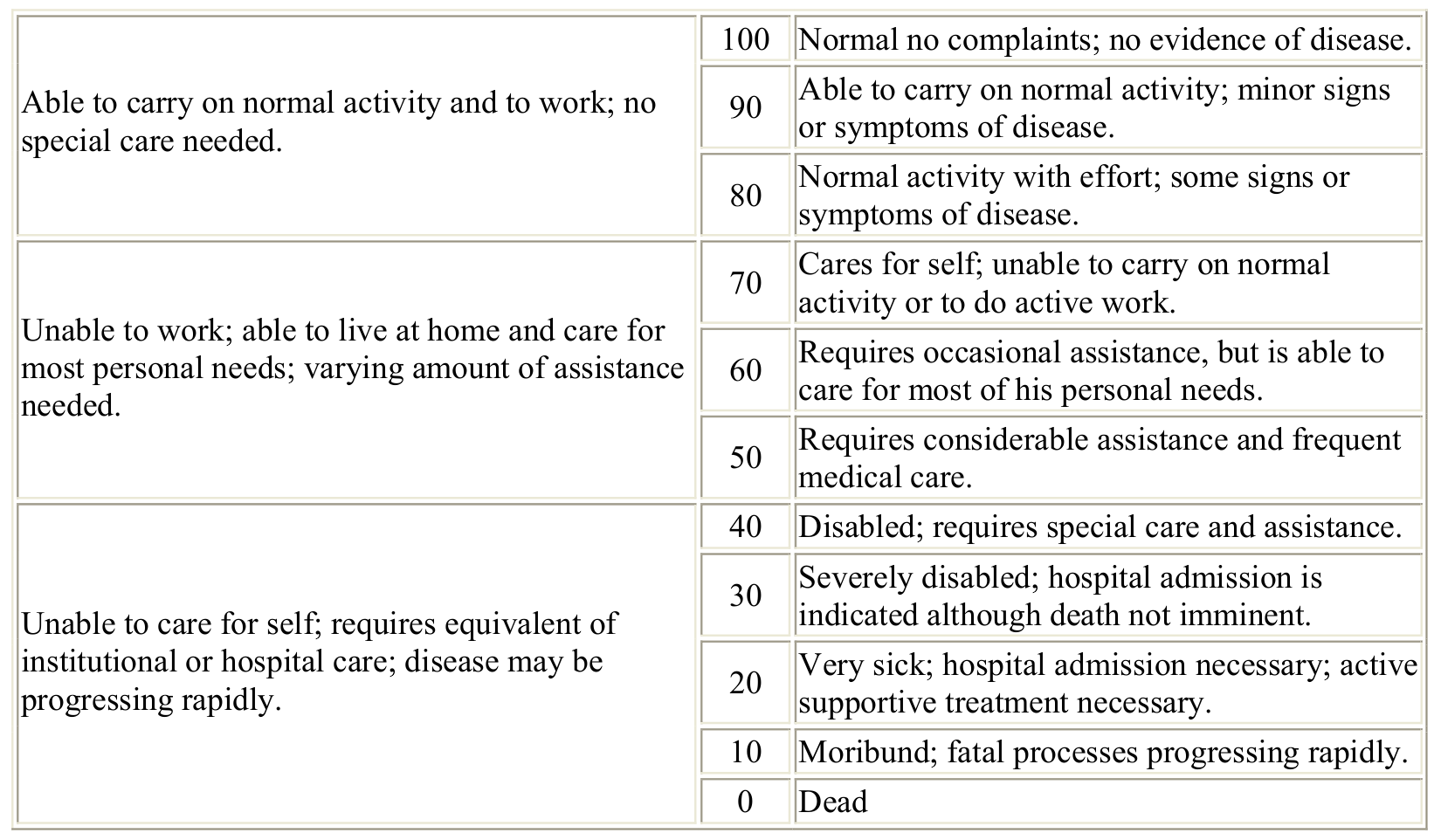
**

​**​Attachment 2: Response Evaluation Criteria in Solid Tumors (RECIST) Version 1.1​**​

3. Measurability of tumour at baseline

3.1. Definitions

At baseline, tumour lesions/lymph nodes will be categorised measurable or non-measurable as follows:

3.1.1. Measurable

*Tumour lesions*: Must be accurately measured in at least one dimension (*longest* diameter in the plane of measurement is to be recorded) with a *minimum* size of:

- 10 mm by CT scan (CT scan slice thickness no greater than 5 mm; see [Appendix II](https://www.sciencedirect.com/science/article/pii/S0959804908008733?via%3Dihub#app2) on imaging guidance).
- 10 mm caliper measurement by clinical exam (lesions which cannot be accurately measured with calipers should be recorded as non-measurable).
- 20 mm by chest X-ray.

*Malignant lymph nodes*: To be considered pathologically enlarged *and* measurable, a lymph node must be ⩾15 mm in *short* axis when assessed by CT scan (CT scan slice thickness recommended to be no greater than 5 mm). At baseline and in follow-up, only the *short* axis will be measured and followed (see Schwartz et al. in this Special Issue^[15](https://www.sciencedirect.com/science/article/pii/S0959804908008733?via%3Dihub#bib15)^). See also notes below on ‘Baseline documentation of target and non-target lesions’ for information on lymph node measurement.

3.1.2. Non-measurable

All other lesions, including small lesions (longest diameter <10 mm or pathological lymph nodes with ⩾10 to <15 mm short axis) as well as truly non-measurable lesions. Lesions considered truly non-measurable include: leptomeningeal disease, ascites, pleural or pericardial effusion, inflammatory breast disease, lymphangitic involvement of skin or lung, abdominal masses/abdominal organomegaly identified by physical exam that is not measurable by reproducible imaging techniques.

3.1.3. Special considerations regarding lesion measurability

Bone lesions, cystic lesions, and lesions previously treated with local therapy require particular comment:

**Bone lesions:**

- Bone scan, PET scan or plain films are not considered adequate imaging techniques to measure bone lesions. However, these techniques can be used to confirm the presence or disappearance of bone lesions.
- Lytic bone lesions or mixed lytic-blastic lesions, with *identifiable* *soft* *tissue* *components*, that can be evaluated by cross sectional imaging techniques such as CT or MRI can be considered as measurable lesions if the *soft* *tissue* *component* meets the definition of measurability described above.
- Blastic bone lesions are non-measurable.

**Cystic lesions:**

- Lesions that meet the criteria for radiographically defined simple cysts should not be considered as malignant lesions (neither measurable nor non-measurable) since they are, by definition, simple cysts.
- ‘Cystic lesions’ thought to represent cystic metastases can be considered as measurable lesions, if they meet the definition of measurability described above. However, if non-cystic lesions are present in the same patient, these are preferred for selection as target lesions.

**Lesions with prior local treatment:**

- Tumour lesions situated in a previously irradiated area, or in an area subjected to other loco-regional therapy, are usually not considered measurable unless there has been demonstrated progression in the lesion. Study protocols should detail the conditions under which such lesions would be considered measurable.

3.2. Specifications by methods of measurements

3.2.1. Measurement of lesions

All measurements should be recorded in metric notation, using calipers if clinically assessed. All baseline evaluations should be performed as close as possible to the treatment start and never more than 4 weeks before the beginning of the treatment.

3.2.2. Method of assessment

The same method of assessment and the same technique should be used to characterise each identified and reported lesion at baseline and during follow-up. Imaging based evaluation should always be done rather than clinical examination unless the lesion(s) being followed cannot be imaged but are assessable by clinical exam.*Clinical lesions:* Clinical lesions will only be considered measurable when they are superficial and ⩾10 mm diameter as assessed using calipers (e.g. skin nodules). For the case of skin lesions, documentation by colour photography including a ruler to estimate the size of the lesion is suggested. As noted above, when lesions can be evaluated by both clinical exam and imaging, imaging evaluation should be undertaken since it is more objective and may also be reviewed at the end of the study.*Chest X-ray:* Chest CT is preferred over chest X-ray, particularly when progression is an important endpoint, since CT is more sensitive than X-ray, particularly in identifying new lesions. However, lesions on chest X-ray may be considered measurable if they are clearly defined and surrounded by aerated lung. See [Appendix II](https://www.sciencedirect.com/science/article/pii/S0959804908008733?via%3Dihub#app2) for more details.*CT, MRI:* CT is the best currently available and reproducible method to measure lesions selected for response assessment. This guideline has defined measurability of lesions on CT scan based on the assumption that CT slice thickness is 5 mm or less. As is described in [Appendix II](https://www.sciencedirect.com/science/article/pii/S0959804908008733?via%3Dihub#app2), when CT scans have slice thickness greater than 5 mm, the minimum size for a measurable lesion should be twice the slice thickness. MRI is also acceptable in certain situations (e.g. for body scans). More details concerning the use of both CT and MRI for assessment of objective tumour response evaluation are provided in [Appendix II](https://www.sciencedirect.com/science/article/pii/S0959804908008733?via%3Dihub#app2).*Ultrasound:* Ultrasound is not useful in assessment of lesion size and should not be used as a method of measurement. Ultrasound examinations cannot be reproduced in their entirety for independent review at a later date and, because they are operator dependent, it cannot be guaranteed that the same technique and measurements will be taken from one assessment to the next (described in greater detail in [Appendix II](https://www.sciencedirect.com/science/article/pii/S0959804908008733?via%3Dihub#app2)). If new lesions are identified by ultrasound in the course of the study, confirmation by CT or MRI is advised. If there is concern about radiation exposure at CT, MRI may be used instead of CT in selected instances.*Endoscopy, laparoscopy:* The utilisation of these techniques for objective tumour evaluation is not advised. However, they can be useful to confirm complete pathological response when biopsies are obtained or to determine relapse in trials where recurrence following complete response or surgical resection is an endpoint.*Tumour markers:* Tumour markers *alone* cannot be used to assess *objective* tumour response. If markers are initially above the upper normal limit, however, they must normalise for a patient to be considered in complete response. Because tumour markers are disease specific, instructions for their measurement should be incorporated into protocols on a disease specific basis. Specific guidelines for both CA-125 response (in recurrent ovarian cancer) and PSA response (in recurrent prostate cancer), have been published.[16](https://www.sciencedirect.com/science/article/pii/S0959804908008733?via%3Dihub#bib16), [17](https://www.sciencedirect.com/science/article/pii/S0959804908008733?via%3Dihub#bib17), [18](https://www.sciencedirect.com/science/article/pii/S0959804908008733?via%3Dihub#bib18) In addition, the Gynecologic Cancer Intergroup has developed CA125 progression criteria which are to be integrated with objective tumour assessment for use in first-line trials in ovarian cancer.^[19](https://www.sciencedirect.com/science/article/pii/S0959804908008733?via%3Dihub#bib19)^*Cytology, histology:* These techniques can be used to differentiate between PR and CR in rare cases if required by protocol (for example, residual lesions in tumour types such as germ cell tumours, where known residual benign tumours can remain). When effusions are known to be a potential adverse effect of treatment (e.g. with certain taxane compounds or angiogenesis inhibitor), the cytological confirmation of the neoplastic origin of any effusion that appears or worsens during treatment can be considered if the measurable tumour has met criteria for response or stable disease in order to differentiate between response (or stable disease) and progressive disease.

4. Tumour response evaluation

4.1. Assessment of overall tumour burden and measurable disease

To assess objective response or future progression, it is necessary to estimate the *overall* *tumour* *burden* *at* *baseline* and use this as a comparator for subsequent measurements. Only patients with measurable disease at baseline should be included in protocols where objective tumour response is the primary endpoint. Measurable disease is defined by the presence of at least one measurable lesion (as detailed above in Section [3](https://www.sciencedirect.com/science/article/pii/S0959804908008733?via%3Dihub#sec1)). In studies where the primary endpoint is tumour progression (either time to progression or proportion with progression at a fixed date), the protocol must specify if entry is restricted to those with measurable disease or whether patients having non-measurable disease only are also eligible.

4.2. Baseline documentation of ‘target’ and ‘non-target’ lesions

When more than one measurable lesion is present at baseline all lesions up to a maximum of five lesions total (and a maximum of two lesions per organ) representative of all involved organs should be identified as *target* *lesions* and will be recorded and measured at baseline (this means in instances where patients have only one or two organ sites involved a *maximum* of two and four lesions respectively will be recorded). For evidence to support the selection of only five target lesions, see analyses on a large prospective database in the article by Bogaerts et al.^[10](https://www.sciencedirect.com/science/article/pii/S0959804908008733?via%3Dihub#bib10)^.

Target lesions should be selected on the basis of their size (lesions with the longest diameter), be representative of all involved organs, but in addition should be those that lend themselves to *reproducible* *repeated* *measurements*. It may be the case that, on occasion, the largest lesion does not lend itself to reproducible measurement in which circumstance the next largest lesion which can be measured reproducibly should be selected. To illustrate this point see the example in Fig. 3 of [Appendix II](https://www.sciencedirect.com/science/article/pii/S0959804908008733?via%3Dihub#app2).

*Lymph nodes* merit special mention since they are normal anatomical structures which may be visible by imaging even if not involved by tumour. As noted in Section [3](https://www.sciencedirect.com/science/article/pii/S0959804908008733?via%3Dihub#sec1), pathological nodes which are defined as measurable and may be identified as target lesions must meet the criterion of a short axis of ⩾15 mm by CT scan. Only the *short* axis of these nodes will contribute to the baseline sum. The short axis of the node is the diameter normally used by radiologists to judge if a node is involved by solid tumour. Nodal size is normally reported as two dimensions in the plane in which the image is obtained (for CT scan this is almost always the axial plane; for MRI the plane of acquisition may be axial, saggital or coronal). The smaller of these measures is the short axis. For example, an abdominal node which is reported as being 20 mm × 30 mm has a short axis of 20 mm and qualifies as a malignant, measurable node. In this example, 20 mm should be recorded as the node measurement (See also the example in Fig. 4 in [Appendix II](https://www.sciencedirect.com/science/article/pii/S0959804908008733?via%3Dihub#app2)). All other pathological nodes (those with short axis ⩾10 mm but <15 mm) should be considered non-target lesions. Nodes that have a short axis <10 mm are considered non-pathological and should not be recorded or followed.

A *sum* *of* *the* *diameters* (longest for non-nodal lesions, short axis for nodal lesions) for all target lesions will be calculated and reported as the *baseline* *sum* *diameters*. If lymph nodes are to be included in the sum, then as noted above, only the *short* axis is added into the sum. The baseline sum diameters will be used as reference to further characterise any objective tumour regression in the measurable dimension of the disease.

All other lesions (or sites of disease) including pathological lymph nodes should be identified as *non-target* *lesions* and should also be recorded at baseline. Measurements are not required and these lesions should be followed as ‘present’, ‘absent’, or in rare cases ‘unequivocal progression’ (more details to follow). In addition, it is possible to record multiple non-target lesions involving the same organ as a single item on the case record form (e.g. ‘multiple enlarged pelvic lymph nodes’ or ‘multiple liver metastases’).

4.3. Response criteria

This section provides the definitions of the criteria used to determine objective tumour response for target lesions.

4.3.1. Evaluation of target lesions

Complete Response (CR):

Disappearance of all target lesions. Any pathological lymph nodes (whether target or non-target) must have reduction in short axis to <10 mm.

Partial Response (PR):

At least a 30% decrease in the sum of diameters of target lesions, taking as reference the baseline sum diameters.

Progressive Disease (PD):

At least a 20% increase in the sum of diameters of target lesions, taking as reference the *smallest* *sum* *on* *study* (this includes the baseline sum if that is the smallest on study). In addition to the relative increase of 20%, the sum must also demonstrate an absolute increase of at least 5 mm. (*Note:* the appearance of one or more new lesions is also considered progression).

Stable Disease (SD):

Neither sufficient shrinkage to qualify for PR nor sufficient increase to qualify for PD, taking as reference the smallest sum diameters while on study.

4.3.2. Special notes on the assessment of target lesions

**Lymph nodes**

Lymph nodes identified as target lesions should always have the actual short axis measurement recorded (measured in the same anatomical plane as the baseline examination), even if the nodes regress to below 10 mm on study. This means that when lymph nodes are included as target lesions, the ‘sum’ of lesions may not be zero even if complete response criteria are met, since a normal lymph node is defined as having a short axis of <10 mm. Case report forms or other data collection methods may therefore be designed to have target nodal lesions recorded in a separate section where, in order to qualify for CR, each node must achieve a short axis <10 mm. For PR, SD and PD, the actual short axis measurement of the nodes is to be included in the sum of target lesions.

**Target lesions that become ‘too small to measure’**

While on study, all lesions (nodal and non-nodal) recorded at baseline should have their actual measurements recorded at each subsequent evaluation, even when very small (e.g. 2 mm). However, sometimes lesions or lymph nodes which are recorded as target lesions at baseline become so faint on CT scan that the radiologist may not feel comfortable assigning an exact measure and may report them as being ‘too small to measure’. When this occurs it is important that a value be recorded on the case report form. If it is the opinion of the radiologist that the lesion has likely disappeared, the measurement should be recorded as 0 mm. If the lesion is believed to be present and is faintly seen but too small to measure, a default value of 5 mm should be assigned (*Note:* It is less likely that this rule will be used for lymph nodes since they usually have a definable size when normal and are frequently surrounded by fat such as in the retroperitoneum; however, if a lymph node is believed to be present and is faintly seen but too small to measure, a default value of 5 mm should be assigned in this circumstance as well). This default value is derived from the 5 mm CT slice thickness (but should not be changed with varying CT slice thickness). The measurement of these lesions is potentially non-reproducible, therefore providing this default value will prevent false responses or progressions based upon measurement error. To reiterate, however, if the radiologist *is* able to provide an actual measure, that should be recorded, even if it is below 5 mm.

**Lesions that split or coalesce on treatment**

As noted in [Appendix II](https://www.sciencedirect.com/science/article/pii/S0959804908008733?via%3Dihub#app2), when non-nodal lesions ‘fragment’, the longest diameters of the fragmented portions should be added together to calculate the target lesion sum. Similarly, as lesions coalesce, a plane between them may be maintained that would aid in obtaining maximal diameter measurements of each individual lesion. If the lesions have truly coalesced such that they are no longer separable, the vector of the longest diameter in this instance should be the maximal longest diameter for the ‘coalesced lesion’.

4.3.3. Evaluation of non-target lesions

This section provides the definitions of the criteria used to determine the tumour response for the group of non-target lesions. While some non-target lesions may actually be measurable, they need not be measured and instead should be assessed only *qualitatively* at the time points specified in the protocol.

Complete Response (CR):

Disappearance of all non-target lesions and normalisation of tumour marker level. All lymph nodes must be non-pathological in size (<10 mm short axis).

Non-CR/Non-PD:

Persistence of one or more non-target lesion(s) and/or maintenance of tumour marker level above the normal limits.

Progressive Disease (PD):

*Unequivocal* *progression* (see comments below) of existing non-target lesions. (*Note:* the appearance of one or more new lesions is also considered progression).

4.3.4. Special notes on assessment of progression of non-target disease

The concept of progression of non-target disease requires additional explanation as follows:

**When the patient also has measurable disease**

In this setting, to achieve ‘unequivocal progression’ on the basis of the non-target disease, there must be an overall level of substantial worsening in non-target disease such that, even in presence of SD or PR in target disease, the overall tumour burden has increased sufficiently to merit discontinuation of therapy (see examples in [Appendix II](https://www.sciencedirect.com/science/article/pii/S0959804908008733?via%3Dihub#app2) and further details below). A modest ‘increase’ in the size of one or more non-target lesions is usually not sufficient to quality for unequivocal progression status. The designation of overall progression *solely* on the basis of change in non-target disease in the face of SD or PR of target disease will therefore be extremely rare.

**When the patient has only non-measurable disease**

This circumstance arises in some phase III trials when it is not a criterion of study entry to have measurable disease. The same general concepts apply here as noted above, however, in this instance there is no measurable disease assessment to factor into the interpretation of an increase in non-measurable disease burden. Because worsening in non-target disease cannot be easily quantified (by definition: if all lesions are truly non-measurable) a useful trial that can be applied when assessing patients for unequivocal progression is to consider if the increase in overall disease burden based on the change in non-measurable disease is comparable in magnitude to the increase that would be required to declare PD for measurable disease: i.e. an increase in tumour burden representing an additional 73% increase in ‘volume’ (which is equivalent to a 20% increase diameter in a measurable lesion). Examples include an increase in a pleural effusion from ‘trace’ to ‘large’, an increase in lymphangitic disease from localised to widespread, or may be described in protocols as ‘sufficient to require a change in therapy’. Some illustrative examples are shown in Figs. 5 and 6 in [Appendix II](https://www.sciencedirect.com/science/article/pii/S0959804908008733?via%3Dihub#app2). If ‘unequivocal progression’ is seen, the patient should be considered to have had overall PD at that point. While it would be ideal to have objective criteria to apply to non-measurable disease, the very nature of that disease makes it impossible to do so, therefore the increase must be substantial.

4.3.5. New lesions

The appearance of new malignant lesions denotes disease progression; therefore, some comments on detection of new lesions are important. There are no specific criteria for the identification of new radiographic lesions; however, the finding of a new lesion should be unequivocal: i.e. not attributable to differences in scanning technique, change in imaging modality or findings thought to represent something other than tumour (for example, some ‘new’ bone lesions may be simply healing or flare of pre-existing lesions). This is particularly important when the patient’s baseline lesions show partial or complete response. For example, necrosis of a liver lesion may be reported on a CT scan report as a ‘new’ cystic lesion, which it is not.

A lesion identified on a follow-up study in an anatomical location that was *not* scanned at baseline is considered a new lesion and will indicate disease progression. An example of this is the patient who has visceral disease at baseline and while on study has a CT or MRI brain ordered which reveals metastases. The patient’s brain metastases are considered to be evidence of PD even if he/she did not have brain imaging at baseline.

If a new lesion is equivocal, for example because of its small size, continued therapy and follow-up evaluation will clarify if it represents truly new disease. If repeat scans confirm there is definitely a new lesion, then progression should be declared using the date of the initial scan.

While FDG-PET response assessments need additional study, it is sometimes reasonable to incorporate the use of FDG-PET scanning to complement CT scanning in assessment of progression (particularly possible ‘new’ disease). New lesions on the basis of FDG-PET imaging can be identified according to the following algorithm:

a. Negative FDG-PET at baseline, with a positive^[l](https://www.sciencedirect.com/science/article/pii/S0959804908008733?via%3Dihub#fn1)^ FDG-PET at follow-up is a sign of PD based on a new lesion.

b. No FDG-PET at baseline and a positive FDG-PET at follow-up:

- - If the positive FDG-PET at follow-up corresponds to a new site of disease confirmed by CT, this is PD.
  - If the positive FDG-PET at follow-up is not confirmed as a new site of disease on CT, additional follow-up CT scans are needed to determine if there is truly progression occurring at that site (if so, the date of PD will be the date of the initial abnormal FDG-PET scan).
  - If the positive FDG-PET at follow-up corresponds to a pre-existing site of disease on CT that is not progressing on the basis of the anatomic images, this is not PD.

4.4. Evaluation of best overall response

The best overall response is the best response recorded from the start of the study treatment until the end of treatment taking into account any requirement for confirmation. On occasion a response may not be documented until after the end of therapy so protocols should be clear if post-treatment assessments are to be considered in determination of best overall response. Protocols must specify how any new therapy introduced before progression will affect best response designation. The patient’s best overall response assignment will depend on the findings of both target and non-target disease and will also take into consideration the appearance of new lesions. Furthermore, depending on the nature of the study and the protocol requirements, it may also require confirmatory measurement (see Section [4.6](https://www.sciencedirect.com/science/article/pii/S0959804908008733?via%3Dihub#sec2)). Specifically, in non-randomised trials where response is the primary endpoint, confirmation of PR or CR is needed to deem either one the ‘best overall response’. This is described further below.

4.4.1. Time point response

It is assumed that at each protocol specified time point, a response assessment occurs. [Table 1](https://www.sciencedirect.com/science/article/pii/S0959804908008733?via%3Dihub#tbl1) on the next page provides a summary of the overall response status calculation at each time point for patients who have measurable disease at baseline.

Table 1. Time point response: patients with target (+/– non-target) disease.

| **Target lesions** | **Non-target lesions** | **New lesions** | **Overall response** |
| --- | --- | --- | --- |
| SCR | CR | No | CR |
| CR | Non-CR/non-PD | No | PR |
| CR | Not evaluated | No | PR |
| PR | Non-PD or not all evaluated | No | PR |
| SD | Non-PD or not all evaluated | No | SD |
| Not all evaluated | Non-PD | No | NE |
| PD | Any | Yes or No | PD |
| Any | PD | Yes or No | PD |
| Any | Any | Yes | PD |

CR = complete response, PR = partial response, SD = stable disease, PD = progressive disease, and NE = inevaluable.

When patients have non-measurable (therefore non-target) disease only, [Table 2](https://www.sciencedirect.com/science/article/pii/S0959804908008733?via%3Dihub#tbl2) is to be used.

Table 2. Time point response: patients with non-target disease only.

| **Non-target lesions** | **New lesions** | **Overall response** |
| --- | --- | --- |
| CR | No | CR |
| Non-CR/non-PD | No | Non-CR/non-PD^[a](https://www.sciencedirect.com/science/article/pii/S0959804908008733?via%3Dihub#tblfn1)^ |
| Not all evaluated | No | NE |
| Unequivocal PD | Yes or No | PD |
| Any | Yes | PD |

CR = complete response, PD = progressive disease, and NE = inevaluable.

^a^ ‘Non-CR/non-PD’ is preferred over ‘stable disease’ for non-target disease since SD is increasingly used as endpoint for assessment of efficacy in some trials so to assign this category when no lesions can be measured is not advised.

4.4.2. Missing assessments and inevaluable designation

When no imaging/measurement is done at all at a particular time point, the patient is not evaluable (NE) at that time point. If only a subset of lesion measurements are made at an assessment, usually the case is also considered NE at that time point, unless a convincing argument can be made that the contribution of the individual missing lesion(s) would not change the assigned time point response. This would be most likely to happen in the case of PD. For example, if a patient had a baseline sum of 50 mm with three measured lesions and at follow-up only two lesions were assessed, but those gave a sum of 80 mm, the patient will have achieved PD status, regardless of the contribution of the missing lesion.

4.4.3. Best overall response: all time points

The *best* *overall* *response* is determined once all the data for the patient is known.

*Best response determination in trials where confirmation of complete or partial response IS NOT required*: Best response in these trials is defined as the best response across all time points (for example, a patient who has SD at first assessment, PR at second assessment, and PD on last assessment has a best overall response of PR). When SD is believed to be best response, it must also meet the protocol specified minimum time from baseline. If the minimum time is not met when SD is otherwise the best time point response, the patient’s best response depends on the subsequent assessments. For example, a patient who has SD at first assessment, PD at second and does not meet minimum duration for SD, will have a best response of PD. The same patient lost to follow-up after the first SD assessment would be considered inevaluable.

*Best response determination in trials where confirmation of complete or partial response IS required*: Complete or partial responses may be claimed only if the criteria for each are met at a subsequent time point as specified in the protocol (generally 4 weeks later). In this circumstance, the best overall response can be interpreted as in [Table 3](https://www.sciencedirect.com/science/article/pii/S0959804908008733?via%3Dihub#tbl3).

Table 3. Best overall response when confirmation of CR and PR required.

| **Overall response** | **Overall response** | **BEST overall response** |
| --- | --- | --- |
| **First time point** | **Subsequent time point** | Empty Cell |
| CR | CR | CR |
| CR | PR | SD, PD or PR^[a](https://www.sciencedirect.com/science/article/pii/S0959804908008733?via%3Dihub#tblfn2)^ |
| CR | SD | SD provided minimum criteria for SD duration met, otherwise, PD |
| CR | PD | SD provided minimum criteria for SD duration met, otherwise, PD |
| CR | NE | SD provided minimum criteria for SD duration met, otherwise NE |
| PR | CR | PR |
| PR | PR | PR |
| PR | SD | SD |
| PR | PD | SD provided minimum criteria for SD duration met, otherwise, PD |
| PR | NE | SD provided minimum criteria for SD duration met, otherwise NE |
| NE | NE | NE |

CR = complete response, PR = partial response, SD = stable disease, PD = progressive disease, and NE = inevaluable.

^a^ If a CR is *truly* met at first time point, then any disease seen at a subsequent time point, even disease meeting PR criteria relative to baseline, makes the disease PD at that point (since disease must have reappeared after CR). Best response would depend on whether minimum duration for SD was met. However, sometimes ‘CR’ may be claimed when subsequent scans suggest small lesions were likely still present and in fact the patient had PR, not CR at the first time point. Under these circumstances, the original CR should be changed to PR and the best response is PR.

4.4.4. Special notes on response assessment

When nodal disease is included in the sum of target lesions and the nodes decrease to ‘normal’ size (<10 mm), they may still have a measurement reported on scans. This measurement should be recorded even though the nodes are normal in order not to overstate progression should it be based on increase in size of the nodes. As noted earlier, this means that patients with CR may not have a total sum of ‘zero’ on the case report form (CRF).

In trials where confirmation of response is required, repeated ‘NE’ time point assessments may complicate best response determination. The analysis plan for the trial must address how missing data/assessments will be addressed in determination of response and progression. For example, in most trials it is reasonable to consider a patient with time point responses of PR-NE-PR as a confirmed response.

Patients with a global deterioration of health status requiring discontinuation of treatment without objective evidence of disease progression at that time should be reported as ‘symptomatic deterioration’. Every effort should be made to document objective progression even after discontinuation of treatment. Symptomatic deterioration is *not* a descriptor of an objective response: it is a reason for stopping study therapy. The objective response status of such patients is to be determined by evaluation of target and non-target disease as shown in [Table 1](https://www.sciencedirect.com/science/article/pii/S0959804908008733?via%3Dihub#tbl1), [Table 2](https://www.sciencedirect.com/science/article/pii/S0959804908008733?via%3Dihub#tbl2), [Table 3](https://www.sciencedirect.com/science/article/pii/S0959804908008733?via%3Dihub#tbl3).

Conditions that define ‘early progression, early death and inevaluability’ are study specific and should be clearly described in each protocol (depending on treatment duration, treatment periodicity).

In some circumstances it may be difficult to distinguish residual disease from normal tissue. When the evaluation of complete response depends upon this determination, it is recommended that the residual lesion be investigated (fine needle aspirate/biopsy) before assigning a status of complete response. FDG-PET may be used to upgrade a response to a CR in a manner similar to a biopsy in cases where a residual radiographic abnormality is thought to represent fibrosis or scarring. The use of FDG-PET in this circumstance should be prospectively described in the protocol and supported by disease specific medical literature for the indication. However, it must be acknowledged that both approaches may lead to false positive CR due to limitations of FDG-PET and biopsy resolution/sensitivity.

For equivocal findings of progression (e.g. very small and uncertain new lesions; cystic changes or necrosis in existing lesions), treatment may continue until the next scheduled assessment. If at the next scheduled assessment, progression is confirmed, the date of progression should be the earlier date when progression was suspected.

4.5. Frequency of tumour re-evaluation

Frequency of tumour re-evaluation while on treatment should be protocol specific and adapted to the type and schedule of treatment. However, in the context of phase II studies where the beneficial effect of therapy is not known, follow-up every 6–8 weeks (timed to coincide with the end of a cycle) is reasonable. Smaller or greater time intervals than these could be justified in specific regimens or circumstances. The protocol should specify which organ sites are to be evaluated at baseline (usually those most likely to be involved with metastatic disease for the tumour type under study) and how often evaluations are repeated. Normally, all target and non-target sites are evaluated at each assessment. In selected circumstances certain non-target organs may be evaluated less frequently. For example, bone scans may need to be repeated only when complete response is identified in target disease or when progression in bone is suspected.

After the end of the treatment, the need for repetitive tumour evaluations depends on whether the trial has as a goal the response rate or the time to an event (progression/death). If ‘time to an event’ (e.g. time to progression, disease-free survival, progression-free survival) is the main endpoint of the study, then routine scheduled re-evaluation of protocol specified sites of disease is warranted. In randomised comparative trials in particular, the scheduled assessments should be performed as identified on a calendar schedule (for example: every 6–8 weeks on treatment or every 3–4 months after treatment) and should not be affected by delays in therapy, drug holidays or any other events that might lead to imbalance in a treatment arm in the timing of disease assessment.

4.6. Confirmatory measurement/duration of response

4.6.1. Confirmation

In non-randomised trials where response is the primary endpoint, confirmation of PR and CR is required to ensure responses identified are not the result of measurement error. This will also permit appropriate interpretation of results in the context of historical data where response has traditionally required confirmation in such trials (see the paper by Bogaerts et al. in this Special Issue[^10^](https://www.sciencedirect.com/science/article/pii/S0959804908008733?via%3Dihub#bib10)). However, in all other circumstances, i.e. in randomised trials (phase II or III) or studies where stable disease or progression are the primary endpoints, confirmation of response is not required since it will not add value to the interpretation of trial results. However, elimination of the requirement for response confirmation may increase the importance of central review to protect against bias, in particular in studies which are not blinded.

In the case of SD, measurements must have met the SD criteria at least once after study entry at a minimum interval (in general not less than 6–8 weeks) that is defined in the study protocol.

4.6.2. Duration of overall response

The duration of overall response is measured from the time measurement criteria are first met for CR/PR (whichever is first recorded) until the first date that recurrent or progressive disease is objectively documented (taking as reference for progressive disease the smallest measurements recorded on study).

The duration of overall complete response is measured from the time measurement criteria are first met for CR until the first date that recurrent disease is objectively documented.

4.6.3. Duration of stable disease

Stable disease is measured from the start of the treatment (in randomised trials, from date of randomisation) until the criteria for progression are met, taking as reference the *smallest* *sum* *on* *study* (if the baseline sum is the smallest, this is the reference for calculation of PD).

The clinical relevance of the duration of stable disease varies in different studies and diseases. If the proportion of patients achieving stable disease for a minimum period of time is an endpoint of importance in a particular trial, the protocol should specify the minimal time interval required between two measurements for determination of stable disease.

*Note*: The duration of response and stable disease as well as the progression-free survival are influenced by the frequency of follow-up after baseline evaluation. It is not in the scope of this guideline to define a standard follow-up frequency. The frequency should take into account many parameters including disease types and stages, treatment periodicity and standard practice. However, these limitations of the precision of the measured endpoint should be taken into account if comparisons between trials are to be made.

4.7. Progression-free survival/proportion progression-free

4.7.1. Phase II trials

This guideline is focused primarily on the use of objective response endpoints for phase II trials. In some circumstances, ‘response rate’ may not be the optimal method to assess the potential anticancer activity of new agents/regimens. In such cases ‘progression-free survival’ (PFS) or the ‘proportion progression-free’ at landmark time points, might be considered appropriate alternatives to provide an initial signal of biologic effect of new agents. It is clear, however, that in an uncontrolled trial, these measures are subject to criticism since an apparently promising observation may be related to biological factors such as patient selection and not the impact of the intervention. Thus, phase II screening trials utilising these endpoints are best designed with a randomised control. Exceptions may exist where the behaviour patterns of certain cancers are so consistent (and usually consistently poor), that a non-randomised trial is justifiable (see for example van Glabbeke et al.^[20](https://www.sciencedirect.com/science/article/pii/S0959804908008733?via%3Dihub#bib20)^). However, in these cases it will be essential to document with care the basis for estimating the expected PFS or proportion progression-free in the absence of a treatment effect.

4.7.2. Phase III trials

Phase III trials in advanced cancers are increasingly designed to evaluate progression-free survival or time to progression as the primary outcome of interest. Assessment of progression is relatively straightforward if the protocol requires all patients to have measurable disease. However, restricting entry to this subset of patients is subject to criticism: it may result in a trial where the results are less likely to be generalisable if, in the disease under study, a substantial proportion of patients would be excluded. Moreover, the restriction to entry will slow recruitment to the study. Increasingly, therefore, trials allow entry of both patients with measurable disease as well as those with non-measurable disease only. In this circumstance, care must be taken to explicitly describe the findings which would qualify for progressive disease for those patients *without* measurable lesions. Furthermore, in this setting, protocols must indicate if the maximum number of recorded target lesions for those patients with measurable disease may be relaxed from five to three (based on the data found in Bogaerts et al.[^10^](https://www.sciencedirect.com/science/article/pii/S0959804908008733?via%3Dihub#bib10) and Moskowitz et al.^[11](https://www.sciencedirect.com/science/article/pii/S0959804908008733?via%3Dihub#bib11)^). As found in the ‘special notes on assessment of progression’, these guidelines offer recommendations for assessment of progression in this setting. Furthermore, if available, validated tumour marker measures of progression (as has been proposed for ovarian cancer) may be useful to integrate into the definition of progression. Centralised blinded review of imaging studies or of source imaging reports to verify ‘unequivocal progression’ may be needed if important drug development or drug approval decisions are to be based on the study outcome. Finally, as noted earlier, because the date of progression is subject to ascertainment bias, timing of investigations in study arms should be the same. The article by Dancey et al. in this special issue^[21](https://www.sciencedirect.com/science/article/pii/S0959804908008733?via%3Dihub#bib21)^ provides a more detailed discussion of the assessment of progression in randomised trials.

4.8. Independent review of response and progression

For trials where *objective* *response* (CR + PR) is the primary endpoint, and in particular where key drug development decisions are based on the observation of a minimum number of responders, it is recommended that all claimed responses be reviewed by an expert(s) independent of the study. If the study is a randomised trial, ideally reviewers should be blinded to treatment assignment. Simultaneous review of the patients’ files and radiological images is the best approach.

Independent review of progression presents some more complex issues: for example, there are statistical problems with the use of central-review-based progression time in place of investigator-based progression time due to the potential introduction of informative censoring when the former precedes the latter. An overview of these factors and other lessons learned from independent review is provided in an article by Ford et al. in this special issue.^[22](https://www.sciencedirect.com/science/article/pii/S0959804908008733?via%3Dihub#bib22)^

4.9. Reporting best response results

4.9.1. Phase II trials

When response is the primary endpoint, and thus all patients must have measurable disease to enter the trial, all patients included in the study must be accounted for in the report of the results, even if there are major protocol treatment deviations or if they are not evaluable. Each patient will be assigned one of the following categories:

1. Complete response

2. Partial response

3. Stable disease

4. Progression

5. Inevaluable for response: specify reasons (for example: early death, malignant disease; early death, toxicity; tumour assessments not repeated/incomplete; other (specify)).

Normally, all *eligible* patients should be included in the denominator for the calculation of the response rate for phase II trials (in some protocols it will be appropriate to include all treated patients). It is generally preferred that 95% two-sided confidence limits are given for the calculated response rate. Trial conclusions should be based on the response rate for all eligible (or all treated) patients and should *not* be based on a selected ‘evaluable’ subset.

4.9.2. Phase III trials

Response evaluation in phase III trials may be an indicator of the relative anti-tumour activity of the treatments evaluated and is almost always a secondary endpoint. Observed differences in response rate may not predict the clinically relevant therapeutic benefit for the population studied. If objective response is selected as a primary endpoint for a phase III study (only in circumstances where a direct relationship between objective tumour response and a clinically relevant therapeutic benefit can be unambiguously demonstrated for the population studied), the same criteria as those applying to phase II trials should be used and all patients entered should have at least one measurable lesion.

In those many cases where response is a secondary endpoint and not all trial patients have measurable disease, the method for reporting overall best response rates must be pre-specified in the protocol. In practice, response rate may be reported using either an ‘intent to treat’ analysis (all randomised patients in the denominator) or an analysis where only the subset of patients with measurable disease at baseline are included. The protocol should clearly specify how response results will be reported, including any subset analyses that are planned.

The original version of RECIST suggested that in phase III trials one could write protocols using a ‘relaxed’ interpretation of the RECIST guidelines (for example, reducing the number of lesions measured) but this should no longer be done since these revised guidelines have been amended in such a way that it is clear how these criteria should be applied for all trials in which anatomical assessment of tumour response or progression are endpoints.
