# Supplementary material for: Consolidative stereotactic radiotherapy for oligo-residual non-small cell lung cancer after first-line chemoimmunotherapy: A single-arm, phase 2 trial from China
Source: PLoS Med. 2025 Aug 1;22(8):e1004680. doi: 10.1371/journal.pmed.1004680 (PMC12316271; doi:10.1371/journal.pmed.1004680)
Supplement: S3 Protocol — (DOCX) [file pmed.1004680.s010.docx]

**巩固性立体定向放疗在一线化免治疗后寡残留非小细胞肺癌中的应用**

**负责机构:** 复旦大学附属肿瘤医院

**主要研究者:** 朱正飞

**协议编号: 2020-NSCLCSBRT**

**协议版本号: 1.0**

**方案版本日期: 2021.1.20**

**协议签署页**

**临床试验单位声明**

我将按照本协议（版本号：1.0，版本日期：2021.01.20）的要求记录临床试验数据，并确保临床试验数据真实、准确、及时、合法地包含在病例报告表中。我将负责与临床试验相关的医疗决策，以确保在试验过程中如果发生不良事件，受试者将尽快接受治疗。我了解严重不良事件的正确报告程序和要求，如果在临床研究中识别出不良事件或严重不良事件，我将按照协议要求进行报告。

复旦大学附属肿瘤医院

主要研究者（签名）：

日期（YYYY/MM/DD）：

**1.0 研究标题**

巩固性立体定向放疗在一线化免治疗后寡残留非小细胞肺癌中的应用

**2.0 背景**

肺癌是一种严重危害人类健康的恶性肿瘤。2012年全球约有180万新发肺癌病例，占所有新发癌症病例的12.9%和癌症相关死亡的19.4%[1, 2]。非小细胞肺癌（NSCLC）占所有肺癌的85%，近50%的患者在确诊时已处于晚期，治疗选择有限，预后不良[3, 4]。

PD-1/PD-L1抑制剂是晚期NSCLC治疗的重大突破。PD-1/PD-L1抑制剂单独或与化疗联合已成为无EGFR或ALK突变的晚期NSCLC的标准一线治疗方案。PD-1/PD-L1抑制剂的出现将无EGFR或ALK突变的晚期NSCLC的5年总生存率从约5%提高到约20%[5-11]。迄今为止，帕博利珠单抗（Pembrolizumab），单独或与化疗联合，已获得中国食品药品监督管理局批准用于无EGFR或ALK突变的晚期NSCLC的一线治疗。纳武利尤单抗（Nivolumab），另一种PD-1抑制剂，已获批作为无EGFR或ALK突变的晚期NSCLC的二线治疗。

随着越来越多的晚期NSCLC患者接受PD-1/PD-L1抑制剂治疗，获得性耐药逐渐成为一个问题。越来越多的证据表明，接受PD-1/PD-L1抑制剂治疗并取得客观反应的晚期NSCLC患者中有30%-40%最终会发展出获得性耐药[12, 13]。2018年6月，耶鲁癌症中心的Scott N Gettinger等人首次报告了接受PD-1/PD-L1抑制剂治疗的晚期NSCLC患者获得性耐药的临床表型。在那项研究中，包括了26例对PD-1/PD-L1抑制剂产生获得性耐药的转移性NSCLC病例，其中23例（88.0%）发展为寡进展性疾病。大多数疾病进展限于一个（54%）或两个（35%）部位，主要发生在淋巴结（包括11例仅在淋巴结发生疾病进展的患者）[14]。

寡转移状态是局部病变和广泛转移之间的过渡阶段。在这个阶段，肿瘤病灶的数量有限，进展速度较慢[1]。相应地，寡残留状态指的是在有效的系统治疗后，残留病灶分布在少数器官（≤3）和少数病灶（≤5）中的情况[2-5]。对于这些寡残留疾病患者，放疗、手术和射频消融等局部治疗可以显著延长无进展生存期（PFS）[3-5]。在靶向治疗时代，巩固性局部治疗被发现可以改善前瞻性II期ATOM研究中的PFS[2]。然而，在免疫治疗时代，对PD-1/PD-L1抑制剂的耐药仍然是实现持久疾病控制的障碍。

晚期NSCLC患者接受PD-1/PD-L1抑制剂治疗时局部治疗的最佳时机仍存在争议。耶鲁癌症中心的Scott N Gettinger等人分析了26例对PD-1/PD-L1抑制剂产生获得性耐药的患者，其中15例接受了挽救性局部治疗，其中大部分是挽救性放疗。在接受挽救性局部治疗的15例患者中，11例继续使用PD-1/PD-L1抑制剂，没有出现3-4级不良事件。这些15例患者的2年生存率达到92%，明显高于未接受挽救性局部治疗的其他患者（44%）[6]。然而，关于使用局部治疗管理PD-1/PD-L1抑制剂治疗后的寡残留疾病的报告很少。

立体定向放疗（SRT）是基于现代放疗技术平台，以较少的分割次数提供较高剂量的放疗。它对肿瘤的适形性更高，对周围组织的损伤更小，是一种安全有效的局部治疗方法。SRT在靶向治疗的寡转移晚期NSCLC治疗中的应用已被广泛报道，并逐渐成为标准治疗[7, 8]。此外，在SRT过程中，肿瘤的局部辐射剂量更高，会释放更多的肿瘤相关抗原，同时SRT能更有效地诱导抗肿瘤免疫反应，因为它更好地保护了肿瘤周围的淋巴组织。2016年，Joe Y Chang提出了iSABR的概念，主张将SRT与免疫检查点抑制剂联合用于所有阶段的实体瘤治疗[9]。此后，多项关于PD-1/PD-L1抑制剂与SRT联合治疗晚期NSCLC的回顾性和前瞻性研究结果已发表。这种联合疗法被发现是安全的，并显示出初步的疗效[10, 11]。一项关于免疫检查点抑制剂（CTLA-4抑制剂、PD-1抑制剂）与SRT联合治疗晚期NSCLC的18项代表性临床研究的荟萃分析显示，局部控制率为71%，非照射部位的反应率为41%，中位总生存时间为12.4个月[10]。2018年6月报告初步结果的Pembro-RT试验比较了SRT加帕博利珠单抗与单独帕博利珠单抗在先前治疗过的晚期NSCLC中的疗效，结果显示加用SRT可以提高反应率（41%对19%）并延长PFS（6.4对1.8个月）[12]。这些研究表明，SRT是一种安全有效的治疗方法，有潜力增加转移性NSCLC中PD-1/PD-L1抑制剂的敏感性。

**References**

1. Weichselbaum, R.R. and S. Hellman, *Oligometastases revisited.* Nat Rev Clin Oncol, 2011. **8**(6): p. 378-82.

2. Chan, O.S.H., et al., *ATOM: A phase II study to assess efficacy of preemptive local ablative therapy to residual oligometastases of NSCLC after EGFR TKI.* Lung Cancer, 2020. **142**: p. 41-46.

3. Guo, T., et al., *Pattern of Recurrence Analysis in Metastatic EGFR-Mutant NSCLC Treated with Osimertinib: Implications for Consolidative Stereotactic Body Radiation Therapy.* Int J Radiat Oncol Biol Phys, 2020. **107**(1): p. 62-71.

4. Miyawaki, T., et al., *Association between oligo-residual disease and patterns of failure during EGFR-TKI treatment in EGFR-mutated non-small cell lung cancer: a retrospective study.* BMC Cancer, 2021. **21**(1): p. 1247.

5. Zeng, Y., et al., *The value of local consolidative therapy in Osimertinib-treated non-small cell lung cancer with oligo-residual disease.* Radiat Oncol, 2020. **15**(1): p. 207.

6. Gettinger, S.N., et al., *Clinical Features and Management of Acquired Resistance to PD-1 Axis Inhibitors in 26 Patients With Advanced Non-Small Cell Lung Cancer.* J Thorac Oncol, 2018. **13**(6): p. 831-839.

7. Ning, M.S., et al., *Stereotactic ablative body radiation for oligometastatic and oligoprogressive disease.* Transl Lung Cancer Res, 2019. **8**(1): p. 97-106.

8. Basler, L., S.G. Kroeze, and M. Guckenberger, *SBRT for oligoprogressive oncogene addicted NSCLC.* Lung Cancer, 2017. **106**: p. 50-57.

9. Bernstein, M.B., et al., *Immunotherapy and stereotactic ablative radiotherapy (ISABR): a curative approach?* Nat Rev Clin Oncol, 2016. **13**(8): p. 516-24.

10. Chicas-Sett, R., et al., *Stereotactic Ablative Radiotherapy Combined with Immune Checkpoint Inhibitors Reboots the Immune Response Assisted by Immunotherapy in Metastatic Lung Cancer: A Systematic Review.* Int J Mol Sci, 2019. **20**(9).

11. Luke, J.J., et al., *Safety and Clinical Activity of Pembrolizumab and Multisite Stereotactic Body Radiotherapy in Patients With Advanced Solid Tumors.* J Clin Oncol, 2018. **36**(16): p. 1611-1618.

12. Willemijn Theelen, N.F.H.P.N.N., *Randomized phase II study of pembrolizumab after stereotactic body radiotherapy (SBRT) versus pembrolizumab alone in patients with advanced non-small cell lung cancer: The PEMBRO-RT study.* Journal of Clinical Oncology, 2018. **DOI: 10.1200/JCO.2018.36.15_suppl.9023 Journal of Clinical Oncology 36, no. 15_suppl (May 20, 2018) 9023-9023**.

**3.0 研究目的**

探索立体定向放疗（SRT）在一线化免治疗后晚期非小细胞肺癌（NSCLC）患者中的安全性、有效性及潜在疗效预测因子。

**4.0 入排标准**

**4.1 入组标准**

- 年龄≥18岁且≤75岁。
- ECOG PS 0-1。
- 通过肿瘤活检和/或细针抽吸确诊的IV期NSCLC。
- 不包括EGFR、ALK和ROS-1等驱动基因阳性。
- 根据RECIST1.1至少有一个可测量病灶。
- 患者应在一线化免治疗后有临床获益且合并颅内和/或颅外ORD。临床获益的定义为：部分反应（PR）或持久稳定疾病（SD）（SD持续时间不少于6个月）。颅内和/或颅外ORD的定义如下：颅外ORD定义为残留肿瘤限于3个器官和5个病灶，且没有基线BMs或有完全颅内反应；颅内ORD定义为BMs限于10个病灶，最大肿瘤体积<10 mL，最长直径<3 cm，总累积体积≤15 mL，对于那些有残留BMs且没有颅外进展疾病（在这种情况下，不一定需要颅外ORD）的患者。至少有一个直径>1 cm的BM病灶或在1.5 mm厚层磁共振成像（MRI）上的病灶直径>0.5 cm是反应评估的要求。
- 经研究者评估颅外和/或颅内寡残留肿瘤病灶适合接受巩固性SRT。
- 有放疗史的患者如果满足以下标准则符合条件：
  1. 放疗在研究入组前超过4周进行。
  2. 至少有一个可测量病灶在放疗区域外。
- 根据研究者的意见，没有姑息放疗的指征。
- 有手术史的患者如果从手术的毒性和/或并发症中充分恢复，则符合条件。
- 签署同意使用新鲜肿瘤活检的知情同意书。
- 育龄妇女和男性必须同意在试验期间使用有效的避孕措施。
- 预计生存时间超过3个月。
- 在入组前1周内有足够的器官功能：
  1. 足够的骨髓功能：血红蛋白≥80g/L，白细胞（WBC）计数≥4.0 * 10^9/L或中性粒细胞计数≥1.5 * 10^9/L，血小板计数≥100 * 10^9/L；
  2. 足够的肝功能：总胆红素<1.5 x正常上限（ULN）。注意：如果总胆红素>1.5 x ULN，直接胆红素必须≤ULN，天门冬氨酸氨基转移酶（AST）和丙氨酸氨基转移酶（ALT）≤2.5 ULN；
  3. 足够的肾功能：血清肌酐≤1.5 x ULN或肌酐清除率≥50 mL/min；
- 能够理解和愿意提供知情同意。

**4.2 排除标准**

- 严重的自身免疫疾病，如炎症性肠病（包括克罗恩病和溃疡性结肠炎）、类风湿性关节炎、硬皮病、系统性红斑狼疮、韦格纳肉芽肿和相关血管炎。
- 有症状的间质性肺病或临床活动性感染性/非感染性肺炎。
- 其他恶性肿瘤或同时恶性肿瘤的病史。
- 活动性感染、充血性心力衰竭或在入组前6个月内有心肌梗塞、不稳定心绞痛或心律失常的证据。
- 任何严重或无法控制的系统性疾病的证据，研究者认为这些疾病使患者不宜参加试验或可能危及遵守协议。不需要对慢性疾病进行筛查。
- 根据研究者的意见，有姑息放疗指征的患者。
- 混合小细胞与非小细胞肺癌组织学。
- 患者怀孕（如适用，通过血清β-HCG确认）或正在哺乳。
- 接受过肿瘤疫苗治疗的患者；或在治疗开始前4周内接受过活疫苗接种。注意：仅在流感季节允许接种流感疫苗，而不允许接种如FluMist等活流感疫苗。
- 接受免疫抑制剂或其他研究治疗的患者。长期皮质类固醇使用者也被排除。
- 精神障碍、药物滥用和社会条件可能影响遵从性的患者。
- 对PD-1/PD-L1抑制剂有过敏反应或禁忌症的患者。

**4.3 退出标准**

- 错误入组者。未接受研究治疗的患者应立即退出，其信息将不包括在研究分析中。如果已经开始研究治疗，是否退出的决定由研究者在评估患者的利弊后做出。如果需要排除，患者在完成治疗后随访后退出研究，患者将不包括在疗效分析中，但将包括在安全性分析中。
- 在治疗前评估阶段有以下任何条件的患者：新发系统性疾病或原有系统性疾病恶化并符合排除标准；要求分组或失访或死亡；遵从性差。
- 被研究者认为需要排除的患者，研究者应向主要研究者报告排除原因。经同意后，未接受研究治疗的患者应立即退出，其信息将不包括在研究分析中。如果患者已经开始研究治疗并在完成治疗后随访访问后退出研究，患者将不包括在疗效分析中，但将包括在安全性分析中。
- 如果受试者在试验过程中不希望继续试验，可以随时要求退出试验。
- 如果试验过程中发生严重不良事件，根据研究者的判断，应停止受试者参加试验；
- 试验恶化可能威胁生命，其他影响试验，和/或在试验观察条件下；
- 在治疗阶段失访或死亡；
- 在化疗期间共享试验、中药、其他免疫治疗、放疗增敏剂或其他影响疗效和毒性评估者；
- 在临床试验方案实施过程中发生严重偏差，使药物疗效评估变得困难。坚持索赔。

**5.0 入组前评估**

- 完整的病史和人口统计数据；
- 组织病理学诊断和临床分期；
- 与肿瘤相关的症状和体格检查；
- 驱动基因突变（组织或外周血检测，ARMS，NGS，或数字PCR）；
- 基线肿瘤评估（治疗开始前3周内全身肿瘤成像）；
- 常规检查结果（血液、尿液和粪便常规、肝肾功能、心电图等）。

1. **治疗和随访**
   1. **患者治疗**

患者将接受原始维持PD-1/PD-L1抑制剂治疗，最长可达2年或直至确认进展或不可接受的毒性。PD-1/PD-L1抑制剂将作为静脉输注给予。在有效治疗后有寡残留NSCLC的患者将接受覆盖所有残留病灶的根治性意向SRT治疗。剂量分割方案的选择由治疗放射肿瘤科医生自行决定，参考NRG-BR001研究方案。

- 1. **定期随访**

1. SRT治疗后1个月进行第一次影像检查，之后每2个月进行一次。具体检查时间应在预定时间的±5天内；
2. 影像检查的详细情况应基于患者化免治疗前的基线发现：
3. 除骨转移外，基线评估中发现的病灶部位每次检查都需要进行。
4. 对于骨转移，如果有明确的软组织肿块，应进行相应的影像检查，否则不是必须的。每次检查不是必须的。骨扫描不是必须的。
5. 对于基线评估中没有病灶的部位，当患者有相应的症状或异常生化指标时，由负责医生决定是否进行额外的影像检查。
6. 如果患者在治疗过程中有任何病灶的明确进展（根据RECIST1.1标准），则认为是全身影像。
7. 对于基线脑转移的患者，每次随访应进行脑部MRI检查，根据RANO标准评估颅内病灶的反应。
8. 根据需要检查血液常规、肝肾功能、肿瘤标志物等血液学指标。
9. 记录肿瘤相关症状和体格检查结果的变化。
   1. **疾病进展时的检查**
10. 体格检查：体重、体表面积、生命体征；
11. 肿瘤评估：肿瘤相关症状、全面影像检查；
12. 实验室检查：血液常规、尿液常规、肝肾功能、心电图（ECG）。

**7.0 样本量**

所有接受一线化免治疗的无驱动基因突变NSCLC患者均被纳入一项前瞻性观察研究，该研究已获批准并注册为NCT04766515。那些最佳反应为部分反应（PR）或持久稳定疾病（SD）（SD持续时间不少于6个月）的患者将被筛选参加本研究。由于在一线化免治疗后发展为ORD的转移性NSCLC患者中有限的生存数据，我们汇总了三项先前回顾性研究的个体患者数据，发现这一疾病人群的中位PFS为10.0个月，作为历史对照。增加巩固性SRT，疾病进展或死亡的风险将降低40%（HR=0.6）。在功率=90%和α=0.05（单侧）的条件下，这项单臂研究需要总共53个可评估对象。考虑到数据分析前10%的退出率，我们需要总共59名受试者。

此外，为进一步探索巩固性立体定向放疗的疗效，本研究拟进行一项倾向性匹配（propensity score match）分析：在本研究受试者入组的同一时期，本中心所有接受免疫联合化疗一线治疗后达到本研究规定的ORD标准、在一线治疗耐药前未接受任何局部治疗干预的患者，纳入前瞻性观察性队列，并根据常见的临床病理特征与纳入本研究的受试者进行PSM匹配，比较PSM匹配后的患者的生存差异。

1. **研究评估**
   1. **研究终点**

**8.1.1 主要终点**

**无进展生存（PFS）。**PFS从一线化免治疗开始日期测量至初始疾病进展日期，根据实体肿瘤反应评估标准（RECIST）版本1.1或死亡。在数据分析时未进展的生存者在最后随访日期被审查。

**8.1.2 次要终点**

**治疗相关不良事件（TRAEs）。**TRAEs将根据CTCAE v.5.0进行评估和分级。

**总生存（OS）。**OS从一线化免治疗开始日期测量至任何原因死亡日期。在数据分析时生存的参与者在最后随访日期被审查。

**8.1.3 探索性终点**

本研究将在特定时间点获取外周血和组织样本，并按照规格保存和处理，用于以下转化研究：

1. 基线肿瘤组织中免疫浸润细胞的分子亚群与治疗反应之间的相关性。

疗效与外周血中的细胞因子、免疫细胞亚群及其动态变化之间的相关性。

- 1. **反应评估标准**

根据实体肿瘤反应评估标准（RECIST）版本1.1（见附录3）。

**9.0 年度研究计划**

| 2021.1-2021.03 | 临床研究启动。 |
| --- | --- |
| 2021.03-2021.12 | 受试者招募和SBRT治疗（平均每月6~7名受试者）。 |
| 2022.01-2022.12 | 患者招募。定期随访。持续动态外周血样本收集。 |
| 2023.01-2023.12 | 随访完成。数据收集和分析。外周血样本检测和分析；数据和论文撰写总结。 |

**10.0 伦理和法律事项**

**10.1 独立伦理委员会（IEC）**

根据GCP、中国法律法规和相关组织的要求，所有参与研究的中心在研究开始前应获得伦理委员会批准文件，并在必要时进行修订或重新试验。

**10.2 本研究的伦理指导**

涉及本研究的操作、评估和程序文件的解决方案是确保研究人员在以下临床实践指南和赫尔辛基宣言指南中详细说明。本研究的实施还将遵循中国的相关法律法规。

研究人员未经伦理委员会和赞助商书面同意，不得修改研究计划。然而，在紧急情况下，为了消除参与者的风险因素，研究人员可以在未经伦理委员会/赞助商同意或支持的情况下偏离或更改计划。偏差或更改及其原因应尽快提交给伦理委员会/赞助商，如果适当，还应提交修改建议。研究人员必须充分解释所有偏差或更改的研究。

**10.3 参与者信息和知情同意**

研究的主要信息和知情同意书应提供给受试者。在研究开始前，研究人员必须向参与者提供经伦理委员会批准的知情同意书和所有其他书面信息。伦理委员会批准文件和经批准的受试者指南/知情同意书必须存档在研究文件中。

在本研究的任何具体步骤实施之前，必须获得受试者的签名知情同意。

**10.4 保密性**

关于患者身份的所有记录都是保密的，并且根据相关法律法规允许，这些数据不会公开。只有相关人员，如研究人员、研究护士可以知道患者的身份信息。

参与者的名字不会出现在病例报告表中。病例报告表只记录受试者编号和姓名缩写，如果受试者的姓名出现在任何其他文件中（如病理报告、影像检查），必须在文件副本中覆盖。使用计算机存储的报告必须符合当地数据保护法律。在研究结果发表时，受试者的身份将保密。

研究人员将保留一份清单，以识别受试者的记录。

**10.5 研究计划修订条件**

除非是为了消除试验对象的损害而进行的紧急调整，或者只是关于实验研究的后勤和管理调整，如仲裁员、替代者数量。所有修订计划必须提交给相应的伦理委员会，并在研究人员实施调整后获得许可。

**10.6 监管**

如果患者保密性符合当地要求，负责的仲裁员将定期联系并访问研究人员，并被允许检查各种试验记录表（案例）和其他相关数据。

在整个研究期间，负责的仲裁员负责定期检查报告，验证研究计划的合规性，并检查输入数据的完整性、一致性和准确性。仲裁员应能够访问实验室试验报告和其他特权，患者记录以验证病例报告上的输入。研究人员（或其指定人员）同意合作，以确保在检查访问期间发现的任何问题都可以解决。

**附录1. Karnofsky表现状态量表定义评分（%）标准**

**
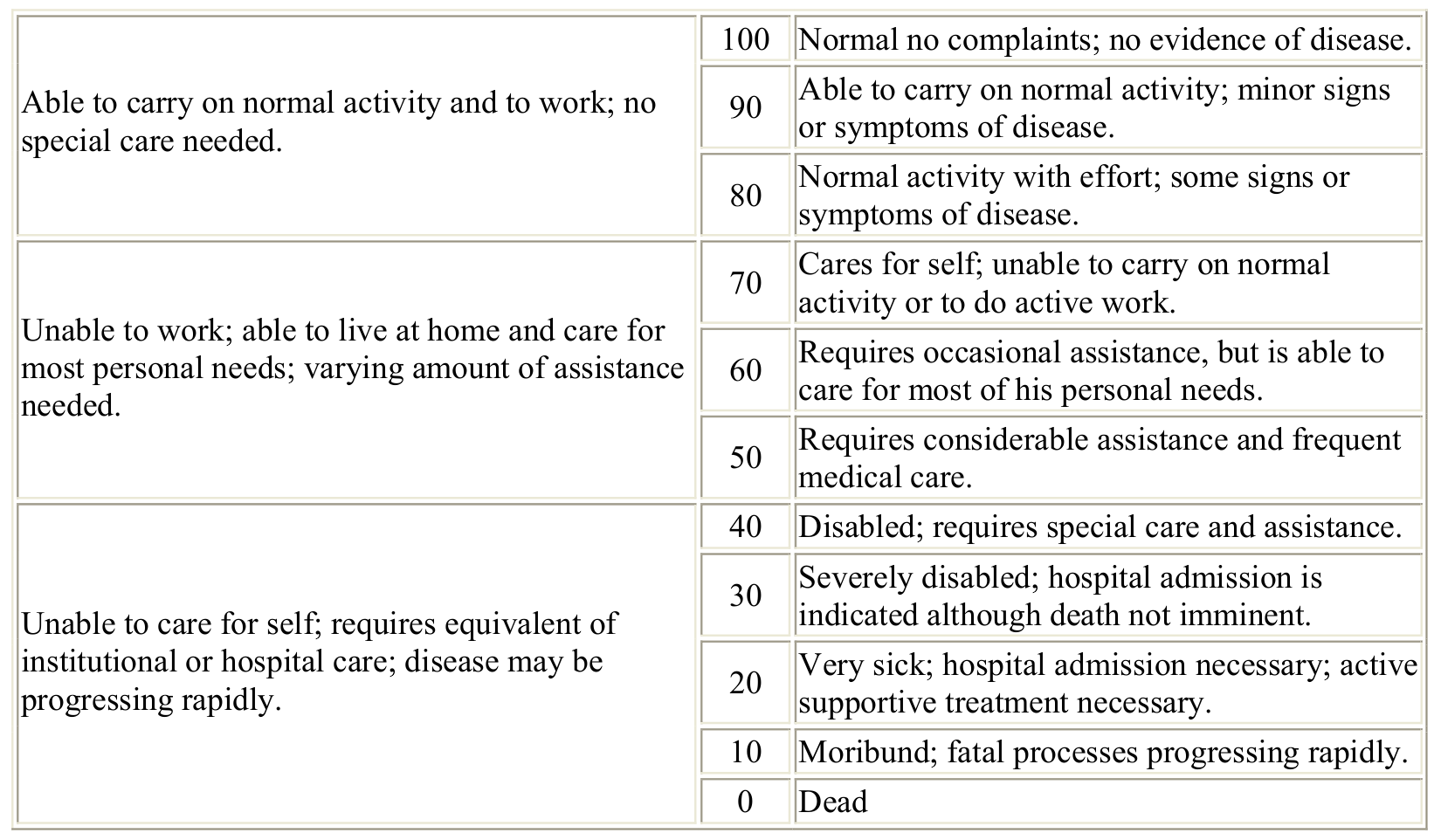
**

**附录2. 第八版肺癌分期分类**


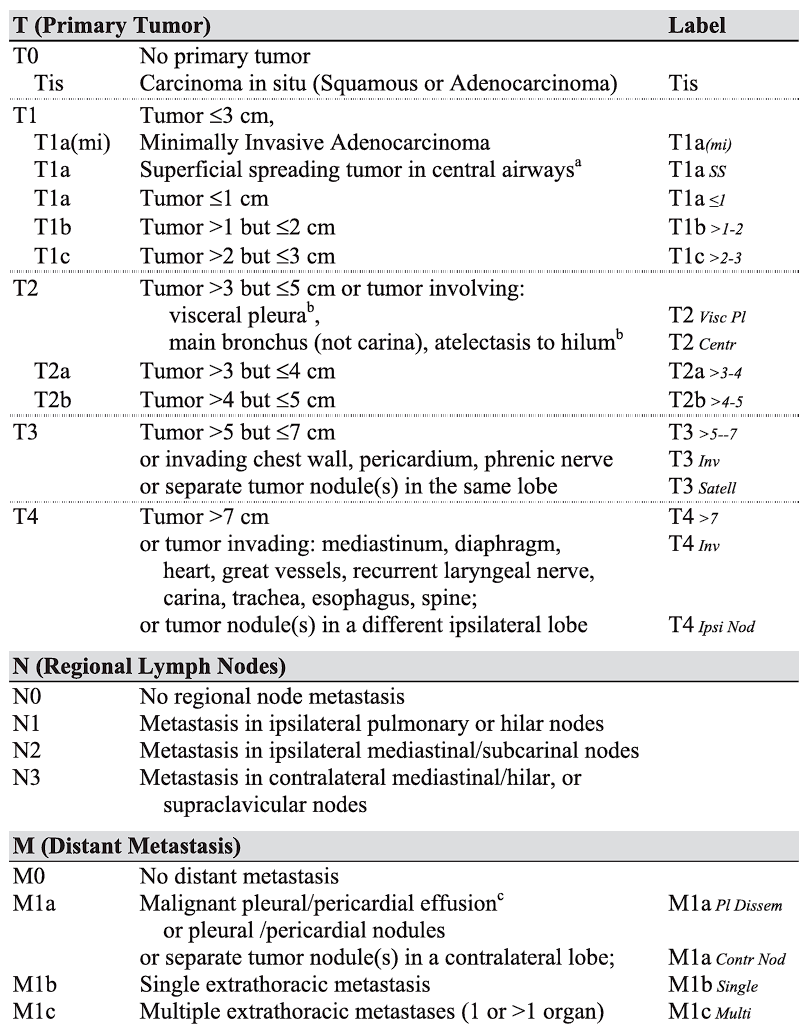


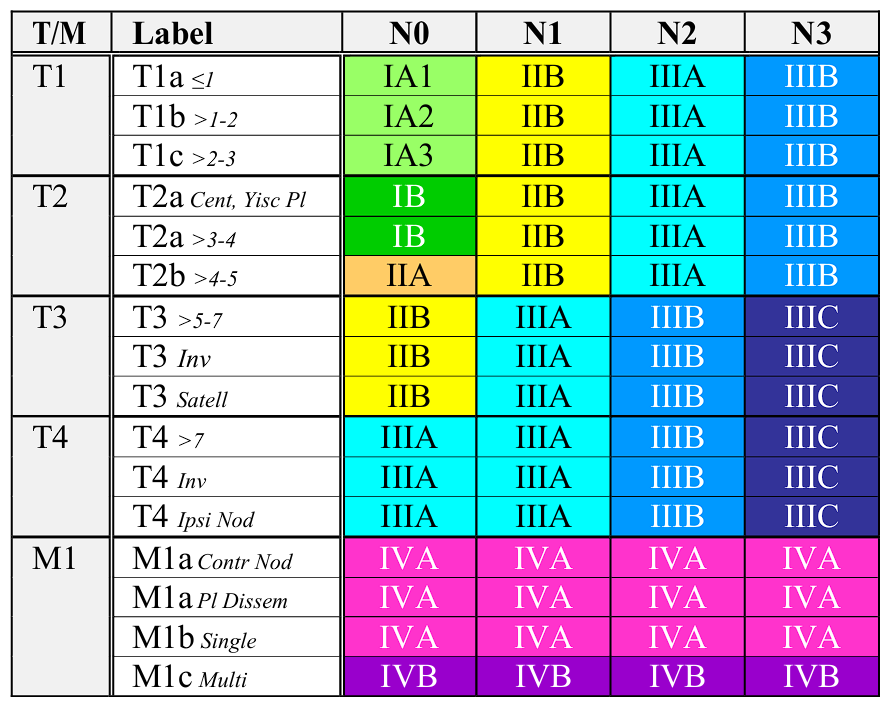


**附录3. 新实体肿瘤反应评估标准：修订版RECIST指南（版本1.1）**

**3. 基线肿瘤的可测量性**

**3.1定义**

在基线时，肿瘤病灶/淋巴结将被分类为可测量与不可测量，如下所示：

**3.1.1 可测量**

**肿瘤病灶：**

必须在至少一个维度上准确测量（测量平面中的最长直径将被记录），最小尺寸为：

- 10mm，通过CT扫描（CT扫描切片厚度不大于5mm；见附录Ⅱ关于影像学指南）。
- 10mm，通过临床检查（无法用卡尺准确测量的病灶应记录为不可测量）。
- 20mm，通过胸部X光检查。

**恶性淋巴结：**

当通过CT扫描评估时（建议CT扫描切片厚度不超过5mm），淋巴结的短轴必须≥15mm才会被认定为是病理性增大和可测量。在基线和随访中，仅测量和跟踪其短轴（见施瓦茨等人在本期特刊15）。另请参阅下文关于**“靶病灶和非靶病灶的基线记录”**的说明，以获取有关淋巴结测量的信息。

**3.1.2 不可测量**

所有其他病灶，包括小病灶（最长直径<10mm或短轴≥10至<15mm 的病理性淋巴结）以及真正不可测量的病灶。被认为真正无法测量的病灶包括：软脑膜疾病、腹水、胸腔或心包积液、炎性乳腺疾病、皮肤或肺淋巴管炎受累、通过体格检查发现但无法进行可重复的影像学技术测量的腹部肿块/腹部器官肿大。

**3.1.3 关于病灶可测量性的特殊注意事项**

骨病灶、囊性病灶和既往接受局部治疗的病灶需要特别注意：

**骨病灶：**

- 骨扫描，PET扫描或平片不认为是测量骨病变合适的影像学技术。然而，这些技术可用于确认骨病灶的存在或消失。
- 如果软组织成分符合上述可测量性的定义，则可通过横断面成像技术（如 CT或 MRI）评估的具有可识别软组织成分的溶解性骨病灶或混合溶解性原始细胞病灶，可被视为可测量病灶。
- 原始细胞性骨病灶是不可测量的。

**囊性病灶：**

- 符合影像学定义的单纯性囊肿标准的病灶不应被视为恶性病灶（既非可测量，也非不可测量），因为根据定义，它们是单纯性囊肿。
- 被认为代表囊性转移的“囊性病灶”如果符合上述可测量性的定义，则可以被视为可测量的病灶。然而，如果同一患者存在非囊性病灶，则首选这些病灶作为靶病灶。

**既往接受局部治疗的病灶：**

- 除非病灶已证实疾病进展，否则位于先前照射区域或接受其他局部区域治疗的肿瘤病灶通常不被认为是可测量的。研究方案应详细说明此类病灶被认定是可测量的条件。

**3.2 测量方法规范**

**3.2.1. 病灶的测量**

如果使用经过临床评估的卡尺，所有测量值均应以公制单位记录。所有基线评估都应尽可能接近治疗起点，切勿超过治疗起点的前4周。

**3.2.2. 评估方法**

应使用相同的评估方法和相同的技术，来描述基线和随访期间发现和报告的每种病变。除非所跟踪的病灶无法成像，但可通过临床检查进行评估，否则应始终进行基于影像学的评估，而不是临床检查。

**临床病灶**：临床病灶只有在浅表且使用卡尺评估 ≥10mm直径（例如皮肤结节）时才被认为是可测量的。皮肤表浅病灶建议使用彩色照片记录，照片附上测量病灶大小的比例尺。如前所述，当病灶既可用临床检测也可用影像学检查时，由于影像学更客观并可用于治疗后研究终点的回顾，应采用影像学检查。

**胸部X片**：胸片和胸部CT测量病灶，因为CT在发现新病灶等方面比较X片更敏感，优先选用CT扫描，特别在重要的治疗终点时。当然，肺实质中边界清楚的病灶也可使用胸片检测。详见附录Ⅱ。

**CT, MRI**：CT是目前用来评估病灶疗效最有效和重复性最好的检测方法。指南定义可测量病灶用CT扫描基于层厚不超过5mm。如附录Ⅱ所示，当CT层厚超过5mm，可测量病灶最小应是层厚的两倍。MRI在某也情况下也可使用（如全身扫描）。更多关于使用CT和MRI检测实体瘤评估疗效的意见详见附录Ⅱ。

**超声检查**：超声检查不适用于评估病灶大小，不应用作测量方法。超声检查在两次相邻的观察间不能完全再现，而且结果依赖于检查者，从一次检测到下一次，不能保证相同的技术和测量结果（详见附录Ⅱ）。如果在研究过程中通过超声发现新的病灶，建议用CT或MRI验证。如果顾虑CT的射线照射，可用MRI代替来检测待检病灶。

**内窥镜检查、腹腔镜检查**：不建议使用这些技术进行肿瘤客观评估。然而，在获得活检时，它们可用于确认完全病理性缓解；或者在完全缓解或手术切除后复发为终点的试验中确定复发。

**肿瘤标志物**：肿瘤标志物不能单独用于评估肿瘤客观疗效。但是，如果标志物最初高于正常值上限，则必须使其正常化，才能考虑患者完全缓解。由于肿瘤标志物具有疾病特异性，应根据疾病特异性将其测量说明纳入方案中。关于CA-125反应（复发性卵巢癌）和PSA反应（复发性前列腺癌）的具体指南已经发布16-18。此外，国际妇科肿瘤协作小组还制定了CA125进展标准，该标准将与肿瘤客观评估相结合，用于卵巢癌的一线试验19。

**细胞学、组织学**：如果方案要求，这些技术可用于在极少数情况下区分PR和CR（例如，生殖细胞肿瘤等肿瘤类型的残留病变，其中已知残留良性肿瘤可能仍然存在）。当已知积液是治疗的潜在不良反应时（例如使用某些紫杉烷类化合物或血管生成抑制剂），如果可测量的肿瘤符合缓解或疾病稳定标准，则可以考虑对治疗期间出现或恶化的任何积液的肿瘤起源进行细胞学确认，以区分缓解（或疾病稳定）和疾病进展。

**4. 肿瘤疗效评估 (Tumour response evaluation)**

**4.1. 评估肿瘤总体负荷与可测量病变**

为了评估客观缓解或未来进展，有必要在基线时评估肿瘤总体负荷，并将其作为后续测量的比较对象。在以肿瘤客观缓解为主要终点的方案中，只有基线有可测量病变的患者才会入组。可测量病变定义为，存在至少一个可测量的病灶（详见上文第3节）。在主要终点是肿瘤进展的研究中（进展时间或固定日期的进展比例），方案必须指定是否仅限于患有可测量病变的患者入组，或者仅患有不可测量病变的患者是否也符合入组条件。

**4.2. 靶病灶与非靶病灶的基线记录**

当基线存在一个以上的可测量病灶时，代表所属器官的病灶总共最多5个，会被认定为靶病灶(每个器官最多2个)，并在基线进行记录和测量（这意味着，患者在只有一个或两个器官部位病变的情况下，将分别记录最多两个或四个病灶）。对于仅选择5个靶病灶的依据，参见Bogaerts等人对一个大型前瞻性数据库的分析文章10。

靶病灶应基于其大小尺寸进行选择(直径最长的病灶)，应是所属器官中最有代表性的病灶，但也应该是可重复测量的病灶。有时可能会出现这样的情况，最大病灶本身不适合进行重复测量。这种情况下，应该选择下一个可重复测量的最大病灶。为了说明这一点，请参阅附录Ⅱ中的图3的例子。

淋巴结需特别说明，因为它们是正常的解剖结构，即使不涉及肿瘤，也可以通过影像看到。如第3节所述，定义为可测量且可能被确定为靶病灶的病理性淋巴结，必须符合CT扫描短径≥15 mm的标准。只有这些结节的短径，才会被纳入基线总和的计算。通常放射科医生通过使用淋巴结的短径，来判断是否有实体瘤。结节大小通常记录为所获影像的两个平面维度 (对于CT扫描，这几乎总是轴向面；对于MRI，采集平面可能是轴向、矢状或冠状)。在这些测量中，较小的那一条径线就是短径。例如，一个报告为20mm * 30mm的腹部淋巴结，其短径为20mm，属于可测量的恶性淋巴结。本例中，应记录20mm作为结节的测量值(参见附录Ⅱ中的图4示例)。其他所有病理性淋巴结(短径≥10 mm，但<15 mm)，应被认定为非靶病灶。短径<10mm的淋巴结被认为是非病理性的，不应该记录或随访。

所有靶病灶的直径之和(非淋巴结病灶使用长径，淋巴结病灶使用短径)，将会在基线时计算，并记录为基线总和。如果淋巴结被纳入总和计算中，如上所述，只有短径会算入总和。基线直径总和将作为参考，以进一步在病变可测量的维度上描述任何肿瘤客观消退。

所有其他病灶(或病变部位)，包括病理性淋巴结，应认定为非靶病灶，也应在基线时记录。这些病灶不需要测量，应随访为“存在”、“不存在”，或在极少数情况下“明确进展”（更多细节见下文）。此外，可以将所属同一器官的多个非靶标病灶记录为病例报告表上的单个项目（例如“多发性盆腔淋巴结肿大”或“多发性肝转移”）。

**4.3. 疗效标准**

本节提供了用于确定靶病灶的肿瘤客观疗效的定义标准。

**4.3.1. 靶病灶的疗效评价**

- **完全缓解（Complete Response, CR）**： 所有靶病灶消失。任何病理性淋巴结 (无论是否为靶病灶) 的短径必须缩短至 <10 mm。
- **部分缓解（Partial Response, PR）**： 以基线直径总和作为参考值，靶病灶的直径总和至少减小30%。
- **疾病进展（Progressive Disease, PD）**： 以研究中的直径总和的最小值作为参考值，靶病灶的直径总和至少增加20% (如果基线总和是研究中的最小值，则参考值为基线总和)。除了相对增加20%，总和增加的绝对值还必须至少是5毫米 (注意：出现一个或多个新病灶也被认为是疾病进展)。
- **疾病稳定（Stable Disease, SD）**： (直径总和) 既没有减少得足够多，以达到PR；也没有增加得足够多，以达到PD (以研究中的直径总和的最小值作为参考值)。

**4.3.2. 靶病灶评估的注意事项**

**淋巴结 (Lymph nodes)**

被认定为靶病灶的淋巴结，即使在研究中消退至10mm以下，应始终记录短轴的实际测量值（在与基线检查相同的解剖平面上测量）。这意味着当淋巴结作为靶病灶时，即使满足完全缓解标准，病灶的“总和”也不可能为零，因为正常淋巴结定义为拥有<10mm的短轴。因此，为了符合CR条件，病例报告表或其他数据收集方法可以将淋巴结靶病灶记录设计在单独的部分，其中每个节点的短轴必须<10mm。对于PR，SD和PD，节点短轴的实际测量值，应包含在靶病灶的总和中。

**“太小而无法测量”的靶病灶 (Target lesions that become ‘too small to measure’)**

在研究中，基线记录的所有病变（淋巴结和非淋巴结）都应在每次后续评估中记录其实际测量值，即使非常小（例如2mm）。然而，有时在基线时记录为靶病灶的病变或淋巴结在CT扫描中变得非常微弱，以至于放射科医生可能无法给定精确的测量值，而将其报告为“太小而无法测量”。

发生这种情况时，在病例报告表上记录一个值很重要。如果放射科医生认为病灶可能已经消失，则应将测量记录为0mm。如果认为病灶存在且隐约可见，但太小而无法测量，则应分配默认值5mm（注意：此规则不太适用于淋巴结，因为通常它们在正常时具有可定义的大小，并且经常被脂肪包围，例如在腹膜后；但是，如果认为淋巴结存在并且隐约可见，但太小而无法测量，则在这种情况下也应分配默认值5mm）。此默认值源自5mm CT切片厚度（但不应随 CT切片厚度的变化而改变）。这些病灶的测量可能是不可重复的，因此提供默认值将防止基于测量误差的假缓解或假进展。然而，重申一下，如果放射科医生能够提供实际的测量结果，即使它低于5毫米，也应该记录下来。

**治疗中分裂或融合的病灶 (Lesions that split or coalesce on treatment)**

如附录Ⅱ所述，当非淋巴结病灶变成“碎片”时，应将碎片部分的最长直径相加以计算靶病灶总和。同样，当病灶融合时，可以在它们之间保持一个平面，以帮助获得每个单个病变的最大直径测量值。如果病变确实已经融合，以至于不可分离，在这种情况下，最长直径应该是“融合病灶”的最长直径的最大值。

**4.3.3. 非靶病灶的评估**

本节提供用于确定一组非靶标病灶的肿瘤疗效标准的定义。虽然一些非靶病灶实际上可能是可测量的，但它们不需要测量，而应仅在方案指定的时间点进行定性评估。

- **完全缓解（Complete Response, CR）：**
  所有非靶病灶消失，肿瘤标志物水平正常化。所有淋巴结的大小必须是非病理性的（短轴<10mm） 。
- **Non-CR/Non-PD**： 一个或多个非靶病灶持续存在和/或肿瘤标志物水平维持在正常值上限以上。
- **疾病进展（Progressive Disease, PD）：** 现有非靶病灶的明确进展（见下文评论）。（注意：一个或多个新病灶的出现也被认定为是进展）。

**4.3.4. 非靶病变进展评估的特别说明**

非靶病变进展的概念需要以下额外解释：

**当患者还有可测量的病变时**

在这种情况下，为了在非靶病变的基础上实现“明确进展”，非靶病变的总体水平必须显著恶化，这样，即使在靶病变中存在SD或PR，肿瘤总体负荷也已增加到足以值得停止治疗（请参阅附录Ⅱ中的示例以及下面的更多详细信息）。一个或多个非靶病灶的大小尺寸的适度“增加”通常不足以达到明确进展状态。因此，面对靶病灶的SD或PR，仅根据非靶病灶的变化来确定总体进展是极其罕见的。

**当患者只有不可测量病变时**

当研究入组标准不是具有可测量病变时，这种情况会出现在某些Ⅲ期试验中。如上所述，一般同样的概念在这里也适用，但是，在这种情况下，没有可测量的病变评估用于解释不可测量疾病负荷的增加。由于非靶病变的恶化不容易量化（根据定义：如果所有病变确实不可测量），因此在评估患者的明确进展时可以应用的有效检测是，考虑基于不可测量病变变化的总体疾病负担的增加，是否与宣布PD为可测量疾病所需的增加相当：即肿瘤负荷的增加意味着“体积”额外增加73%（相当于可测量病变的直径增加20%）。例如，胸腔积液从“少量”增加到“大量”，淋巴管炎疾病从局部增加到广泛，或者在方案中可能被描述为“足以需要改变治疗”。附录Ⅱ的图5和图6显示了一些说明性示例。如果看到“明确进展”，则应考虑患者此时达到整体PD。虽然最好有适用于不可测量疾病的客观标准，但这种疾病的性质使其无法这样做，因此增加必须是实质性的。

**4.3.5. 新病灶**

新的恶性病灶的出现意味着疾病进展，因此，一些关于新病灶检测的讨论很重要。没有识别新发病灶的具体影像学标准；然而，新病变的发现应该是明确的：即不能归因于扫描技术的差异、成像方式的变化或被认为代表肿瘤以外的其他东西的发现（例如，一些“新”骨病灶可能只是先前存在的病灶的愈合或耀斑）。当患者的基线病灶显示部分或完全缓解时，这一点尤其重要。例如，肝脏病变的坏死可能在CT扫描报告中报告为“新”囊性病灶，但事实并非如此。

在随访研究中发现一个解剖位置的病灶在基线时未扫描，可以被认定是新病灶，并表明疾病进展。这方面的一个例子是患者在基线时患有内脏病变，需在研究中做脑部CT或MRI以揭示转移灶的情况。患者的脑转移被认为是PD的证据，即使他/她在基线时没有进行脑成像。

如果一个新病灶是模棱两可的，例如由于其体积小，持续的治疗和随访评估将说明它是否代表真正的新疾病。如果重复扫描确认肯定有新病灶，则应使用初始扫描的日期作为进展的日期。

虽然FDG-PET反应评估需要额外的研究，但有时在评估进展时（特别是可能的“新”病变），使用FDG-PET扫描来补充CT扫描是合理的。基于FDG-PET成像的新病灶可以根据以下算法识别：

- a. 基线时FDG-PET阴性，随访时FDG-PET阳性是一个基于新病灶的PD信号。
- b. 基线时无FDG-PET，随访时FDG-PET阳性：

- 如果随访时FDG-PET阳性对应于CT确认的新病变部位，则为PD。

- 如果随访时FDG-PET阳性未被CT确认为病变新部位，则需要进行额外的随访CT扫描，以确定该部位是否确实发生进展（如果是，PD的日期将是FDG-PET初始异常的扫描日期）。

- 如果随访时FDG-PET阳性对应于CT上预先存在的病变部位，而根据解剖影像没有进展，则不是PD。

**4.4. 最佳总体疗效的评估 (Evaluation of best overall response)**

考虑到任何确认要求，最佳总体疗效是从研究治疗开始到治疗结束记录的最佳疗效。有时，直到治疗结束后才记录疗效，因此在确定最佳总体疗效时应考虑治疗后的评估，方案应明确这一点。方案必须说明在进展前引入的任何新疗法将如何影响最佳疗效的判定。患者的最佳疗效的评估将依赖于靶病灶和非靶病灶的发现，也将考虑新病灶的出现。此外，根据研究的性质和方案要求，可能还需要确认测量值（见第4.6节）。具体而言，在以疾病缓解为主要终点的非随机试验中， PR或CR需要确认后才能认为是“最佳总体疗效”。下文将对此作进一步说明。

**4.4.1. 时间点疗效 (Time point response)**

假设在方案指定的每个时间点，都会进行一次疗效评估。下一页的表1总结了在基线时有可测量病灶的患者，在每个时间点的总体疗效 (overall response) 的状态计算。

表1. 时间点反应：基线有目标（+/-非目标）疾病的患者。

| Target lesions | Non-target lesions | New lesions | Overall response |
| --- | --- | --- | --- |
| CR | CR | No | CR |
| CR | Non-CR/non-PD | No | PR |
| CR | Not evaluated | No | PR |
| PR | Non-PD or not all evaluated | No | PR |
| SD | Non-PD or not all evaluated | No | SD |
| Not all evaluated | Non-PD | No | NE |
| PD | Any | Yes or No | PD |
| Any | PD | Yes or No | PD |
| Any | Any | Yes | PD |

CR = 完全反应，PR = 部分反应，SD = 稳定疾病，PD = 进展性疾病，NE = 不可评估。

当患者仅有不可测量病变时（因此非靶），应使用表2。

表2. 时间点反应：只有非目标疾病的患者。

| Non-target lesions | New lesions | Overall response |
| --- | --- | --- |
| CR | No | CR |
| Non-CR/non-PD | No | Non-CR/non-PD^a^ |
| Not all evaluated | No | NE |
| Unequivocal PD | Yes or No | PD |
| Any | Yes | PD |

CR = 完全反应，PD = 进展性疾病，NE = 不可评估。

^a^ “非CR/非PD”优于非目标疾病中的“稳定疾病”，因为SD越来越多地用作评估一些试验中疗效的终点，因此在无法测量任何病变时分配此类别是不建议的。

**4.4.2. 缺少评估与不可评估的说明**

当在特定时间点完全没有影像/测量时，患者在该时间点是不可评估的(NE)。如果在评估中只测量了部分病灶，该病例在该时间点通常也被认为是NE，除非有令人信服的依据，即单个缺失病灶的贡献不会改变指定时间点的疗效。这最有可能发生在疾病进展的病例中，例如，如果一个患者三次测量的基线总和为50mm，在随访时仅评估了两个病灶，但这些评估的总和为80mm，无论缺失病灶的贡献如何，患者都将达到PD状态。

**4.4.3. 最佳总体疗效：所有时间点 (Best overall response: all time points)**

一旦获取患者的所有数据，就可以确定最佳总体疗效。

**在不需要确认完全或部分缓解的试验中，最佳疗效的确定：**

这些试验中的最佳疗效被定义为所有时间点中的最佳疗效（例如，病人第一次评估为疾病稳定(SD)，第二次评估为部分缓解(PR)，最后一次评估为疾病进展(PD)，则最佳总体疗效为部分缓解(PR)）。当疾病稳定(SD)被认为是最佳疗效时，它也必须满足方案规定的从基线开始的最小时限。如果不满足最小时限，疾病稳定(SD)就不会被认为是最佳疗效，患者的最佳疗效就取决于随后的评估。例如，病人第一次评估为疾病稳定(SD)，第二次为疾病进展(PD)，并且不满足疾病稳定(SD)的最小持续时间，则其最佳疗效为疾病进展(PD)。第一次评估为疾病稳定(SD)后未能随访的患者，将被认为是不可评估的。

**在需要确认完全或部分缓解的试验中，最佳疗效的确定：**

只有在方案规定的后续时间点满足对应标准（通常是4周后），才能确认完全缓解(CR)或部分缓解(PR)。在这种情况下，最佳总体疗效如表3中说明。

表3. 当确认CR和PR需要时的最佳总体反应。

| Overall response | Overall response | BEST overall response |
| --- | --- | --- |
| First time point | **Subsequent time point** | Empty Cell |
| CR | CR | CR |
| CR | PR | SD, PD or PR^a^ |
| CR | SD | SD provided minimum criteria for SD duration met, otherwise, PD |
| CR | PD | SD provided minimum criteria for SD duration met, otherwise, PD |
| CR | NE | SD provided minimum criteria for SD duration met, otherwise NE |
| PR | CR | PR |
| PR | PR | PR |
| PR | SD | SD |
| PR | PD | SD provided minimum criteria for SD duration met, otherwise, PD |
| PR | NE | SD provided minimum criteria for SD duration met, otherwise NE |
| NE | NE | NE |

a 如果在第一次时间点真正满足CR，则在随后的时间点看到的任何疾病，即使是与基线相比满足PR标准的疾病，也会使疾病在该时间点PD（因为疾病必须在CR后重新出现）。最佳反应将取决于是否满足SD的最短时间。然而，有时可能会声称“CR”，而随后的扫描表明小病变可能仍然存在，实际上患者在第一次时间点有PR，而不是CR。在这种情况下，原来的CR应该改为PR，最佳反应是PR。

**4.4.4. 疗效评估的特别说明**

当淋巴结病变包括在靶病灶的（直径）总和中，并且淋巴结减小到“正常”大小(<10mm)时，其仍然可能在扫描报告中有测量结果。即使结点是正常的，这种测量也应该记录下来，避免夸大基于结点大小增加的疾病进展。如前所述，这意味着CR患者的病例报告表(CRF)上的直径总和可能不是“零”。

在需要确认缓解的试验中，重复的“NE”时间点评估可能会使最佳疗效的确认复杂化。试验分析计划必须解决，在确定缓解和进展时，如何处理缺失的数据/评估。例如，在大多数试验中，将具有时间点疗效PR-NE-PR的患者视为确认缓解是合理的。

如果患者健康状况总体恶化，需要停止治疗而当时没有疾病进展的客观证据，则应报告为“症状恶化”。即使在停止治疗后，也应尽一切努力记录客观的进展情况。症状恶化不是客观疗效的描述：只是停止研究治疗的一个原因。这类患者的客观疗效状态，将通过对靶病变和非靶病变的评估来确定，见表1-3。

定义“早期进展、早期死亡和不可评估”的条件是各研究所特有的，应该在方案中明确说明（取决于治疗持续时间，治疗周期）。

在某些情况下，区分残余病灶和正常组织可能会很困难。当评估完全缓解取决于这一决定时，建议在确认完全缓解状态之前对残留病灶进行检查(细针穿刺/活检)。在残余影像学异常被认为代表纤维化或瘢痕形成的情况下，FDG-PET可用于将疗效改善至CR，其方式类似于活检。在这种情况下，FDG-PET的使用应在方案中进行前瞻性描述，并应得到适应症对应的特定疾病医学文献的支持。然而，必须承认，由于FDG-PET和活检分辨率/敏感性的局限性，这两种方法都可能导致假阳性CR。

对于疾病进展不明确的发现（例如，非常小和不确定的新病灶；囊性病灶或已有病灶的坏死），治疗可持续到下一次的计划评估。如果在接下来的计划评估中，疾病进展得到确认，则疾病进展日期应以怀疑疾病进展的较早日期为准。

**4.5 肿瘤重新评估的频率**

治疗期间肿瘤重新评估的频率应具有方案特异性，并适应治疗类型和方案。然而，在治疗的有益效果尚不清楚的Ⅱ期研究中，每6-8周随访一次是合理的（时间安排在周期结束时）。在特定的治疗方案或情况下，比这些时间间隔更小或更大可能是合理的。该方案应指定在基线时评估哪些器官部位（通常是那些最有可能与所研究肿瘤类型的转移性疾病有关的器官部位）以及重复评估的频率。通常，所有靶和非靶部位在每次评估时间点都要进行评估。在某些情况下，对某些非靶器官的评估可能不那么频繁。例如，仅在靶病变确认完全缓解或怀疑骨骼疾病进展时，骨扫描才需要重复进行。

治疗结束后，是否需要重复评估肿瘤，取决于试验目标是缓解率还是事件发生的时间(进展/死亡)。如果“到某一事件的时间”(例如，疾病进展时间、无病生存期、无进展生存期)是研究的主要终点，则有必要对方案指定的疾病部位进行常规的计划性重新评估。特别是在随机比较试验中，计划的评估应按照时间表进行（例如：治疗后每6-8周或治疗后每3-4个月一次），并且不应受到治疗延迟、药物假期或任何其他可能导致治疗组疾病评估时间不平衡的事件影响。

**4.6. 确认测量/缓解持续时间**

**4.6.1. 确认 (Confirmation)**

在疾病缓解作为主要终点的非随机试验中，需要确认PR和CR，以确保疾病缓解不是测量误差的结果。这也将允许在历史数据的背景下对结果进行适当的解释，在这些试验中，按传统疾病缓解需要确认（见Bogaerts等人在本期特刊中的论文10）。然而，在所有其他情况下，即在随机试验（Ⅱ期或Ⅲ期）或以疾病稳定或疾病进展为主要终点的研究中，不需要确认缓解，因为它不会增加对试验结果的解释。然而，特别是在非盲法研究中，为防止偏倚，取消对疾病缓解的确认可能会增加中心审查的重要性。

在SD的情况下，必须在研究开始后，至少有一次测量符合研究方案中定义的最小时限的SD标准(通常不少于6-8周)。

**4.6.2. 总缓解期 (Duration of overall response)**

总缓解期是指，从首次满足CR/PR测量标准的时间（以首次记录者为准）到客观记录的疾病复发或疾病进展的第一个日期（以研究中记录的最小测量值作为疾病进展的参考）的持续时间。

完全缓解期是指，从首次满足CR测量标准的时间到客观记录疾病复发的第一个日期的持续时间。

**4.6.3. 疾病稳定期 (Duration of stable disease)**

疾病稳定期是指，从治疗开始（在随机化试验中，从随机化日期开始），到符合疾病进展标准的持续时间，疾病进展以研究中的直径和的最小值作为参照（如果基线总和最小，基线总和就是计算PD参考值）。

疾病稳定持续时间的临床相关性在不同的研究和疾病中有所不同。如果在特定试验中，在最短时间内达到疾病稳定的患者比例是重要的终点，则方案应指定两次测量之间确定疾病稳定所需的最小时间间隔。

注：缓解期、稳定期及与无进展生存期会受到基线评估后随访频率的影响。定义标准随访频率不在本指南的范围内。确定随访频率应考虑多个参数，包括疾病类型和阶段、治疗周期和标准操作。然而，如果要在试验之间进行比较，应考虑测量终点精度的这些限制。

**4.7. 无进展生存期/无进展比率**

**4.7.1. Ⅱ期试验**

本指南主要侧重于Ⅱ期试验中客观缓解终点的使用。在某些情况下，“缓解率”可能不是评估新药物/新方法潜在抗癌活性的最佳方法。在这种情况下，“无进展生存期”（PFS）或具有里程碑意义的时间点的“无进展比例”可能被认为是适当的替代方案，以提供新药物生物学效应的初始信号。然而，很明显，在非对照试验中，这些措施会受到批评，因为明显有希望的观察结果可能与患者选择等生物学因素有关，而不是干预的影响。因此，利用这些终点的Ⅱ期筛查试验最好采用随机对照设计。如果某些癌症的行为模式非常一致（并且通常一直很差），因此非随机试验是合理的，则可能存在例外情况（例如参见van Glabbeke等人20）。然而，在这些情况下，在没有治疗效果的情况下，必须仔细记录估计预期PFS或无比例进展的基础。

**4.7.2. Ⅲ期试验**

晚期癌症的Ⅲ期临床试验，越来越多地将评估无进展生存期 (PFS) 或至肿瘤进展时间 (TTP) 作为主要研究终点。如果试验方案要求所有患者都有可测量的病灶，疾病进展评估就相对简单。但是，限制患者入组会遭受批评：如果在研究的疾病中，大量患者被排除在外，那么可能会导致试验结果不太具有普遍性。此外，限制入组将减缓研究的受试者招募。所以，在越来越多的试验中，可测量病灶的患者与只有不可测量病灶的患者都被允许入组。在这种情况下，对于那些没有可测量病灶的患者，必须仔细明确地描述使其符合疾病进展的发现条件。此外，在这种情况下，方案必须说明可测量病变患者记录的靶病灶的最大数量是否可以从五个减少到三个 (基于Bogaerts等人10和Moskowitz等人11发现的数据)。正如在“疾病进展评估的特别说明”中描述的那样，这些指南为这种情况下的疾病进展评估提供了建议。此外，如果可行，经过验证的疾病进展的肿瘤标记物测量可能有助于整合到疾病进展的定义中（已被提议用于卵巢癌）。如果要根据研究结果做出重要的药物开发或药物批准决定，可能需要对影像学研究或影像学原始报告进行集中盲法审查，以验证“明确进展”。最后，如前所述，由于进展日期存在确定性偏倚，研究组的调查时间应相同。Dancey等人在本期特刊21上发表的文章对随机试验疾病进展的评估进行了更详细的讨论。

**4.8.缓解和进展的独立审查**

对于以客观缓解(CR + PR)为主要终点的试验，特别是关键药物开发决策基于对最少数量的应答者的观察，建议所有得出的疗效应由独立于研究的专家进行审查。如果该研究是随机试验，理想情况下，审查员应该对治疗分配不知情。最好的方法是同时审查患者档案和放射学影像。

对疾病进展的独立审查引出了一些更复杂的问题：例如，使用基于中心审查的疾病进展时间，代替基于调查人员的疾病进展时间，存在统计问题。因为当前者先于后者时，可能会引入信息审查。Ford等人在本期特刊的一篇文章中概述了这些因素以及从独立审查中获得的其他经验教训22。

**4.9.报告最佳疗效结果**

**4.9.1. Ⅱ期临床试验**

当疗效是主要终点，因此所有患者必须有可测量的病变才能进入试验时，即使存在重大的方案治疗违背或者不可评估，研究中包括的所有患者都必须在结果报告中予以考虑。 每位患者将被划分为以下类别之一：

1. 完全缓解 (Complete response)
2. 部分缓解 (Partial response)
3. 疾病稳定 (Stable disease)
4. 疾病进展 (Progression)
5. 疗效不可评估 (Inevaluable for response)：特定原因（例如：因肿瘤而早期死亡；因毒性早期死亡；肿瘤评估资料不能重复或不完全；其他（特定））

通常，所有符合条件的患者都应包括在分母中，以计算Ⅱ期试验的缓解率（在某些方案中，包括所有治疗的患者是合适的）。对于计算出的缓解率，通常最好给出95%的双侧置信限。试验结论应基于所有符合条件（或所有接受治疗的）患者的缓解率，而不应基于选定的“可评估的”子集。

**4.9.2. Ⅲ期临床试验**

Ⅲ期试验中的疗效评估可能是所评估治疗的相对抗肿瘤活性的指标，并且几乎总是次要终点。观察到的缓解率的差异，可能并不能预测研究人群的临床相关治疗效益。如果选择客观缓解作为Ⅲ期研究的主要终点（只有在肿瘤客观缓解和临床相关治疗效益之间有直接关系的情况下，才可以明确地为研究人群证明），应采用与Ⅱ期试验相同的标准，所有进入试验的患者都应该至少有一个可测量的病灶。

在许多情况下，缓解是次要终点，并非所有试验患者都有可测量的病变，报告总体最佳缓解率的方法必须在方案中预先指定。在实践中，可以使用“治疗意向”分析（分母中的所有随机患者）或仅包括基线时可测量病变患者的亚组分析来报告缓解率。方案应该清楚地规定如何报告疗效结果，包括计划的任何亚组分析。

RECIST的原始版本建议，在Ⅲ期试验中，人们可以使用对RECIST指南的“宽松”解释来编写方案（例如，减少测量的病灶数量），但不应再这样做，因为这些修订后的指南已经修订，明确了这些标准应如何应用于所有以肿瘤缓解或进展的解剖学评估为终点的试验。
